# Towards a Consensus for the Analysis and Exchange of TFA as a Counterion in Synthetic Peptides and Its Influence on Membrane Permeation

**DOI:** 10.3390/ph18081163

**Published:** 2025-08-05

**Authors:** Vanessa Erckes, Alessandro Streuli, Laura Chamera Rendueles, Stefanie Dorothea Krämer, Christian Steuer

**Affiliations:** 1 Pharmaceutical Analytics, Department of Chemistry and Applied Biosciences, ETH Zurich, 8093 Zurich, Switzerland; vanessa.erckes@pharma.ethz.ch (V.E.); alessandro.streuli@pharma.ethz.ch (A.S.);; 2 Biopharmacy, Department of Chemistry and Applied Biosciences, ETH Zurich, 8093 Zurich, Switzerland; stefanie.kraemer@pharma.ethz.ch

**Keywords:** counterion, trifluoroacetic acid, 19F-NMR, HPLC-ELSD, FT-IR, membrane permeability

## Abstract

**Background:** With the increasing shift in drug design away from classical drug targets towards the modulation of protein-protein interactions, synthetic peptides are gaining increasing relevance. The synthesis and purification of peptides via solid-phase peptide synthesis (SPPS) strongly rely on trifluoroacetic acid (TFA) as a cleavage agent and ion-pairing reagent, respectively, resulting in peptides being obtained as TFA salts. Although TFA has excellent properties for peptide production, numerous studies highlight the negative impact of using peptides from TFA^−^ salts in biological assays. **Methods:** Investigated peptides were synthesized via SPPS and the TFA^−^ counterion was exchanged for Cl^−^ via freeze-drying in different concentrations of HCl. Detection and quantification of residual TFA^−^ were carried out via FT-IR, ^19^F-NMR, and HPLC using an evaporative light-scattering detector (ELSD). A liposomal fluorescence assay was used to test for the influence of the counterion on the peptides’ passive membrane permeability. **Results:** All TFA^−^ detection methods were successfully validated according to ICH guidelines. TFA^−^ removal with 10 mM HCl was determined to be the optimal condition. No impact on peptide purity was observed at all HCl concentrations. Influences on permeability coefficients depending on peptide sequence and salt form were found. **Conclusions:** This study presents a systematic investigation of the removal of TFA^−^ counterions from synthetic peptides and their replacement with Cl^−^ counterions. Detected counterion contents were used to understand the impact of sequence differences, especially positive charges, on the amount and potential localization of counterions. Our findings emphasize the importance of counterion quantification and specification in assays with synthetic peptides.

## 1. Introduction

Peptides, which are chemical entities, are an emerging class of therapeutic drug forms located in between the chemical spaces of small-molecule drugs and biologics. Compared to these two classes, peptides offer unique therapeutic opportunities by mimicking known signaling molecules such as hormones and cytokines [1]. Insulin, the first peptide-based therapeutic, demonstrated the life-saving potential of peptide drugs in humans when it was first used as a hormone replacement therapy in the early 1920s [2]. Even though this was a medical breakthrough that helped a large group of patients and insulin is still used as a drug to this day, these types of natural, isolated peptides were found to have limitations as therapeutic molecules, such as their poor in vivo stability and low membrane permeability, including poor oral absorption [3,4].

With the development of solid-phase peptide synthesis (SPPS) in 1963 by Merrifield, the reliable and rapid synthesis of longer and chemically modified peptide chains has been revolutionized, allowing researchers to take a medicinal chemistry approach to help overcome the existing challenges [1,5]. Merrifield’s original approach consisted of repeating cycles of coupling, washing, and deprotection, resulting in the addition of Boc *N*-protected amino acids to a growing peptide chain that was covalently bound to a resin. This process required the use of strong acids like hydrogen fluoride (HF) for global deprotection. Due to the high toxicity of HF and the demanding safety requirements, an alternative synthesis route was needed. The introduction of the Fmoc protection group in SPPS addressed this challenge, as it offered the possibility of an orthogonal synthesis strategy and, therefore, the replacement of HF with trifluoroacetic acid (TFA), which is less hazardous and more widely available [6,7]. Since the development of this alternative route, TFA, as a global deprotection and cleavage agent, has become indispensable. Furthermore, TFA is also widely employed as an ion-pairing reagent in reverse-phase liquid chromatography (RP-LC) for both purification and analytical characterization [4,8]. In this application, TFA^−^ acts as a strong ion-pairing reagent, presumably with positively charged basic functional groups, as found on the *N*-terminus and in the amino acids arginine, histidine, and lysine, that improves the separation of polar compounds on lipophilic stationary phases. Due to the strong interaction between TFA^−^ and the peptide, the lyophilization of peptide-containing solutions results in the formation of a peptide—TFA salt.

Despite the central role of TFA in peptide chemistry, there is increasing awareness that TFA, when used as a counterion, can interfere with physicochemical, in vivo, and in vitro experiments [9]. From a chemical perspective, TFA^−^ can introduce a weighting error due to differences in the molecular weights of its salt forms [10,11], there can be changes in peptide conformation [12], and challenges arise due to complicated physicochemical characterization, such as the determination of the secondary structure via CD spectroscopy and FT-IR [13,14]. In in vitro and in vivo settings, residual TFA^−^ has been shown to increase, as well as inhibit, cell proliferation, increase cell and liver toxicity, and lead to greater activation of antibody responses [13,15,16,17,18,19,20,21]. Additionally, multiple studies have shown that masking hydrogen bonds in peptides can increase membrane permeability [22,23,24]. TFA^−^ is able to significantly shield the polarity of peptides when used as an ion-pair reagent in HPLC systems, as seen by an increase in retention time and, therefore, lipophilicity [8]. This raises the question as to whether the presence of TFA^−^ as a counterion translates to increased membrane permeability [25,26]. In regard to the multifaceted influences of TFA on peptide properties, the removal of TFA^−^ or its replacement with a more physiological ion such as chloride (Cl^−^) or acetate should always be considered for synthetic peptides. An in-depth discussion on the role of different types of counterions in peptides can be found elsewhere [9]. Various exchange techniques have been described, including anion-exchange columns, RP-HPLC with varying counterion concentrations, cycles of deprotonation and reprotonation, or treatment with stronger acids such as hydrochloric acid (HCl) and subsequent lyophilization [17,27]. The exchange of TFA^−^ to Cl^−^ using HCl followed by lyophilization is likely the most convenient and widely adopted protocol. However, no consensus has been established regarding the optimal HCl concentration (ranging from 2 to 100 mM), the number of exchange cycles (typically 1–5), or the potential impact of the procedure on peptide integrity [13,27]. Similarly, various methods for the detection of TFA^−^ including RP-HPLC-UV, ^19^F-NMR, ATR FT-IR, ion chromatography, and HPLC coupled to an evaporative light—scattering detector (ELSD) are described, but reported methods often lack validation according to ICH guidelines and often only report TFA^−^ content without assessing the respective exchanged ion [10,13,14,17,28,29].

**Figure 1:**
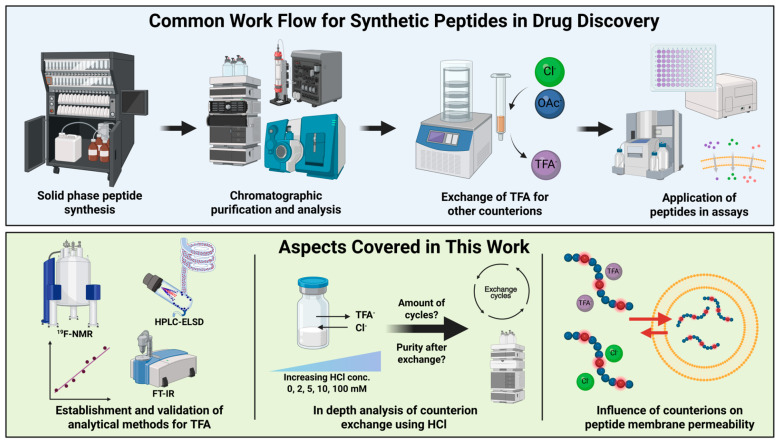
(**Top**) Graphical representation of common workflows implemented in the generation, analysis, and testing of synthetic peptides. (**Bottom**) Aspects covered in this publication regarding detection, exchange, and role of counterions in passive membrane permeation. Created in BioRender (Steuer, C. (2025) https://BioRender.com/zqqq2q2).

In this study, we aimed to define a consensus on optimal conditions for the commonly used HCl exchange and appropriate methods for the monitoring of the residual TFA^−^ (Figure 1). For this, the two commonly used detection techniques ^19^F-NMR and FT-IR were validated according to the ICH guidelines [30,31]. Further, our previously described ELSD method was optimized for the detection of TFA^−^ and Cl^−^ counterions [10]. Using angiotensin (AT) 1 as a model peptide, we evaluated varying HCl concentrations (0–100 mM) to identify the most suitable conditions for the exchange and monitored the TFA^−^ and Cl^−^ concentrations over multiple cycles. Optimal conditions were applied to peptides AT 2–4 as well as the cell-penetrating peptides (CPPs) Pep-1 [32,33], pVEC [34], and penetratin (AntP) [35,36]. Finally, we investigated whether the presence of TFA^−^ affects passive membrane permeability using a liposomal fluorescence assay which is able to measure the kinetics of passive permeation of ionizable compounds across lipid bilayers without the need for compound labelling [37,38].

## 2. Results and Discussion

### 2.1. Method Development, Validation, and Comparison for TFA Quantification

The analytical methods FT-IR, HPLC-ELSD, and ^19^F-NMR were developed and validated for the detection and quantification of TFA^−^. Table 1 provides a comparative overview of their key characteristics and validation performance, including potential strengths and limitations. The validation of all methods was conducted in accordance with the criteria outlined in the ICH guidelines. FT-IR spectra consistently showed key absorption bands at 1670, 1200, and 1147 cm^−1^. In accordance with previous reports, the 1200 cm^−1^ C-F stretch proved to be most suitable for the quantification of TFA^−^ due to its prominence and consistency [39]. Established calibration curves showed linearity in the covered quantitative range as well as a good fit with R^2^ > 0.99. The overall bias was <5% for QC med and high samples and <20% for QC low samples (Table 1). However, single values of QC low samples displayed high biases (e.g., +55.7%), suggesting low accuracy at low concentrations. Nonetheless, the intra- and inter-day precision (RSD_R_ and RSD_T_) of the QC high and med samples were <15% and for QC low <20%, indicating acceptable imprecision at medium and high levels. For HPLC-ELSD measurements, several optimization steps of a previously published method were applied. A stable chromatographic baseline was achieved by reducing the buffer concentration to 20 mM (pH = 4.0), allowing us to lower the ELSD temperature to 30 °C. The adapted solvent system allowed isocratic separation with a reduced run time of 10 min. Using the optimized method, regression curves for each analyte were determined using a quadratic model resulting in an R^2^ > 0.99 for all [40,41]. Bias was <10% for all analytes and concentrations except for the QC low of TFA^−^ displaying a bias of <15%. Precision data for all analytes fulfilled the ICH criteria and were <10% for RSD_R_ and RSD_T_. The ^19^F-NMR method was adapted from previous reports to suit peptide samples [29,42]. The previously used solvents dimethyl sulfoxide-d_6_ and methanol-d_4_ were replaced with D_2_O:H_2_O (1:9) which is frequently used for peptide analysis by NMR. Calibration curves were established using a linear model with a weighting of 1/x displaying an R^2^ > 0.99. An overall bias of <10% and excellent precision with RSD_R_ and RSD_T_ values of <3% across all QC levels was determined for the ^19^F-NMR method.

**Figure 2:**
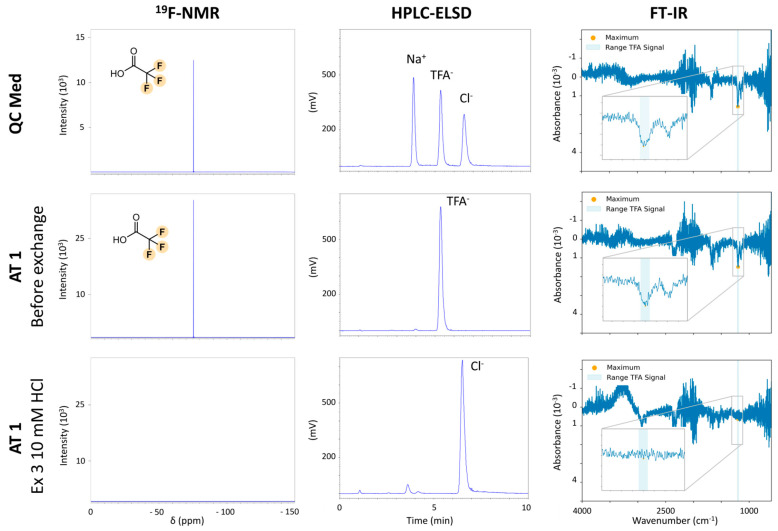
Exemplary spectra and chromatograms of the three developed and validated techniques for the identification and quantification of TFA^−^. From left to right the ^19^F-NMR, HPLC-ELSD, and FT-IR are displayed. From top to bottom for each technique, a QC medium and peptide sample, here AT 1, before and after counterion exchange are shown. Analytical data of other peptides can be found in Supplementary Materials Section S3.

In Figure 2, the top row shows representative QC med measurements across all platforms. Both ^19^F-NMR and HPLC-ELSD offered reliable quantification, with ^19^F-NMR showing slightly better precision, while HPLC-ELSD allowed simultaneous detection of Cl^−^ and sodium (Na^+^) and offered a lower limit of quantification (LoQ) (1.52 µg/mL HPLC-ELSD vs. 20.68 µg/mL for ^19^F-NMR). In contrast, FT-IR exhibited high variability and a comparatively high LoQ of 713 µg/mL, limiting the use for quantitative analysis of TFA^−^. However, due to its simple and rapid implementation, FT-IR remains a useful option for qualitative analysis to confirm the presence of TFA^−^ in peptide samples. As proof of principle, AT 1 was analyzed before and after counterion exchange using all three methods. No signal shifts or matrix interferences were observed as shown in Figure 2.

### 2.2. Testing Different Counterion Exchange Protocols

**Figure 3:**
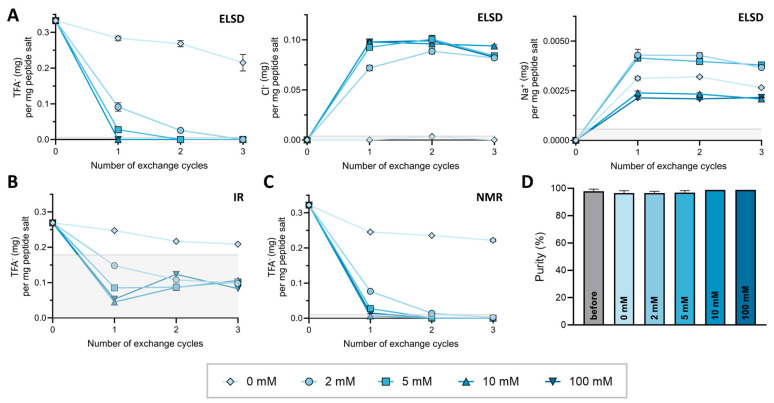
Determination of counterion content in AT 1 peptide salt after HCl exchange in different concentrations of aq. HCl between 2 mM and 100 mM as well as the control indicated by symbol and color legend. LoQ thresholds for TFA^−^, Cl^−^, and Na^+^ (mg) per mg peptide salt analyzed are indicated by a light gray area. Some LoQ thresholds are not visible. Standard deviations are indicated as error bars; however, they are not visible for all data points as they are too small to distinguish from the symbols. (**A**) Simultaneous quantification of TFA^−^, Cl^−^ and Na^+^ by HPLC-ELSD (n = 3). (**B**,**C**) TFA^−^ content determined by FT-IR (n = 1) and ^19^F-NMR (n = 3), respectively. (**D**) Purity of AT 1 determined by HPLC-UV before and after 3 exchange cycles (n = 3).

Using AT 1 as a model peptide, several HCl concentrations, (0–100 mM) for counterion exchange from TFA^−^ to Cl^−^ were tested. The TFA^−^ content was quantified using the three validated methods after purification and after each lyophilization cycle. To compare results across platforms and account for dilution differences, TFA^−^ content was normalized to 1 mg of peptide salt. The TFA^−^ content for each technique and concentration of aq. HCl for exchange over three exchange cycles are shown in Figure 3A–C. The calculated LoQs per mg peptide salt (HPLC-ELSD = 5.8 µg, ^19^F-NMR = 10.3 µg and FT-IR = 178 µg) are indicated in gray. All raw data as well as an exemplary spectrum for each condition are provided in Supplementary Materials Section S2.

After purification, AT 1 contained 0.333 ± 0.008 mg TFA^−^ per mg peptide salt as determined by HPLC-ELSD. Exchange using 10 and 100 mM HCl reduced the TFA^−^ content below the LoQ of all analytical techniques after one exchange cycle, indicating a TFA^−^ content below 1% (*w*/*w*). For 5 mM HCl and 2 mM HCl, this threshold was reached after two and three exchange cycles, respectively. The exchange efficiency increased with higher HCl concentration, whereas no substantial difference could be found between HCl concentrations of 10 and 100 mM. Interestingly, in the absence of HCl (0 mM control) a reduction in TFA^−^ content was also observed, suggesting the presence of excess unbound TFA^−^ removed by repeated lyophilization. The final TFA^−^ content in the control was 0.215 ± 0.023 mg TFA^−^ per mg peptide salt after three exchange cycles. Detected contents were comparable across the three techniques. 

As the presented HPLC-ELSD method not only allows the detection of TFA^−^ but also Cl^−^ and Na^+^, we were able to monitor the removal of TFA^−^ as a counterion while simultaneously observe an increase in Cl^−^. As shown in Figure 3A, the Cl^−^ concentration increased proportionally to the reduction in TFA^−^ content across each cycle. For example, AT 1 exchanged with 10 mM HCl reached a Cl^−^ content of 0.098 mg per 1 mg peptide salt after a single cycle, corresponding to 99.0% of the expected value based on stochiometric exchange according to Table 2, with no further increase. The content determination of Na^+^ in the samples resulted in overall concentrations below 0.005 mg per 1 mg peptide (<0.5% (*w*/*w*)) and remained constant from the first to the last lyophilization cycle for all samples. A possible source of Na^+^ could not be identified.

As degradation issues in aqueous acidic solutions have been reported previously [45], the integrity of AT 1 during counterion exchange with the different concentrations of HCl was measured with HPLC-UV. The purity of AT1 for all concentrations of HCl over three exchange cycles was constantly above 96% as shown in Figure 3D. No degradation products were observed HPLC-UV. Similar results could be found for all other peptides under investigation (Supplementary Materials Section S3). Therefore, it can be concluded that diluted HCl up to 100 mM does not compromise peptide integrity. Based on these results, 10 mM HCl was determined as the optimal condition and used for subsequent TFA^−^ exchanges. Nevertheless, the stability of the peptide as well as the counterion exchange efficacy may vary, depending on the peptide sequence. Therefore, the TFA^−^ content as well as the purity should always be confirmed after counterion exchange.

### 2.3. Effect of Peptide Sequence on Counterion Exchange

**Figure 4:**
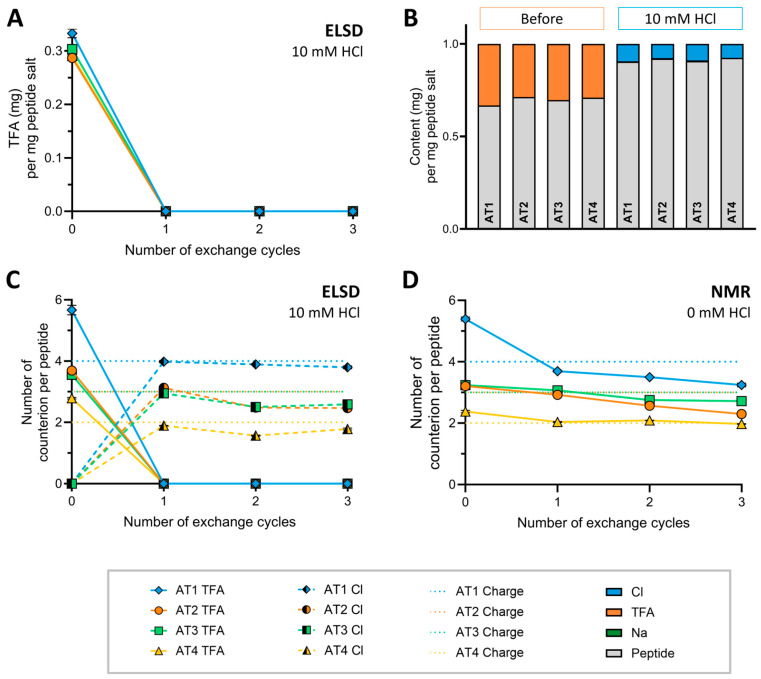
(**A**) Mean (n = 3) TFA^−^ content in AT 1–4 (as indicated by color and symbol code) determined by HPLC-ELSD before and after counterion exchange with 10 mM HCl. Standard deviations are indicated as error bars; however, they are not visible for all data points as they are too small to distinguish from the symbol. (**B**) Mean content (n = 3) of peptide (gray) and the counterions TFA^−^ (orange) and Cl^−^ (blue) in 1 mg peptide salt of AT 1–4 before (left) and after (right) 3 counterion exchange cycles with 10 mM HCl. Values were determined by HPLC-ELSD. Na^+^ content (green) is not visible. (**C**) Mean number (n = 3) of counterions of TFA^−^ (continuous line and fully colored) and Cl^−^ (dashed line and half-colored) calculated per peptide AT 1–4 based on HPLC-ELSD analysis after counterion exchange in 10 mM HCl. Horizontal dotted lines indicate the number of positively charged amino acids in the peptides. Standard deviations are indicated as error bars; however, they are not visible for all data points as they are too small to distinguish from the symbol. (**D**) Mean number (n = 3) of counterions of TFA^−^ calculated per peptide AT 1–4 based on ^19^F-NMR analysis after counterion exchange in the control group (0 mM HCl). Horizontal dotted lines indicate the number of positively charged amino acids in the peptides. Standard deviations are indicated as error bars; however, they are not visible for all data points as they are too small to distinguish from the symbol.

To further evaluate the influence of the peptide sequence and molecule charge in the counterion exchange process, the exchange of TFA^−^ to Cl^−^ was performed for peptides AT 1–4, originally obtained as TFA salts after synthesis and purification. Counterion exchange was conducted for AT 2, 3, and 4 with the optimized conditions of 10 mM HCl over three cycles. The TFA^−^ concentration after each lyophilization cycle was monitored with FT-IR, ^19^F-NMR, and HPLC-ELSD. The results for TFA^−^ removal in AT 2–4 were comparable to AT 1 across all detection methods. After one exchange cycle with 10 mM HCl, the TFA^−^ concentrations of AT 2–4 were below the LoQ as shown for the HPLC-ELSD analysis in Figure 4A, and no impact on the purity of the peptide was determined. The results of AT 2–4 for FT-IR,^19^F-NMR, and purity determination are provided in Supplementary Materials Sections S2 and S3.

Based on these results, we tested our hypothesis that the amount of counterions corresponds to the number of positive charges in the peptides. For this, theoretical molecular weight calculations for all of the peptides listed in Table 1 were conducted assuming the stoichiometric pairing of each positive charge with a counterion at acidic pH < 3. We chose this pH range reflecting both the conditions used during purification and applied in the counterion exchange process. Based on these assumptions, the expected weight percentage for the TFA^−^ and Cl^−^ salt for AT 1–4 was determined at approximately 25% for TFA^−^ and 10% for Cl^−^, respectively. The experimentally determined counterion contents in mg per mg peptide salt for AT 1–4 before and after counterion exchange are summarized in Figure 4B. While the Na^+^ content remained <0.5%, the content of TFA^−^ reached up to 35% and the content of Cl^−^ reached up to 10% of total weight in peptide salts. These results, following up on our previous publications, highlight again the importance of taking the content of counterions of peptide samples into account [10,11].

To allow a correlation of the counterion content to the number of positive charges in each sample, we calculated the molar content of TFA^−^ and Cl^−^ enabling estimation of the number of counterions per peptide molecule as shown in Figure 4C. AT 1 had the highest number of TFA^−^ counterions per peptide followed by AT 2 and AT 3 and the lowest number for AT 4, aligning with the number of positive charges in their respective sequences. Similarly, this trend was also observed for the number of Cl^−^ per peptide after counterion exchange, showing a clear correspondence between the number of Cl^−^ per peptide and the number of positive charges. Before counterion exchange, AT 1–4 all showed a higher number of TFA^−^ ions per peptide than expected from the number of positive charges in the peptide sequence. Considering the observation of decreasing TFA^−^ content for AT 1 even when exchanging only with 0 mM HCl, counterion exchanges with 0 mM and TFA^−^ determination with ^19^F-NMR were repeated for AT 2–4. As with AT1, a decrease in TFA^−^ content was found for AT 2–4 over multiple exchanges with 0 mM HCl. As shown in Figure 4D, after the three exchange cycles, the number of TFA^−^ molecules per peptide also settles at or slightly below the expected value for stoichiometric ion pairing. To consolidate this hypothesis further in unrelated peptide sequences, the same calculations were applied to CPPs based on the TFA^−^ content determined by ^19^F-NMR before counterion exchange. The values of TFA^−^ molecules per peptide found exceeded theoretical values but reflected the expected charge-to-counterion relationship. Corresponding figures can be found in the Supplementary Materials Section S3. Concluding, synthetic peptides seem to contain excess TFA beyond the expected numbers corresponding to charge, which is not removed by initial lyophilization after purification due to strong electrostatic interactions and ion pairing. In our case, 10 mM HCl is sufficient to achieve effective and stoichiometrically appropriate counterion exchange within only one lyophilization cycle. Interestingly, in contrast to TFA^−^, no further accumulation beyond the expected number of counterions was observed for Cl^−^ in subsequent cycles. This may reflect the ability of Cl^−^ to mainly interact via ionic interactions with the peptide, whereas TFA^−^ possesses an additional hydrogen-bonding capacity [14,46]. However, the precise location and mode of interaction of TFA^−^ and residual TFA^−^ not involved in ion pairing in particular need to be investigated further. 

### 2.4. Influence of Counterions on Passive Membrane Permeation

**Figure 5:**
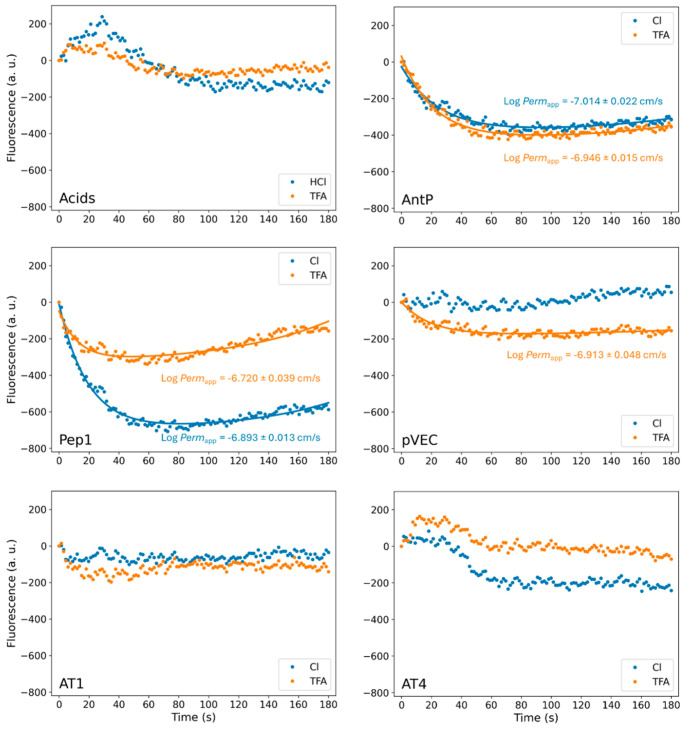
Normalized mean fluorescence curves (n = 3) in arbitrary units over time in seconds. Control measurements of the acids TFA and Cl are shown. Data of the CPPs, AT1, and AT4 are presented for both the TFA (orange) and Cl (blue) salt forms. Fitted biexponential curves and calculated Log *Perm*_app_ values in cm/s with corresponding standard error of the fit are shown if the normalized RSME of the fit was determined <0.1.

To investigate the influence of TFA^−^ and Cl^−^ as counterions on the membrane permeation of synthetic peptides, a liposomal fluorescence assay was performed as previously described [37,38]. The assay relies on the change in fluorescence of HPTS correlating to the liposomal internal pH change altered by permeation of an acidic or basic compound across the phospholipid bilayer. For basic compounds, this would result in an increase in HPTS fluorescence, while for acidic compounds the fluorescence decreases. A schematic of this principle is shown in Supplementary Materials Section S4. We first evaluated the impact of counterions on the permeation kinetics of the CPPs. The results are shown in Figure 5. Fitted biexponential curves and calculated log *Perm*_app_ values are shown if the obtained normalized RMSE was <0.10. Fitted curves with a normalized RMSE greater than 0.10 were excluded with the assumption that the biexponential model did not adequately describe the measured data. Control experiments with TFA and HCl are also shown for reference. Exact values for all fitted parameters are available in Supplementary Materials Section S4. For AntP and Pep-1, biexponential kinetics were observed for both counterion forms, while for pVEC only the TFA salt showed a measurable change in fluorescence, indicating passive membrane permeation. For the experiments where the biexponential model could be applied, the log *Perm*_app_ values ranged between −6.7 and −7.1 cm/s. The different behavior of the CPPs could be related to their distinct permeation mechanisms [47]. For AntP, both energy-dependent and -independent uptake have been described, whereby the extent of passive translocation is cell-type dependent [48,49]. In our assay, passive uptake of AntP appears to be unaffected by the type of counterion present. For Pep-1, an energy-independent mechanism involving a lipid peptide complex formation has been proposed [50]. The type of counterion in the case of Pep-1 seemed to influence the amplitude of the permeation curve, although the overall log *Perm*_app_ remained similar. In contrast, pVEC uptake is known to rely primarily on endocytosis and low passive permeation [49,51]. Consistent with this, we did not detect permeation for the Cl salt of pVEC in our assay. Interestingly, the TFA form of pVEC showed passive permeation, nevertheless. This observation might be related to the TFA property to shield polarity of the peptide mentioned earlier. It should be noted that for CPPs such as AntP and pVEC, general hemolytic activity is described [49], but for the exact concentrations as used in our assay, no POPC membrane disruption was observed [52]. For Pep-1, no hemolytic activity is reported [50]. Comparison of our findings to the literature values was complicated by the often unspecified type of peptide salt implemented in previous studies. To complement the CPP study, we also investigated peptides AT 1–4 in the permeation assay. None of these followed a biexponential function, and no Log *Perm*_app_ values could be calculated. which would indicate permeation. The type of counterion did not alter this observation. The experimental data of AT 1 and AT 4 is shown in Figure 5, while those of AT 2 and AT 3 are provided in Supplementary Materials Section S4. Considering the angiotensin receptors are located in cell membranes, it is reasonable to assume that peptides AT 1–4 are not membrane permeable, also supported by our observations [53]. However, as a limitation it should be noted that AT 1–4 have overall pIs closer to the system pH of 6.0 than the CPPs, suggesting that even if permeation occurs, no detectable pH change may be induced.

**Figure 6:**
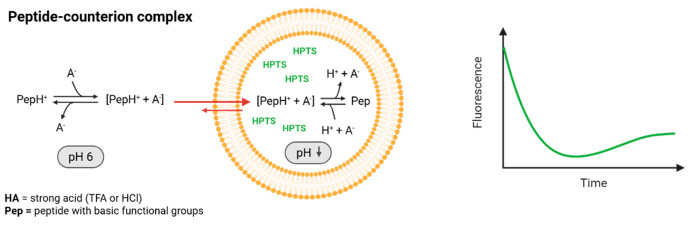
Schematic representation of the liposome assay to determine permeation kinetics over a phospholipid bilayer by monitoring the fluorescence of HPTS over time. The hypothesized mechanism involves permeation of a peptide with basic functional groups (Pep) in the presence of counterions of a strong acid such as HCl or TFA (HA). Assuming permeation of charged basic peptides occurs as a neutral peptide-counterion complex, a decrease in internal pH and therefore fluorescence over time can be observed. Created in BioRender (Steuer, C. (2025) https://BioRender.com/wg53awg).

Despite the high content of basic residues (Lys and Arg), all CPPs, except the Cl salt of pVEC, showed a decrease in fluorescence over time, indicating a decrease in internal liposomal pH. Direct pH measurements of aqueous solutions of peptides present as TFA, as well as Cl salts, were acidic in a pH range below 3 (exact values can be found in the Supporting Information). Therefore, we propose that these peptides do not cross membranes as free cations or as the very unlikely present uncharged base species, but as neutral peptide-counterion complexes as schematically shown in Figure 6. Strong counterions as Cl^−^ and TFA^−^ may shield the positive charges and hydrogen bond donors, facilitating permeation as a net neutral complex. The complex then dissociates in the unbuffered internal liposomal phase, into a weak base (peptide) and a strong acid (HCl or TFA), resulting in the release of protons leading to acidification and a decrease in fluorescence. This assumption would align with the concept of hydrophobic ion pairing, described to enhance the membrane permeability of charged molecules in drug delivery [54]. Our findings confirm results of previous studies that counterions cannot be neglected and the salt form might affect the membrane permeation behavior of peptides, among other things. Given that counterions are often unreported in the literature, we herein emphasize the need for explicit consideration and clarification in peptide-based permeation assays and further functional studies.

## 3. Material and Methods

### 3.1. Method Development and Validation for TFA^−^ Detection

The TFA^−^ content in samples was quantified using three analytical methods, namely FT-IR, ^19^F-NMR, and HPLC-ELSD, adapted from previous publications [10,13,14,17,28,29]. The methods are described in detail in the Supplementary Materials Section S2. In short, FT-IR spectra were recorded in aqueous solution at a wavelength from 4000 cm^−1^ to 600 cm^−1^ (PerkinElmer Inc., Waltham, MA, USA). The absorbance maxima in the TFA^−^ absorbance range of 1210 to 1190 cm^−1^ were determined using Python (3.10.10, https://docs.python.org/3/reference (accessed 22 April 2024)). The ^19^F-NMR (Bruker Avance III 400 MHz NMR spectrometer, Bremen, Germany) measurements were performed in 10% deuterium oxide (D_2_O; >99.9 atom% D, Apollo Scientific, Bredbury, United Kingdom) in H_2_O (nanopure water, in-house water purification system ELGA Purelab, Villmergen, Switzerland) and the signal areas were determined via auto-integration. The HPLC-ELSD (VWR ELITE Lachrome Series, Dietikon, Switzerland) method was used under isocratic conditions with a mobile phase consisting of 40% 20 mM ammonium formate (≥99%, Sigma Aldrich, Buchs, Switzerland) in H_2_O (pH 4.0) and 60% acetonitrile (ACN; HPLC gradient grade, ≥99.9%, Sigma Aldrich, Buchs, Switzerland). Separation was achieved on an Acclaim Trinity P1 column (3 mm × 100 mm, Thermo Fisher Scientific, Sunnyvale, CA, USA) allowing for the simultaneous detection of TFA^−^, Cl^−^, and Na^+^. Retention times were determined by single injection of analytes and quantification performed via peak area. All analytical methods were validated according to the ICH guidelines [30,31]. For each analysis method, calibrators with concentrations in the corresponding quantitative range for TFA^−^ (additionally, Na^+^ and Cl^−^ for HPLC-ELSD) were prepared and aliquoted for each validation day (4 days for FT-IR and ^19^F-NMR, 8 days for HPLC-ELSD). Quality control (QC) samples (QC low, med, and high) were prepared independently. All aliquots were stored at 5 °C. Over a period of 7 (FT-IR and ^19^F-NMR) or 21 days (HPLC-ELSD), daily regression curves were determined using the generalized least squares regression model. For the ^19^F-NMR and FT-IR method, a linear model was used, and for the HPLC-ELSD method a polynomial model [10,41]. Weightings of x, 1/x, and 1/x^2^ were considered for the calibration curves. The accuracy was determined by comparing the established concentration of the QC sample concentration and the theoretical concentration. Bias and intra- and inter-day precision (RSD_R_ and RSD_T_) were calculated according to Peters et al. [30]. Acceptance criteria were set to <15% for QC high and medium and <20% for QC low samples. The limit of detection (LoD) and limit of quantification (LoQ) for the FT-IR and ^19^F-NMR methods were calculated according following ICH guidelines based on the standard deviation of the response and slope. As a quadratic regression curve was determined for the HPLC-ELSD method, LoD and LoQ were determined using the signal-to-noise approach. To assess the stability of the calibration curve, the concentration of the QCs for each day was recalculated with the calibration curve established on the first day and the bias was determined by comparison with the theoretical value calculated. 

### 3.2. Peptide Synthesis, Purification, and Characterization

The peptides listed in Table 2 were synthesized by solid phase peptide synthesis using the Fmoc protection strategy and subsequently purified. The identity of the peptides was confirmed by LC-MS analysis and the purity of the peptides determined by LC-UV. The detailed procedures can be found in the Supplementary Materials Section S1. Synthesized peptides included AT 1–4 as well as the CPPs AntP, Pep1, and PVEC [32,33,34,35].

### 3.3. Monitoring of Counterion Exchange

For the counterion exchange of TFA^−^ with Cl^−^, the peptide salt was dissolved in a diluted aqueous HCl solution at a concentration of 1 mg/ml. The solutions were frozen at −80 °C freezer and lyophilized three times. To determine the optimal concentration of HCl needed for counterion exchange, the experiments were conducted with 0, 2, 5, 10, and 100 mM HCl (diluted hydrochloric acid 37% EMSURE Supelco, Merck, Darmstadt, Germany) for AT 1. To evaluate the influence of the peptide sequence, counterion exchange was also performed for AT 2, AT 3, and AT 4 with the optimally determined salt concentration of 10 mM. Additionally, exchange with 0 mM HCl was performed as a negative control. For all peptide samples, the content of counterions was measured by FT-IR, ^19^F-NMR, and HPLC-ELSD before the exchange as well as after each lyophilization cycle (Ex 0, Ex 1, Ex 2, and Ex 3). FT-IR measurements were conducted with 100 µL of a 4 mg/mL peptide salt solution in H_2_O as singlets. ^19^F-NMR experiments were conducted with a 2 mg/mL peptide salt solution in 10% D_2_O in triplicate. HPLC-ELSD samples containing 0.75 mg/mL peptide salt in H_2_O were measured in triplicate with an injection volume of 5 and 20 µL. The purity of the samples was monitored during the counterion exchange with a 0.25 mg/mL peptide salt solution according to the purity method described below. For the CPPs, counterion exchange was performed with three cycles using 10 mM HCl. For data analysis and visualization, GraphPad Prism (Version 10.2.0, GraphPad Software, Boston, MA, USA) was used.

### 3.4. Liposomal Fluorescence Assay for Permeation Studies

To investigate the influence of the counterions on the membrane permeability of peptides, a liposomal fluorescence assay was performed using a previously described method adapted to a plate reader [37,38]. Liposomes were prepared by thin-film hydration and extrusion as described in the following. 2-Oleoyl-1-palmitoyl-*sn*-glycero-3-phosphatidylcholine (POPC; Apollo Scientific, Bredbury, UK) was dissolved in methanol (MeOH; Emsure Supelco, Ph. Eur., gradient grade, Merck, Darmstadt, Germany) with a concentration of 10 mg/mL. The MeOH was subsequently evaporated in vacuum at 37 °C. The lipid film was hydrated with the liposomal inner phase, containing 10 mM pyranine (HPTS, 8-Hydroxypyrene-1,3,6-trisulfonic acid; Thermo Fisher Scientific, Heysham, UK) and 200 mM NaCl (≥99.5%, Sigma Aldrich, Buchs, Switzerland) resulting in a 10 mg/mL liposomal solution. The liposomal solution underwent 10 freeze (dry ice bath with isopropanol) and thaw (37 °C) cycles followed by extrusion with a 10 mL thermobarrel extruder (LipexTM, Northern Lipids, Burnaby, Canada). Extrusion was performed ten times at room temperature through a membrane with a 200 nm pore size (Whatman Nuclepore Track Etch membrane, Sigma Aldrich, Buchs, Switzerland). Liposomes were stored at 4 °C and used within three days. The outer phase of the liposomes was exchanged with a buffer containing 20 mM 2-(N-morpholino) ethanesulfonic acid (MES, Apollo Scientific, Bredbury, UK) and 180 mM NaCl adjusted to pH 6.0 using a desalting column (Sephadex G-25 resin PD-10 desalting column, GE Healthcare, Chicago, CA, USA) before each measurement series. Exchanged liposomes were equilibrated in the outer phase to allow pH exchange for at least 1 h and were used within 12 h. The average hydrodynamic diameter of the liposomes and the size distribution were determined by dynamic light scattering (Zetasizer 3000 HAS, Malvern Instruments, Malvern, UK) before and after each experimental series.

Permeation kinetics were measured on an Infinite 200 Pro plate reader (Tecan, Männedorf, Switzerland) in top mode with black round-bottom 96 well plates (Greiner Bio-One, Kremsmünster, Austria) at 28.5 ± 0.5 °C. Excitation and emission wavelengths were set according to the maximum values described for HPTS, corresponding to 452 nm for excitation and 512 nm for emission [55,56]. Peptide samples were prepared as 1 mM stock solutions in dimethyl sulfoxide (DMSO; ≥99.5%, Sigma Aldrich, Buchs, Switzerland) and diluted to 50 µM in the outer phase buffer. Permeation kinetics were determined for all synthetic peptides as TFA and Cl salt. HCl and TFA (5 µM) were used as controls in the absence of peptide. Liposomes were diluted to reach a fluorescent signal in the range of 4000 to 5000 arbitrary units corresponding to an approximately 1:75 dilution in the outer phase buffer. In total, 10 µL of the 50 µM peptide solution was pipetted into 100 µL of diluted liposomes, and fluorescence was acquired for 180 s with a gain of 100. Each sample was measured in triplicate. 

For data evaluation, measurement series were aligned to common timepoints by interpolation and averaged. The measured fluorescence kinetics were fitted with the biexponential function Equation (1).

(1) $$F=Ae^{-k_{a}t}+Be^{-k_{b}t}$$

*F* describes the measured fluorescence signal over the exposure time *t*, dependent on the rate constants *k_a_* (s^−1^) and *k_b_* (s^−1^) and the weighting factors A and B. The apparent permeability coefficient *Perm_app_* (cm/s) was calculated with Equation (2) resulting from the rate constant *k_a_* of the fastest phase and the average liposome diameter *d*.

(2) $${Perm}_{app}=k_{a}\frac{d}{6}$$

Data evaluation, curve fitting, and visualization were conducted with Python (3.10.10, https://docs.python.org/3/reference (accessed 22 April 2024)), NumPy (1.23.5) [57], Pandas (2.2.3, Available online: https://pandas.pydata.org/ (accessed on 22 January 2024)), Scipy (1.15.1, Available online: https://scipy.org/ (accessed on 23 August 2024)), and Matplotlib (3.7.1, Available online: https://matplotlib.org/ (accessed on 23 August 2024).) [57,58,59,60,61]. The full code used for evaluation and visualization can be found in Supplementary Materials Section S4.

## 4. Conclusions

Three analytical techniques—FT-IR, ^19^F-NMR, and mixed-mode HPLC-ELSD—were successfully adapted and validated for the detection and quantification of TFA^−^ in synthetic peptides. The developed techniques were used to conduct an in-depth investigation on commonly used counterion exchange protocols, with the aim of reaching a consensus on the HCl concentration and number of exchange cycles. A concentration of 10 mM HCl was identified as the optimal exchange concentration, effectively reducing the TFA^−^ content below the LoQ of all techniques after a single exchange cycle. A consistent excess of TFA^−^ relative to the theoretical amount was identified directly after purification, indicating the presence of unbound TFA^−^ which could be removed by repeating lyophilization cycles with water (0 mM HCl). Furthermore, a strong correspondence was found between the number of positively charged residues on the peptides and the amount of associated counterions, supporting our hypothesis that counterions such as TFA^−^ and Cl^−^ interact in a stoichiometric manner with positive charges on the peptide. To evaluate the functional implications of TFA^−^ and Cl^−^ as counterions on the membrane permeation and penetration of peptides, we used a liposomal fluorescence assay. While non-permeating peptides (AT 1–4) showed no substantial counterion-related differences, cell-penetrating peptides (CPPs) displayed variable permeation kinetics depending on the counterion, suggesting that counterions can modulate passive permeation. Based on our observations this could be due to the potential formation neutral peptide-anion complexes. Future work might include 2D-NMR or peptide crystallography to investigate the precise binding location and interaction of counterions with peptides. Furthermore, focus will be set on acetate as an alternative counterion in peptide formulation, Overall, our study provides a comprehensive overview on how to detect and exchange TFA^−^ as a counterion in synthetic peptides. This emphasizes that counterions are not passive components and might significantly influence the physicochemical and functional properties of peptides. The salt choice should therefore be explicitly reported and considered in peptide-based studies.

## Figures and Tables

**Table 1:** Key characteristics of the three validated methods including specific characteristic values such as bias, intra-day, and inter-day deviations determined during the validation.

	^19^F-NMR	HPLC-ELSD	FT-IR
Detected analytes	TFA^−^	Na^+^, Cl^−^, TFA^−^	TFA^−^
Analyte signal	Signal area at 75.4 ppm	Peak area at retention time 4.0 min (Na^+^), 5.4 min (TFA^−^), 6.6 min (Cl^−^)	Maximum absorbance 1190–1210 cm^−1^
Measurement time	4 min	10 min	1 min
Sample solvent	10% D_2_O/H_2_O	Water	Water
Automation with autosampler	Yes	Yes	No
Sample recovery	Possible	Destructive	Possible
Calibration model	Linear	Quadratic	Linear
Weighting	1/x	x	x
TFA^−^ calibration range	50.7–2536.5 µg/mL	10.24–409.5 µg/mL	503–2514 µg/mL
LoD TFA^−^	6.82 µg/mL	1.52 µg/mL	253 µg/mL
LoQ TFA^−^	20.68 µg/mL	4.61 µg/mL	713 µg/mL
Sample interference	Unlikely	Sequence dependent	Sequence dependent
Bias QC low/med/high	9.5%/3.6%/0.2%	13.2%/5.7%/0.1%	15.9%/4.6%/3.7%
Intra-day deviation QC low/med/high	1.1%/2.1%/1.7%	3.9%/3.2%/2.5%	16.8%/9.7%/4.5%
Inter-day deviation QC low/med/high	2.8%/2.9%/2.0%	6.6%/8.6%/6.6%	19.5%/11.7%/10.0%

**Table 2:** Overview of synthesized peptide sequences with calculated molecular weight and expected charge at pH < 3. Expected positively charged positions are indicated in bold in the sequence. The molecular weight (MW) and weight percentage of the counterion were calculated, assuming each positively charged position is stoichiometrically paired by a counterion. MWs were calculated with PICKAPEP [43]. Isoelectric points (pI) were predicted using pIChemist [44].

Peptide	Sequence *	pI	Charge at pH < 3	MW (Da)	MW as TFA Salt (Da)	Weight Percentage TFA^−^ (%)	MW as Cl Salt (Da)	Weight Percentage Cl^−^ (%)
AT 1	**H_2_N**-D**R**VYI**H**PF**H**L-COOH	7.44 ± 0.93	+4	1296.50	1752.58	26.0	1438.30	9.9
AT 2	**H_2_N**-D**R**VYI**H**PF-COOH	7.28 ± 0.98	+3	1046.20	1388.26	24.6	1152.55	9.2
AT 3	**H_2_N**-**R**VYI**H**PF-COOH	9.05 ± 1.42	+3	931.11	1273.17	26.9	1037.46	10.3
AT 4	**H_2_N**-VYI**H**PF-COOH	7.26 ± 1.05	+2	774.92	1002.96	22.7	845.82	8.4
AntP	**H_2_N**-**R**QI**K**IWFQN**RR**M**K**W**KK**-CONH_2_	12.92 ± 0.82	+8	2245.79	3157.95	28.9	2529.41	11.2
Pep1	**H_2_N**-**K**ETWWETWWTEWSQP**KKKRK**V-COOH	9.90 ± 2.02	+7	2848.27	3646.41	21.9	3096.44	8.0
pVEC	**H_2_N**-LLIIL**RRR**I**RK**QA**H**A**H**S**K**-COOH	12.60 ± 0.90	+9	2096.57	3235.91	35.2	2528.81	17.1

* bold: basic functional groups, underlined: acidic functional groups.

## Data Availability

Exemplary data for all experiments are available in the publication or Supporting Information. Raw data is available from the authors upon request.

## Supplementary Materials

The following supporting information can be downloaded at https://www.mdpi.com/article/10.3390/ph18081163/s1, Section S1: Peptide synthesis, purification and characterization; Section S2: Method Development and Validation; Section S3: Salt Exchange; Section S4: Liposomal Assay; Figure S1: Peptide purity after synthesis and purification determined by HPLC-UV; Figure S2: RP-LC chromatograms and mass spectra of AT1–4; Figure S3: RP-LC chromatograms and mass spectra of CPPs; Figure S4: Calibration data day 1 19F-NMR; Figure S5: QC samples day 1 19F-NMR; Figure S6: Calibration data day 1 HPLC-ELSD; Figure S7: QC samples day 1 HPLC-ELSD; Figure S8: Calibration data FT-IR; Figure S9: 19F-NMR spectra of AT1 with 0 mM HCl exchange; Figure S10: 19F-NMR spectra of AT1 with 2 mM HCl exchange; Figure S11: 19F-NMR spectra of AT1 with 5 mM HCl exchange; Figure S12: 19F-NMR spectra of AT1 with 10 mM HCl exchange; Figure S13: 19F-NMR spectra of AT1 with 100 mM HCl exchange; Figure S14: 19F-NMR spectra of AT2 with 0 mM HCl exchange; Figure S15: 19F-NMR spectra of AT2 with 10 mM HCl exchange; Figure S16: 19F-NMR spectra of AT3 with 0 mM HCl exchange; Figure S17: 19F-NMR spectra of AT3 with 10 mM HCl exchange; Figure S18: 19F-NMR spectra of AT4 with 0 mM HCl exchange; Figure S19: 19F-NMR spectra of AT4 with 10 mM HCl exchange; Figure S20: 19F-NMR spectra of CPPs before and after three counterion exchange cycles with 10 mM HCl; Figure S21: HPLC-ELSD chromatograms of AT1 for 0 mM HCl exchange; Figure S22: HPLC-ELSD chromatograms of AT1 for 2 mM HCl exchange; Figure S23: HPLC-ELSD chromatograms of AT1 for 5 mM HCl exchange; Figure S24: HPLC-ELSD chromatograms of AT1 for 10 mM HCl exchange; Figure S25: HPLC-ELSD chromatograms of AT1 for 100 mM HCl exchange; Figure S26: HPLC-ELSD chromatograms of AT2 for 10 mM HCl exchange; Figure S27: HPLC-ELSD chromatograms of AT3 for 10 mM HCl exchange; Figure S28: HPLC-ELSD chromatograms of AT4 for 10 mM HCl exchange; Figure S29: FT-IR data of salt exchange of AT1 with different concentrations of HCl; Figure S30: FT-IR data of salt exchange of AT1–4 with 10 mM HCl; Figure S31: Purity of AT1 over three counterion exchanges with 0 mM HCl; Figure S32: Purity of AT1 over three counterion exchanges with 2 mM HCl; Figure S33: Purity of AT1 over three counterion exchanges with 5 mM HCl; Figure S34: Purity of AT1 over three counterion exchanges with 10 mM HCl; Figure S35: Purity of AT1 over three counterion exchanges with 100 mM HCl; Figure S36: Purity of AT2 over three counterion exchanges with 10 mM HCl; Figure S37: Purity of AT3 over three counterion exchanges with 10 mM HCl; Figure S38: Purity of AT4 over three counterion exchanges with 10 mM HCl; Figure S39: Purity of CPPs before and after three counterion exchanges with 10 mM HCl; Figure S40: Cl and Na per mg peptide salt determined by HPLC-ELSD for AT1–4; Figure S41: Comparison of calculations of number of TFA per peptide determined by 19F-NMR, FT-IR or HPLC-ELSD for AT1 exchanged with 0 mM HCl; Figure S42: Number of TFA per peptide per exchange cycle for AT1 at different concentrations of HCl determined by HPLC-ELSD; Figure S43: Number of counterions per exchange cycle for AT1–4 determined by HPLC-ELSD; Figure S44: Content of counterion per mg peptide for AT1-4 at different concentrations of HCl over 3 exchange cycles; Figure S45: Content of TFA per mg peptide salt and number of TFA per peptide for AntP, Pep1 and pVEC; Figure S46: General mechanism of the liposomal assay for a basic compound; Figure S47: Permeation kinetics of AT2 and AT3 as TFA and Cl salt; Table S1: Summary of method validation; Table S2: Peptide purity determined by HPLC-UV before and after HCl exchange; Table S3: TFA determination by IR; Table S4: TFA determination by NMR; Table S5: TFA, Cl and Na determination by HPLC-ELSD; Table S6: Calculations number of counterions per peptide for IR, NMR and HPLC-ELSD; Table S7: Size of liposomes used in liposomal assay; Table S8: pH measurements of AT1–4 peptide salt in aq. solution after exchange and lyophilization; Table S9: Parameters of biexponential curve fitting for all liposomal permeation assay experiments.

## References

[1] Wang L., Wang N., Zhang W., Cheng X., Yan Z., Shao G., Wang X., Wang R., Fu C. (2022). Therapeutic peptides: Current applications and future directions. Signal Transduct. Target. Ther..

[2] Lau J.L., Dunn M.K. (2018). Therapeutic peptides: Historical perspectives, current development trends, and future directions. Bioorganic Med. Chem..

[3] Pereira A.J., de Campos L.J., Xing H., Conda-Sheridan M. (2024). Peptide-based therapeutics: Challenges and solutions. Med. Chem. Res..

[4] Erckes V., Steuer C. (2022). A story of peptides, lipophilicity and chromatography—Back and forth in time. RSC Med. Chem..

[5] Merrifield R.B. (1963). Solid phase peptide synthesis. I. The synthesis of a tetrapeptide. J. Am. Chem. Soc..

[6] Chan W., White P. (1999). Fmoc Solid Phase Peptide Synthesis: A Practical Approach.

[7] Carpino L.A., Han G.Y. (1970). 9-Fluorenylmethoxycarbonyl function, a new base-sensitive amino-protecting group. J. Am. Chem. Soc..

[8] Shibue M., Mant C.T., Hodges R.S. (2005). Effect of anionic ion-pairing reagent hydrophobicity on selectivity of peptide separations by reversed-phase liquid chromatography. J. Chromatogr. A.

[9] Sikora K., Jaskiewicz M., Neubauer D., Migon D., Kamysz W. (2020). The Role of Counter-Ions in Peptides—An Overview. Pharmaceuticals.

[10] Streuli A., Coxon C.R., Steuer C. (2021). Simultaneous Quantification of Commonly Used Counter Ions in Peptides and Active Pharmaceutical Ingredients by Mixed Mode Chromatography and Evaporative Light Scattering Detection. J. Pharm. Sci..

[11] Allenspach M.D., Fuchs J.A., Doriot N., Hiss J.A., Schneider G., Steuer C. (2018). Quantification of hydrolyzed peptides and proteins by amino acid fluorescence. J. Pept. Sci..

[12] Gaussier H., Morency H., Lavoie M.C., Subirade M. (2002). Replacement of trifluoroacetic acid with HCl in the hydrophobic purification steps of pediocin PA-1: A structural effect. Appl. Environ. Microbiol..

[13] Andrushchenko V.V., Vogel H.J., Prenner E.J. (2007). Optimization of the hydrochloric acid concentration used for trifluoroacetate removal from synthetic peptides. J. Pept. Sci..

[14] Roux S., Zekri E., Rousseau B., Paternostre M., Cintrat J.C., Fay N. (2008). Elimination and exchange of trifluoroacetate counter-ion from cationic peptides: A critical evaluation of different approaches. J. Pept. Sci..

[15] Cornish J., Callon K., Lin C.-X., Xiao C., Mulvey T., Cooper G., Reid I. (1999). Trifluoroacetate, a contaminant in purified proteins, inhibits proliferation of osteoblasts and chondrocytes. Am. J. Physiol.Endocrinol. Metab..

[16] Sikora K., Jaśkiewicz M., Neubauer D., Bauer M., Bartoszewska S., Barańska-Rybak W., Kamysz W. (2018). Counter-ion effect on antistaphylococcal activity and cytotoxicity of selected antimicrobial peptides. Amino Acids.

[17] López-Sánchez A.G., Rodríguez-Mejía K.G., Cuero-Amu K.J., Ardila-Chantré N., Reyes-Calderón J.E., González-López N.M., Huertas-Ortiz K.A., Fierro-Medina R., Rivera-Monroy Z.J., García-Castañeda J.E. (2024). A New Methodology for Synthetic Peptides Purification and Counterion Exchange in One Step Using Solid-Phase Extraction Chromatography. Processes.

[18] Ardino C., Sannio F., Pasero C., Botta L., Dreassi E., Docquier J.-D., D’Agostino I. (2023). The impact of counterions in biological activity: Case study of antibacterial alkylguanidino ureas. Mol. Divers..

[19] Pini A., Lozzi L., Bernini A., Brunetti J., Falciani C., Scali S., Bindi S., Di Maggio T., Rossolini G.M., Niccolai N. (2012). Efficacy and toxicity of the antimicrobial peptide M33 produced with different counter-ions. Amino Acids.

[20] Ma T.G., Ling Y.H., McClure G.D., Tseng M.T. (1990). Effects of trifluoroacetic acid, a halothane metabolite, on C6 glioma cells. J. Toxicol. Environ. Health.

[21] Dekant W., Dekant R. (2023). Mammalian toxicity of trifluoroacetate and assessment of human health risks due to environmental exposures. Arch. Toxicol..

[22] Barlow N., Chalmers D.K., Williams-Noonan B.J., Thompson P.E., Norton R.S. (2020). Improving Membrane Permeation in the Beyond Rule-of-Five Space by Using Prodrugs to Mask Hydrogen Bond Donors. ACS Chem. Biol..

[23] Calabretta L.O., Yang J., Raines R.T. (2023). N(α) -Methylation of arginine: Implications for cell-penetrating peptides. J. Pept. Sci..

[24] Alex A., Millan D.S., Perez M., Wakenhut F., Whitlock G.A. (2011). Intramolecular hydrogen bonding to improve membrane permeability and absorption in beyond rule of five chemical space. MedChemComm.

[25] Kramer S.D., Aschmann H.E., Hatibovic M., Hermann K.F., Neuhaus C.S., Brunner C., Belli S. (2016). When barriers ignore the “rule-of-five”. Adv. Drug Deliv. Rev..

[26] Liu X., Testa B., Fahr A. (2011). Lipophilicity and its relationship with passive drug permeation. Pharm. Res..

[27] Sikora K., Neubauer D., Jaskiewicz M., Kamysz W. (2018). Citropin 1.1 Trifluoroacetate to Chloride Counter-Ion Exchange in HCl-Saturated Organic Solutions: An Alternative Approach. Int. J. Pept. Res. Ther..

[28] Kaiser E., Rohrer J. (2004). Determination of residual trifluoroacetate in protein purification buffers and peptide preparations by ion chromatography. J. Chromatogr. A.

[29] Little M.J., Aubry N., Beaudoin M.-E., Goudreau N., LaPlante S.R. (2007). Quantifying trifluoroacetic acid as a counterion in drug discovery by 19F NMR and capillary electrophoresis. J. Pharm. Biomed. Anal..

[30] Peters F.T., Drummer O.H., Musshoff F. (2007). Validation of new methods. Forensic Sci. Int..

[31] ICH Harmonised Guideline (2022). Validation of Analytical Procedures Q2 (R2).

[32] Chaloin L., Vidal P., Lory P., Mery J., Lautredou N., Divita G., Heitz F. (1998). Design of carrier peptide-oligonucleotide conjugates with rapid membrane translocation and nuclear localization properties. Biochem. Biophys. Res. Commun..

[33] Morris M.C., Depollier J., Mery J., Heitz F., Divita G. (2001). A peptide carrier for the delivery of biologically active proteins into mammalian cells. Nat. Biotechnol..

[34] Elmquist A., Lindgren M., Bartfai T., Langel U. (2001). VE-cadherin-derived cell-penetrating peptide, pVEC, with carrier functions. Exp. Cell Res..

[35] Derossi D., Joliot A.H., Chassaing G., Prochiantz A. (1994). The third helix of the Antennapedia homeodomain translocates through biological membranes. J. Biol. Chem..

[36] Copolovici D.M., Langel K., Eriste E., Langel U. (2014). Cell-penetrating peptides: Design, synthesis, and applications. ACS Nano.

[37] Hermann K.F., Neuhaus C.S., Micallef V., Wagner B., Hatibovic M., Aschmann H.E., Paech F., Alvarez-Sanchez R., Kramer S.D., Belli S. (2017). Kinetics of lipid bilayer permeation of a series of ionisable drugs and their correlation with human transporter-independent intestinal permeability. Eur. J. Pharm. Sci..

[38] Eyer K., Paech F., Schuler F., Kuhn P., Kissner R., Belli S., Dittrich P.S., Kramer S.D. (2014). A liposomal fluorescence assay to study permeation kinetics of drug-like weak bases across the lipid bilayer. J. Control. Release.

[39] Valenti L.E., Paci M.B., De Pauli C.P., Giacomelli C.E. (2011). Infrared study of trifluoroacetic acid unpurified synthetic peptides in aqueous solution: Trifluoroacetic acid removal and band assignment. Anal. Biochem..

[40] Clement A., Yong D., Brechet C. (1992). Simultaneous identification of sugars by HPLC using evaporative light scattering detection (ELSD) and refractive index detection (RI). Application to plant tissues. J. Liq. Chromatogr. Relat. Technol..

[41] Montesano D., Cossignani L., Giua L., Urbani E., Simonetti M.S., Blasi F. (2016). A Simple HPLC-ELSD Method for Sugar Analysis in Goji Berry. J. Chem..

[42] Camdzic D., Dickman R.A., Joyce A.S., Wallace J.S., Ferguson P.L., Aga D.S. (2023). Quantitation of total PFAS including trifluoroacetic acid with fluorine nuclear magnetic resonance spectroscopy. Anal. Chem..

[43] Erckes V., Hilleke M., Isert C., Steuer C. (2024). PICKAPEP: An application for parameter calculation and visualization of cyclized and modified peptidomimetics. J. Pept. Sci..

[44] Frolov A.I., Chankeshwara S.V., Abdulkarim Z., Ghiandoni G.M. (2023). pIChemiSt—Free Tool for the Calculation of Isoelectric Points of Modified Peptides. J. Chem. Inf. Model..

[45] Nugrahadi P.P., Hinrichs W.L.J., Frijlink H.W., Schöneich C., Avanti C. (2023). Designing Formulation Strategies for Enhanced Stability of Therapeutic Peptides in Aqueous Solutions: A Review. Pharmaceutics.

[46] Hu Y., Chen S., Ye S., Wei S., Chu B., Wang R., Li H., Zhang T. (2023). The role of trifluoroacetic acid in new particle formation from methanesulfonic acid-methylamine. Atmos. Environ..

[47] Madani F., Lindberg S., Langel Ü., Futaki S., Gräslund A. (2011). Mechanisms of Cellular Uptake of Cell-Penetrating Peptides. J. Biophys..

[48] Jiao C.-Y., Delaroche D., Burlina F., Alves I.D., Chassaing G., Sagan S. (2009). Translocation and Endocytosis for Cell-penetrating Peptide Internalization. J. Biol. Chem..

[49] Eggimann G.A., Buschor S., Darbre T., Reymond J.-L. (2013). Convergent synthesis and cellular uptake of multivalent cell penetrating peptides derived from Tat, Antp, pVEC, TP10 and SAP. Org. Biomol. Chem..

[50] Troeira Henriques S., Alexandre Q., Bagatolli L.A., Fabrice H., Castanho M.A.R.B. (2007). Energy-independent translocation of cell-penetrating peptides occurs without formation of pores. A biophysical study with pep-1. Mol. Membr. Biol..

[51] Elmquist A., Hansen M., Langel Ü. (2006). Structure–activity relationship study of the cell-penetrating peptide pVEC. Biochim. Biophys. Acta BBA Biomembr..

[52] Guterstam P., Madani F., Hirose H., Takeuchi T., Futaki S., El Andaloussi S., Gräslund A., Langel Ü. (2009). Elucidating cell-penetrating peptide mechanisms of action for membrane interaction, cellular uptake, and translocation utilizing the hydrophobic counter-anion pyrenebutyrate. Biochim. Biophys. Acta BBA Biomembr..

[53] Forrester S.J., Booz G.W., Sigmund C.D., Coffman T.M., Kawai T., Rizzo V., Scalia R., Eguchi S. (2018). Angiotensin II Signal Transduction: An Update on Mechanisms of Physiology and Pathophysiology. Physiol. Rev..

[54] Ristroph K.D., Prud’homme R.K. (2019). Hydrophobic ion pairing: Encapsulating small molecules, peptides, and proteins into nanocarriers. Nanoscale Adv..

[55] Nandi R., Amdursky N. (2022). The Dual Use of the Pyranine (HPTS) Fluorescent Probe: A Ground-State pH Indicator and an Excited-State Proton Transfer Probe. Acc. Chem. Res..

[56] Launay M., Tripier M., Guizerix J., Viriot M.L., Andre J.C. (1980). Pyranine used as a fluorescent tracer in hydrology: pH effects in determination of its concentration. J. Hydrol..

[57] Harris C.R., Millman K.J., van der Walt S.J., Gommers R., Virtanen P., Cournapeau D., Wieser E., Taylor J., Berg S., Smith N.J. (2020). Array programming with NumPy. Nature.

[58] The Python Language Reference. https://docs.python.org/3/reference/.

[59] Pandas User Guide. https://pandas.pydata.org/.

[60] SciPy. https://scipy.org/.

[61] Matplotlib. https://matplotlib.org/.

