# Supplementary material for: Towards a Consensus for the Analysis and Exchange of TFA as a Counterion in Synthetic Peptides and Its Influence on Membrane Permeation
Source: Pharmaceuticals (Basel). 2025 Aug 5;18(8):1163. doi: 10.3390/ph18081163 (PMC12389442; doi:10.3390/ph18081163)
Supplement: Supplementary file 1 [file pharmaceuticals-18-01163-s001.zip › pharmaceuticals-3787541-supplementary.pdf]

Supporting Information

Towards a Consensus for the Analysis and Exchange of TFA as a Counterion in Synthetic Peptides and Its Influence on Membrane Permeation

Vanessa Erckes [1,†], Alessandro Streuli [1,†], Laura Chamera Rendueles [1], Stefanie Dorothea Krämer [2] and Christian Steuer [1,*]

[1] Pharmaceutical Analytics, Department of Chemistry and Applied Biosciences, ETH Zurich, 8093 Zurich, Switzerland; vanessa.erckes@pharma.ethz.ch (V.E.); alessandro.streuli@pharma.ethz.ch (A.S.)
[2] Biopharmacy, Department of Chemistry and Applied Biosciences, ETH Zurich, 8093 Zurich, Switzerland; stefanie.kraemer@pharma.ethz.ch
* Correspondence: christian.steuer@pharma.ethz.ch; Tel.:+41-44-633-74-19
† These authors contributed equally to this work.

ORCID

VE: https://orcid.org/0000-0002-9650-4160

AS: https://orcid.org/0000-0002-0025-8023

SK: https://orcid.org/0000-0002-0426-4340

CS: https://orcid.org/0000-0002-6102-3367

SECTION S1: PEPTIDE SYNTHESIS, PURIFICATION AND CHARACTERIZATION 4
SECTION S2: METHOD DEVELOPMENT AND VALIDATION 8
SECTION S2.1: ^{19}F-NMR 8
SECTION S2.2: HPLC-ELSD 11
SECTION S2.3: FT-IR 14
SECTION S2.4: SUMMARY VALIDATION RESULTS 18

SECTION S3: SALT EXCHANGE 20
SECTION S3.1: ^{19}F-NMR 20
SECTION S3.2: HPLC-ELSD 28
SECTION S3.3: FT-IR 33
SECTION S3.4: PURITY 35
SECTION S3.5: ADDITIONAL FIGURES AND TABLES 40

SECTION S4: LIPOSOMAL ASSAY 54

FIGURE S1: PEPTIDE PURITY AFTER SYNTHESIS AND PURIFICATION DETERMINED BY HPLC-UV 5
FIGURE S2: RP-LC CHROMATOGRAMS AND MASS SPECTRA OF AT1-4 6

Figure S3:RP-LC chromatograms and mass spectra of CPPs 7
Figure S4: Calibration data day 1 ^{19}F-NMR 9
Figure S5: QC samples day 1 ^{19}F-NMR 10
Figure S6: Calibration data day 1 HPLC-ELSD 12
Figure S7: QC samples day 1 HPLC-ELSD 13
Figure S8: Calibration data FT-IR 15
Figure S9: ^{19}F-NMR spectra of AT1 with 0 mM HCl exchange 20
Figure S10: ^{19}F-NMR spectra of AT1 with 2 mM HCl exchange 21
Figure S11: ^{19}F-NMR spectra of AT1 with 5 mM HCl exchange 21
Figure S12: ^{19}F-NMR spectra of AT1 with 10 mM HCl exchange 22
Figure S13: ^{19}F-NMR spectra of AT1 with 100 mM HCl exchange 23
Figure S14: ^{19}F-NMR spectra of AT2 with 0 mM HCl exchange 23
Figure S15: ^{19}F-NMR spectra of AT2 with 10 mM HCl exchange 24
Figure S16: ^{19}F-NMR spectra of AT3 with 0 mM HCl exchange 24
Figure S17: ^{19}F-NMR spectra of AT3 with 10 mM HCl exchange 25
Figure S18: ^{19}F-NMR spectra of AT4 with 0 mM HCl exchange 26
Figure S19: ^{19}F-NMR spectra of AT4 with 10 mM HCl exchange 26
Figure S20: ^{19}F-NMR spectra of CPPs before and after three counterion exchange cycles with 10 mM HCl 27
Figure S21: HPLC-ELSD chromatograms of AT1 for 0 mM HCl exchange 28
Figure S22: HPLC-ELSD chromatograms of AT1 for 2 mM HCl exchange 29
Figure S23: HPLC-ELSD chromatograms of AT1 for 5 mM HCl exchange 29
Figure S24: HPLC-ELSD chromatograms of AT1 for 10 mM HCl exchange 30
Figure S25: HPLC-ELSD chromatograms of AT1 for 100 mM HCl exchange 30
Figure S26: HPLC-ELSD chromatograms of AT2 for 10 mM HCl exchange 31
Figure S27: HPLC-ELSD chromatograms of AT3 for 10 mM HCl exchange 31
Figure S28: HPLC-ELSD chromatograms of AT4 for 10 mM HCl exchange 32
Figure S29: FT-IR data of salt exchange of AT1 with different concentrations of HCl 33
Figure S30: FT-IR data of salt exchange of AT1-4 with 10 mM HCl 34
Figure S31: Purity of AT1 over three counterion exchanges with 0 mM HCl 35
Figure S32: Purity of AT1 over three counterion exchanges with 2 mM HCl 36
Figure S33: Purity of AT1 over three counterion exchanges with 5 mM HCl 36
Figure S34: Purity of AT1 over three counterion exchanges with 10 mM HCl 37
Figure S35: Purity of AT1 over three counterion exchanges with 100 mM HCl 37
Figure S36: Purity of AT2 over three counterion exchanges with 10 mM HCl 38
Figure S37: Purity of AT3 over three counterion exchanges with 10 mM HCl 38
Figure S38: Purity of AT4 over three counterion exchanges with 10 mM HCl 39
Figure S39: Purity of CPPs before and after three counterion exchanges with 10 mM HCl 39
Figure S40: Cl and Na per mg peptide salt determined by HPLC-ELSD for AT1-4 40
Figure S41: Comparison of calculations of number of TFA per peptide determined by 19F-NMR, FT-IR or HPLC-ELSD for AT1 exchanged with 0 mM HCl 40
Figure S42: Number of TFA per peptide per exchange cycle for AT1 at different concentrations of HCl determined by HPLC-ELSD 40
Figure S43: Number of counterions per exchange cycle for AT1-4 determined by HPLC-ELSD ... 41
Figure S44: Content of counterion per mg peptide for AT1-4 at different concentrations of HCl over 3 exchange cycles 41
Figure S45: Content of TFA per mg peptide salt and number of TFA per peptide for AntP, Pep1 and pVEC 41
Figure S46: General mechanism of the liposomal assay for a basic compound 54

FIGURE S47: PERMEATION KINETICS OF AT2 AND AT3 AS TFA AND CL SALT 54

TABLE S1: SUMMARY OF METHOD VALIDATION 18
TABLE S2: PEPTIDE PURITY DETERMINED BY HPLC-UV BEFORE AND AFTER HCL EXCHANGE 35
TABLE S3: TFA DETERMINATION BY IR 42
TABLE S4: TFA DETERMINATION BY NMR 44
TABLE S5: TFA, CL AND NA DETERMINATION BY HPLC-ELSD 46
TABLE S6: CALCULATIONS NUMBER OF COUNTERIONS PER PEPTIDE FOR IR, NMR AND HPLC-ELSD 50
TABLE S7: SIZE OF LIPOSOMES USED IN LIPOSOMAL ASSAY 54
TABLE S8: PH MEASUREMENTS OF AT1-4 PEPTIDE SALT IN AQ. SOLUTION AFTER EXCHANGE AND LYOPHILIZATION 55
TABLE S9: PARAMETERS OF BIEXPONENTIAL CURVE FITTING FOR ALL LIPOSOMAL PERMEATION ASSAY EXPERIMENTS 60

Section S1: **Peptide Synthesis, Purification and Characterization**

All peptides were synthesized by solid phase peptide synthesis on a PurePrep Chorus peptide synthesizer (Gyros Protein Technologies, Warren, NJ, USA) using the corresponding preloaded Wang resin. Synthesis was performed according to standard protocols using dimethylformamide (DMF; EMPLURA Supelco, Merck, Darmstadt, Germany) as solvent and swelling agent, pyrrolidine (99+%, Thermo Scientific,) in DMF (20% v/v; Sigma Aldrich, Buchs, Switzerland) as deprotection agent and a solution of 5 eq. O-(1H-6-Chlorobenzotriazole-1-yl)-1,1,3,3-tetramethyluronium hexafluorophosphate (HCTU; Gyros Proteins Technologies) and 10 eq. 2,6-dimethylmorpholine (NMM; 99+%, Thermo Scientific) as coupling agents with 5 eq. of Fmoc-protected amino acids (Protein Technologies, United Kingdoms). Peptides were cleaved and fully deprotected with 95% TFA (99.5%, Apollo Scientific), 2.5% TIS (Sigma Aldrich, Buchs, Switzerland), and 2.5% H_2O (v/v/v). Peptides were washed twice with ether (Emsure Supelco, Ph. Eur., Merck,) and purified by C18 reversed-phase (RP) flash chromatography (puriFlash XS520Plus, Interchim, Montluçon, France). Received fractions were lyophilized. Dried peptides were dissolved in H_2O with 0.1% formic acid (FA, 98.0-100%, Sigma Aldrich, Buchs, Switzerland) to a concentration of 0.1 mg/mL for further analysis.

Mass accuracy of peptides was confirmed by LC-MS analysis using a Waters Acquity™ UPLC system (Milford, MA, USA) coupled to an LTQ-XL linear ion trap equipped with a heated ESI II source (Thermo Scientific, San Jose (CA), United States). The mobile phase consisted of 0.1% FA in H_2O (eluent A) and 0.1% FA in acetonitrile (ACN; OPTIMA, LC-MS grade, Merck, Darmstadt, Germany, (eluent B). Gradient elution was performed on a Zorbax Eclipse Plus C18 RP column (2.1 x 50 mm, 1.8 µm; Agilent Technologies, Santa Clara, CA, USA) at room temperature. The autosampler temperature was set to 10 °C and the injection volume to 10 µL. The gradient was used as followed with a flow rate of 0.5 mL/min: 0-2 min at 5% B, 2-10 min to 70% B, 10-12 to 90% B, 12-15 min at 90% B, 15-15.5 min to 5% B,15.5-20 min reequilibration with 5% B. The ESI source was used non-heated with the sheath gas and the auxiliary gas set to 34 and 11 arbitrary units, respectively. The source voltage was 5.00 kV, the temperature of the ion transfer capillary 275 °C, the capillary voltage was 31 V and the tube lens voltage was 80 V. The MS analysis was conducted in positive mode with a full scan in the m/z range from 250 – 2000 and the scan rate set to normal. For data evaluation, Thermo Scientific Xcalibur software (Version 4.4.16.14, Thermo Scientific, San Jose (CA), United States) was used.

Purity control was performed using a VWR ELITE Lachrome Series LC with an UV detector (Dietikon, Switzerland). For chromatographic separation, an XBridge C18 RP column (3.5 µm, 4.6 x 150 mm, SN: USUXP01161, Waters Corporation, Millford, MA, USA) was used. The mobile phase for gradient elution consisted of 0.1% TFA in H_2O (eluent A) and 0.1% TFA in ACN (HPLC gradient grade, ≥ 99.9%, Sigma Aldrich) (eluent B). The used gradient with a flow rate of 1.0 mL/min was as followed: 0-5 min at 5% B, 5-50.0 min to 95% B, 50.0-55.0 min at 95% B, 55.0-56.0 min to 5% B, 56.0-65.0 min reequilibration with 5% B. The column oven was set to 25 °C and the injection volume to 10 µL. UV detection was performed at 214 nm wavelength with a sampling period of 200 ms and a response time of 1 s. For data evaluation, the OpenLab software (Version A. 04.08 - Agilent Technologies, Santa Clara, CA, USA) was used.

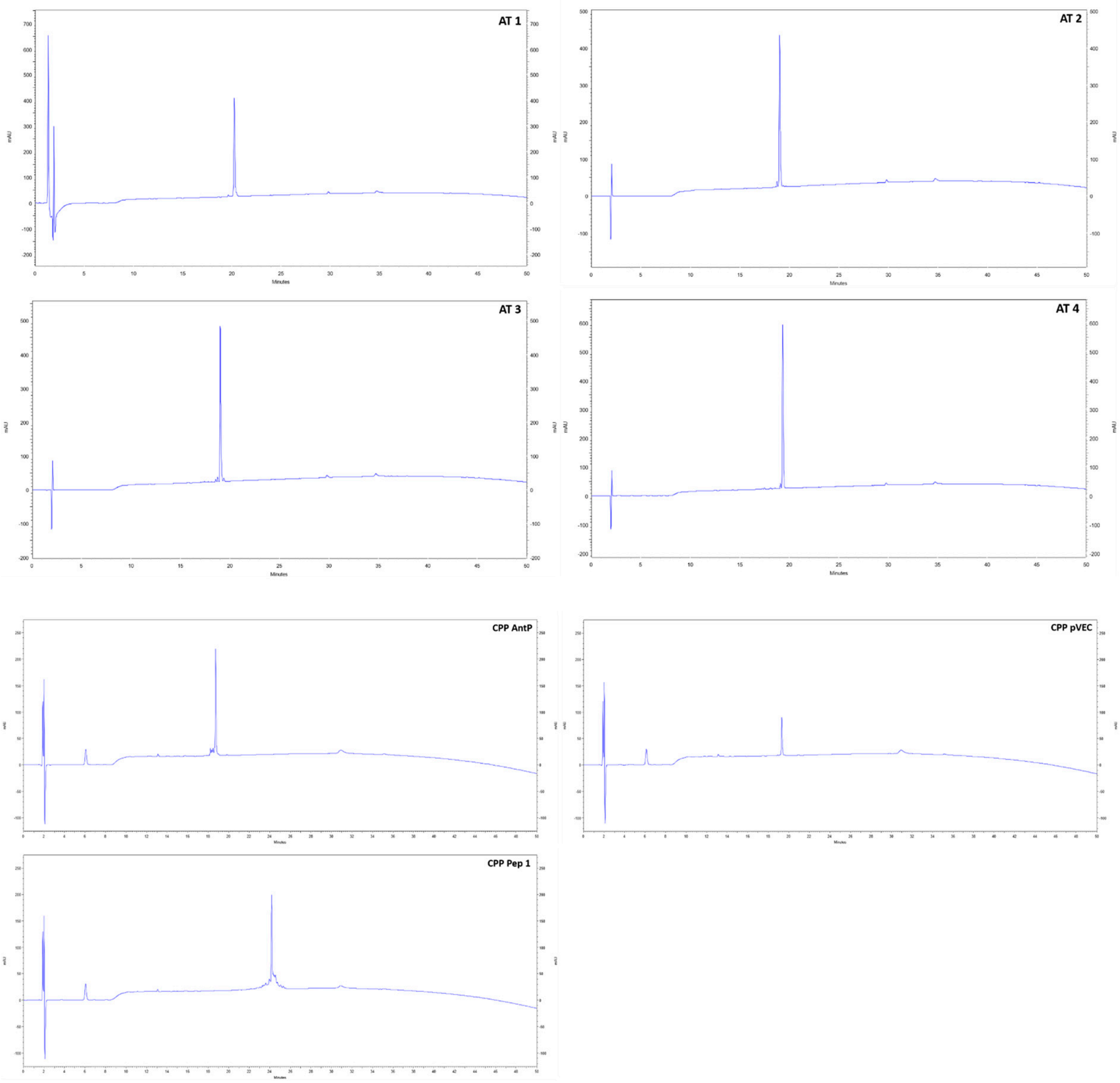

Figure S1: Peptide purity after synthesis and purification determined by HPLC-UV

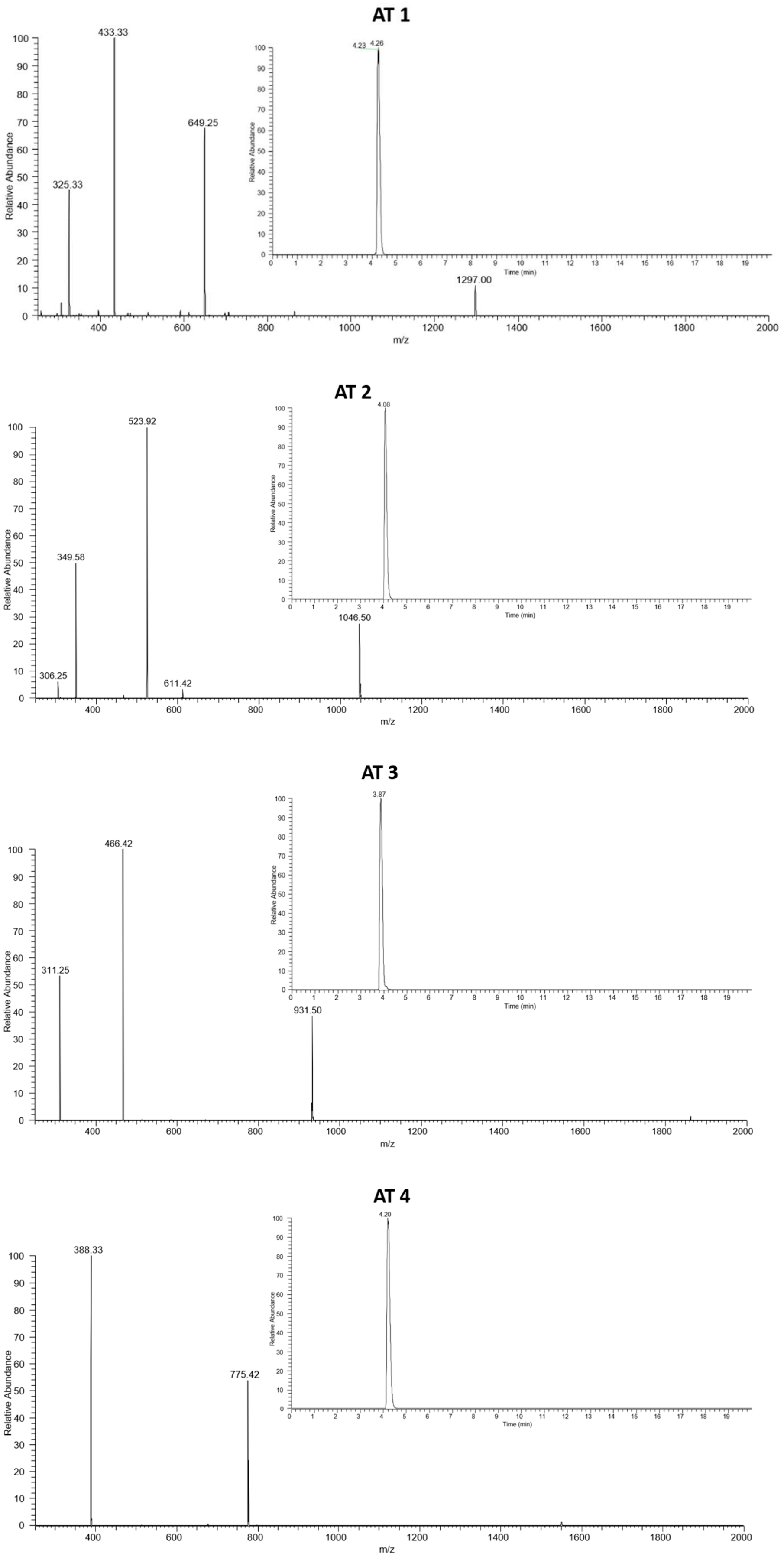

Figure S2: RP-LC chromatograms and mass spectra of AT1-4

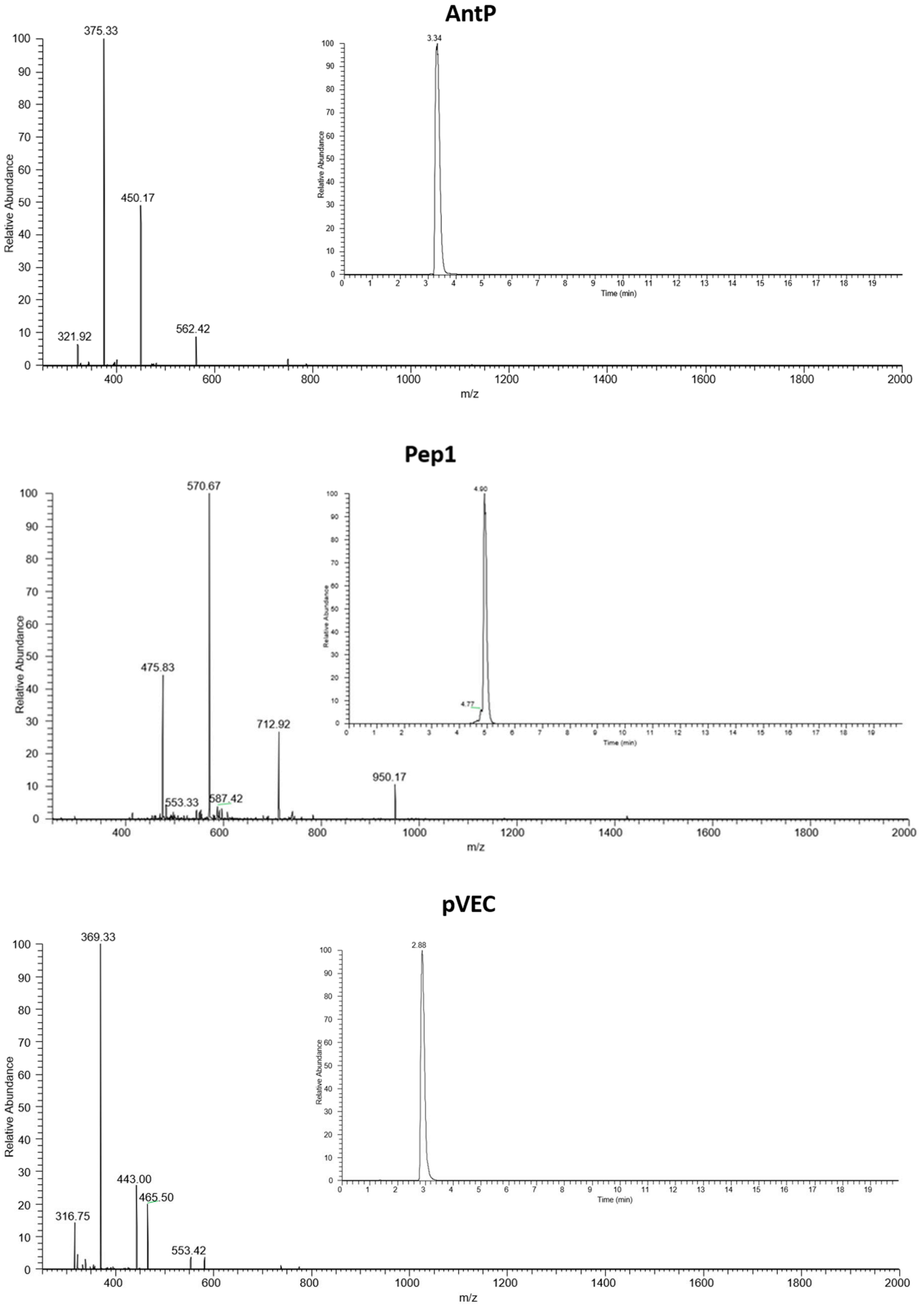

Figure S3:RP-LC chromatograms and mass spectra of CPPs

Section S2**: Method Development and Validation**

Section S2.1: ^{19}F-NMR

NMR experiments were conducted using a Bruker Avance III 400 MHz NMR spectrometer (Bremen, Germany). Samples were dissolved in 10% D_2O/H_2O. The experiments were done as follows: ^{19}F NMR (400 MHz) proton decoupled, 128 scans, 298 K. The NMR data were processed and analyzed using MNova (14.2.3-29241). Data were pre-processed via auto-phase correction and an apodization of 1 Hz. Signal areas were received via auto-integration. For the validation, 1/x weighed calibration curves of five calibrants were established Linear calibration curves displayed a R^2>0.99. The overall bias was below 10%. RSD_R and RSD_T values were below 3% for all QC concentrations. Carry-over of samples can be excluded as new NMR tubes should be taken for each sample. No dedicated analysis of the stability of NMR samples were conducted as part of the validation.

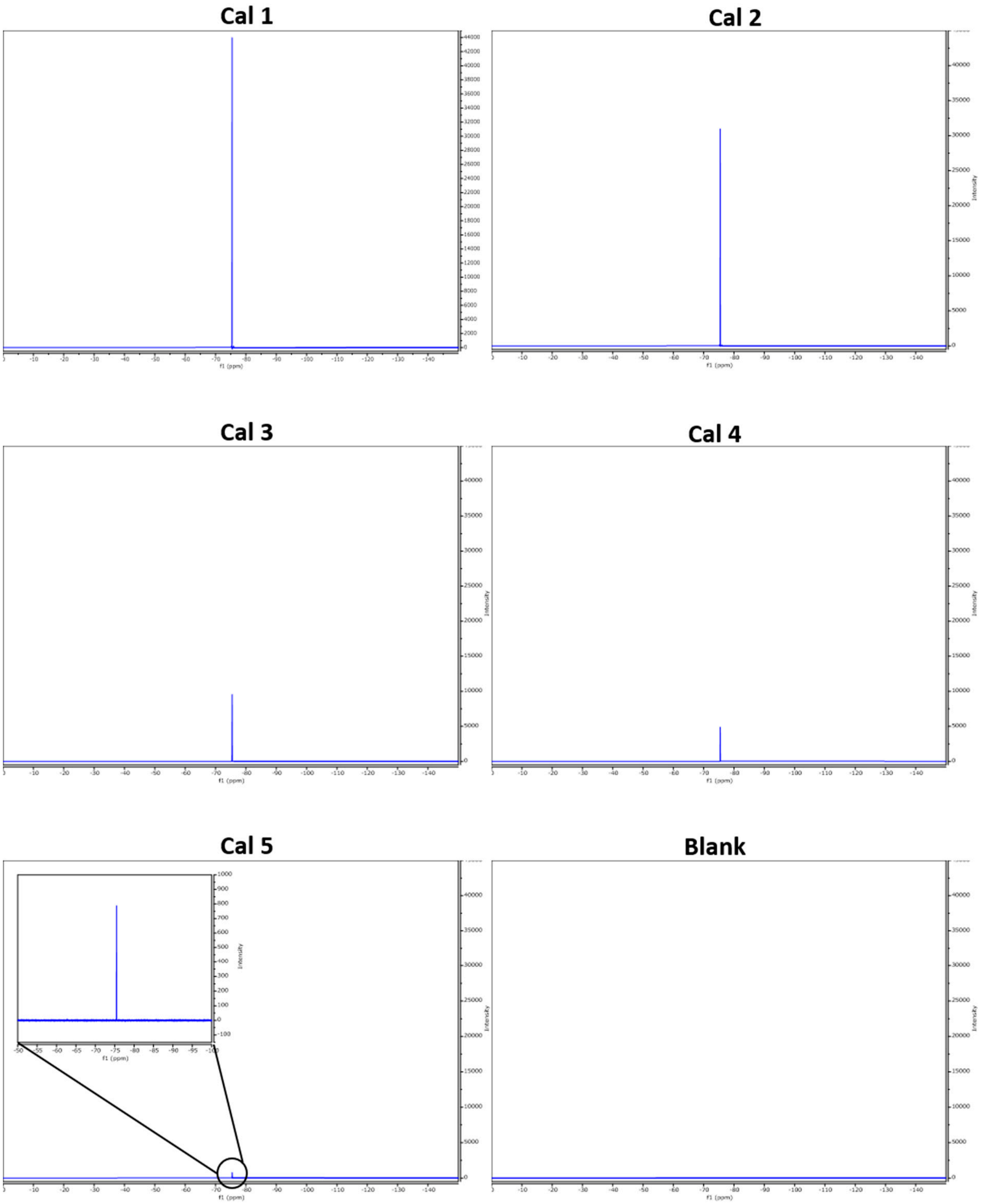

Figure S4: Calibration data day 1 ^{19}F-NMR

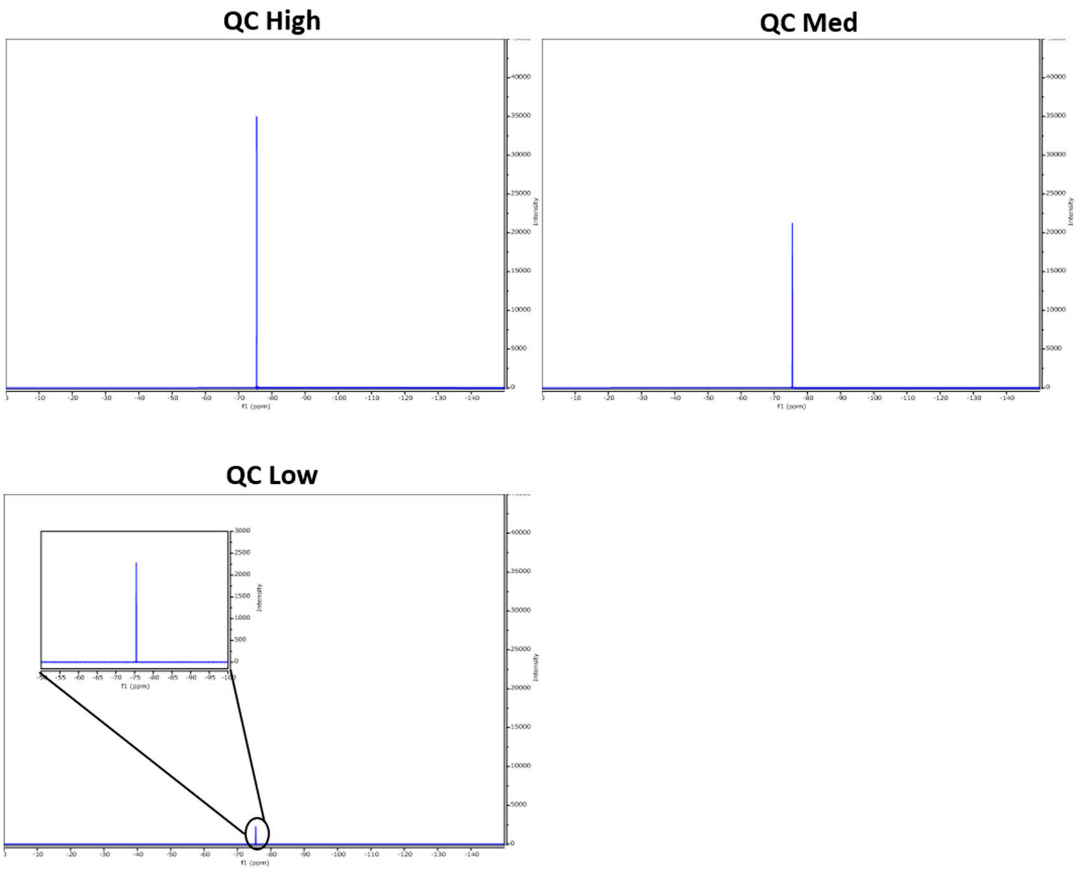

Figure S5: QC samples day 1 ^{19}F-NMR

Section S2.2: HPLC-ELSD

Measurements were performed on a VWR ELITE Lachrome Series LC modular system coupled to a L2455 diode array detector and a VWR Sedex Model 90 LT evaporative light scattering detector(Dietikon, Switzerland). For data evaluation, the OpenLab software (Version A. 04.08 - Agilent Technologies, Santa Clara, CA, USA) was used. Analyte separation was achieved by isocratic elution on an Acclaim Trinity P1 column (3 µm × 100 mm, SN: 002536, Thermo Fischer Scientific, Sunnyvale, CA, USA). As mobile phase, a mixture of 40% 20 mM ammonium formate in H_2O (pH 4.0) and 60% ACN with a flow rate of 0.7 mL/min was used. The column oven temperature was set to 30 °C and the autosampler cooled to 5 °C. The diode array detector was set to scan from 200 to 400 nm with an individual scan at 214 nm with a scanning rate of 400 ms. The ELSD settings were set to the following: 30 °C ELSD temperature, 3.0 bar nebulizer gas flow, 30 Hz sampling rate, gain of 7 for signal amplification with a filter of 1 s for noise reduction. All samples were dissolved in H2O. Calibrator and QC samples for method validation were measured as singlets with an injection volume of 20 µL.

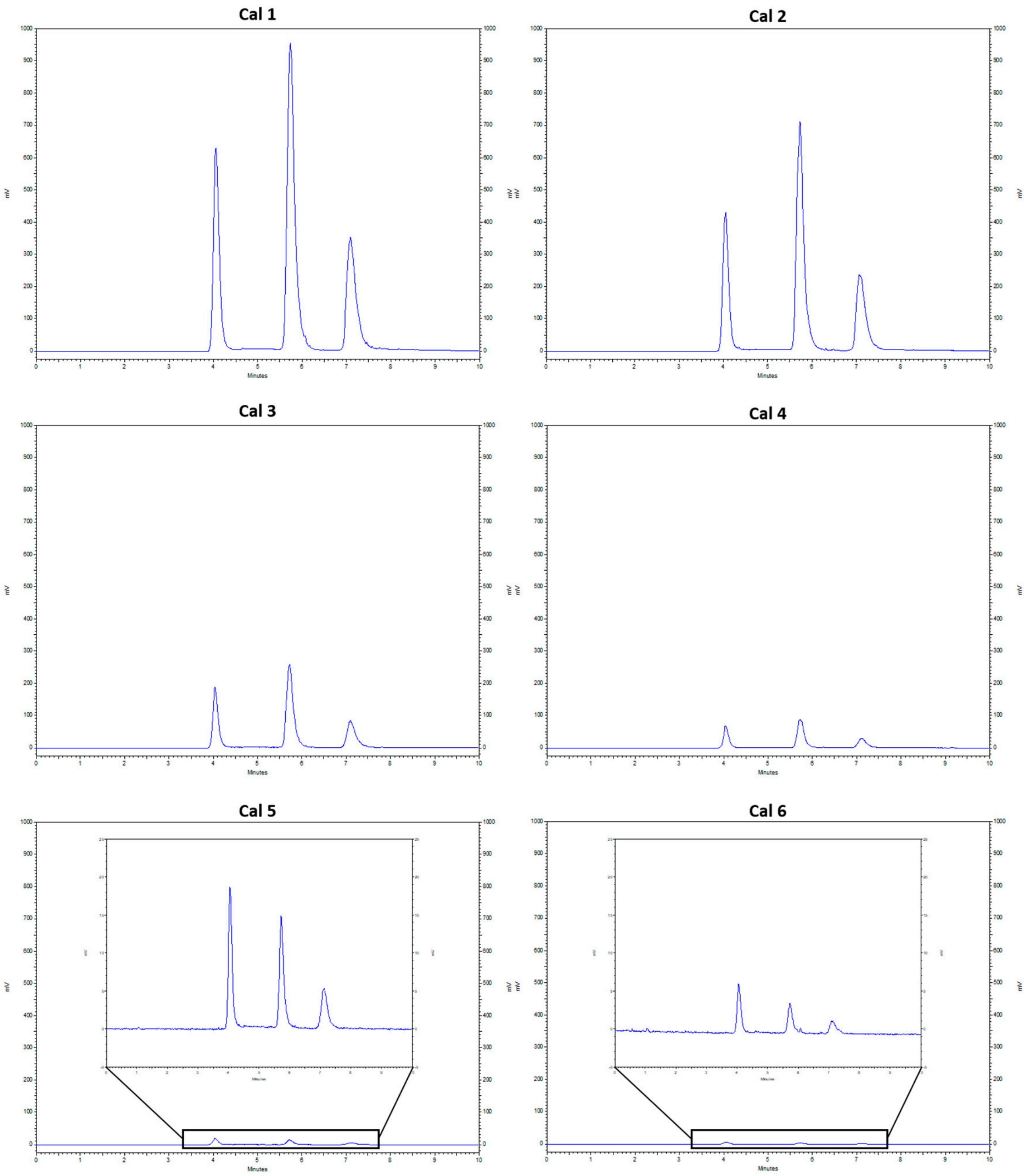

Figure S6: Calibration data day 1 HPLC-ELSD

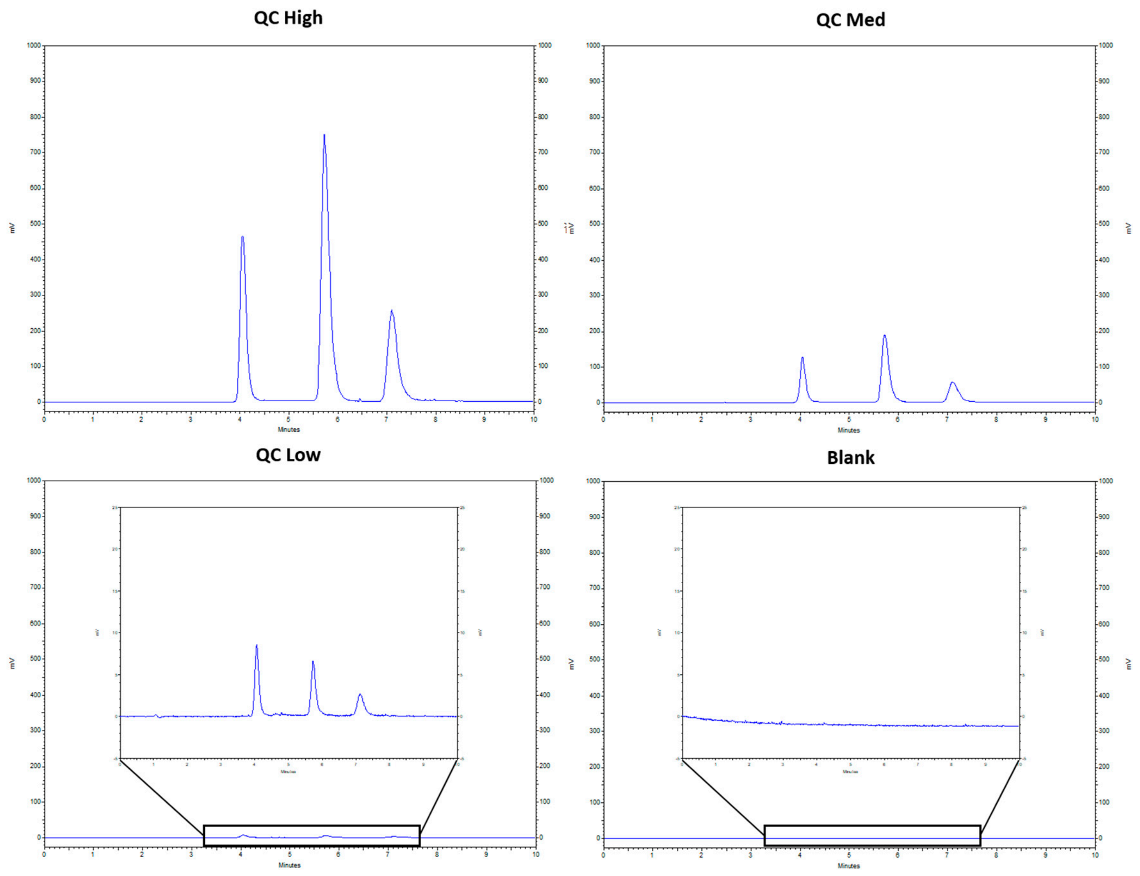

Figure S7: QC samples day 1 HPLC-ELSD

Section S2.3: FT-IR

IR spectra were acquired on a Spectrum Two™ Spectrometer (PerkinElmer Inc., Waltham, MA, USA) equipped with a LiTaO3 MIR detector. Spectra were recorded at a wavelength from 4000 cm^{-1} to 600 cm^{-1} with a resolution of 1 cm^{-1}. For each measurement, eight scans were performed. All samples were diluted in H_2O. For analysis 100 µL of sample solution were pipetted into an ATR volatiles cup. Before every measurement series, H_2O was measured as background. Between measurements, the cup was cleaned thoroughly with H_2O. Data was evaluated with the PerkinElmer SpectrumIR (17.7.2.1630) software. Data processing and visualisation was conducted using Python (3.10.10) and NumPy (1.23.5), Pandas (1.5.3), SciPy (1.9.3) and Matplotlib (3.7.1). Data was smoothed using the Savitzky-Golay filter with a window length of 11 and second polynomial order.

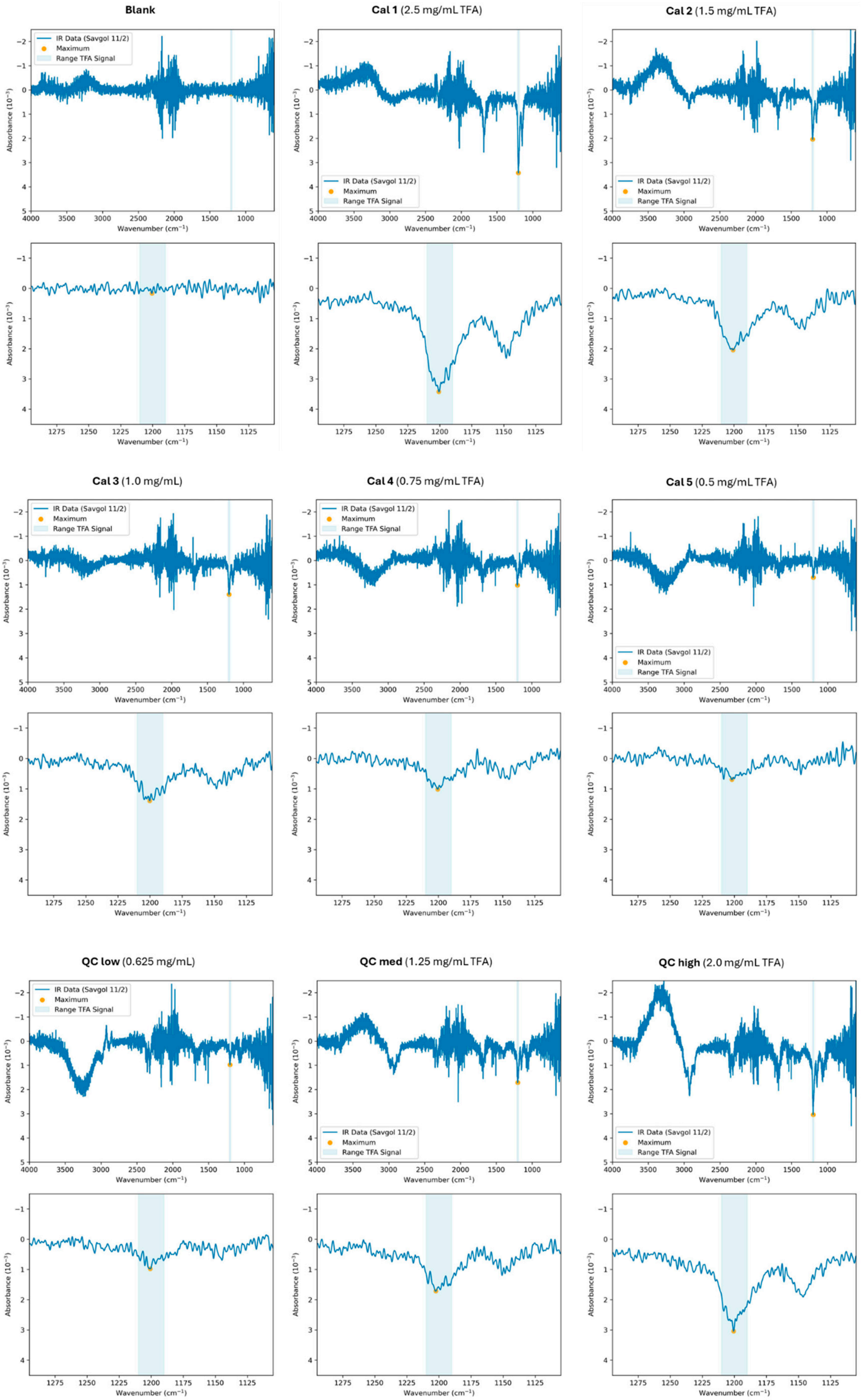

Figure S8: Calibration data FT-IR

Python script for IR data evaluation:

```
import pandas as pd
import numpy as np
import os
import matplotlib.pyplot as plt
from scipy.signal import savgol_filter

filepath = 'Y:\IRData'

max_range = [1190,1210]
plot_range = [1105, 1295]
header_list = ['Sample', 'Data Max', 'Data Wavenumber']
results_list = []

for filename in os.listdir(filepath):
    f = os.path.join(filepath, filename)
    df = pd.read_csv(f, delimiter=';', skiprows = 1)

    # Assigning x and y to the wavenumber and absorption
    x = df['cm-1'].values
    y = df['A'].values * 1000

    sg_window = 11
    sg_polyorder = 2

    # Smooth the data using Savitzky-Golay-Filter
    x_smooth = x
    y_smooth = savgol_filter(y, window_length=sg_window, polyorder=sg_polyorder)

    # Define the region for TFA signal to find maximum absorbance
    x_start, x_end = max_range

    # Reduce x_smooth and y_smooth to the specified range
    mask = (x_smooth >= x_start) & (x_smooth <= x_end)
    x_filtered = x_smooth[mask]
    y_filtered = y_smooth[mask]

    # Find the maximum y-value within the specified range
    max_index = np.argmax(y_filtered)
    max_x = x_filtered[max_index]
    max_y = y_filtered[max_index]

    # Insert result in results dataframe
    row_result = [filename, max_y, max_x]
    dict_result = dict(zip(header_list, row_result))
    results_list.append(dict_result)

    # Plot the smooth curve and maximum
    plt.plot(x_smooth, y_smooth, label='IR Data (Savgol 11/2)')
    plt.scatter(max_x, max_y, color = 'orange', label = 'Maximum')
    plt.axvspan(x_start, x_end, color='lightblue', alpha=0.3, label = 'Range TFA Signal')
    plt.legend()
```

```
    plt.xlabel(r'Wavenumber (cm$^{-1}$)')
    plt.ylabel(r'Absorbance ($10^{-3}$)')
    plt.title(filename)
    plt.xlim(600, 4000)
    plt.ylim(-2.5, 5)
    plt.gca().invert_xaxis()
    plt.gca().invert_yaxis()
    plt.savefig(filename + '.jpg', dpi=300, format='jpg', bbox_inches='tight')
    plt.show()

#Define empty results data frame
df_results = pd.DataFrame(results_list)

# Save Results as Excel Sheet
df_results.to_excel(filepath)
```

Section S2.4: Summary Validation Results

Table S1: Summary of method validation

**Methods**	**Analyte**	**Model**	**Weighting**	**Calibration Range [µg/mL]**	**Range of R^2**	**QC Low**					**QC Med**					**QC High**				
						Theoretic conc. [µg/mL]	Calc. conc. [µg/mL]	Bias [%]	RSDR [%]	RSDT [%]	Theoretic conc. [µg/mL]	Calc. conc. [µg/mL]	Bias [%]	RSDR [%]	RSDT [%]	Theoretic conc. [µg/mL]	Calc. conc. [µg/mL]	Bias [%]	RSDR [%]	RSDT [%]
HPLC-ELSD	Na	Quadratic	x	0.87-34.9	0.9979-0.9993	1.716	1.640	-4.5	2.9	7.4	10.297	9.661	-6.2	1.4	3.1	27.5	28.3	2.9	1.30	1.62
HPLC-ELSD	TFA	Quadratic	x	10.2-409.5	0.9978-0.9999	20.523	17.812	13.2	2.5	6.6	123.135	130.097	5.7	3.2	8.6	328.4	328.1	-0.1	3.93	6.64
HPLC-ELSD	Cl	Quadratic	x	1.35-53.8	0.9978-0.9997	2.647	2.837	7.2	1.2	4.0	15.879	15.643	-1.5	1.3	3.8	42.4	43.8	3.3	1.47	2.09

F^{19}-NMR	TFA	Linear	1/x	50.7-2536.5	0.9993-0.9997	100.775	110.333	9.5	1.1	2.8	1007.750	1043.529	3.6	2.1	2.9	2015.5	2020.1	0.2	1.73	1.73
FT-IR	TFA	Linear	x	503-2514	0.9947-0.9998	613.05	710.35	15.9	16.8	19.6	1226.1	1282.9	4.6	9.7	11.7	2043.5	2119.6	3.7	4.49	10.04

Section S3**: Salt Exchange**

Section S3.1: ^{19}F-NMR

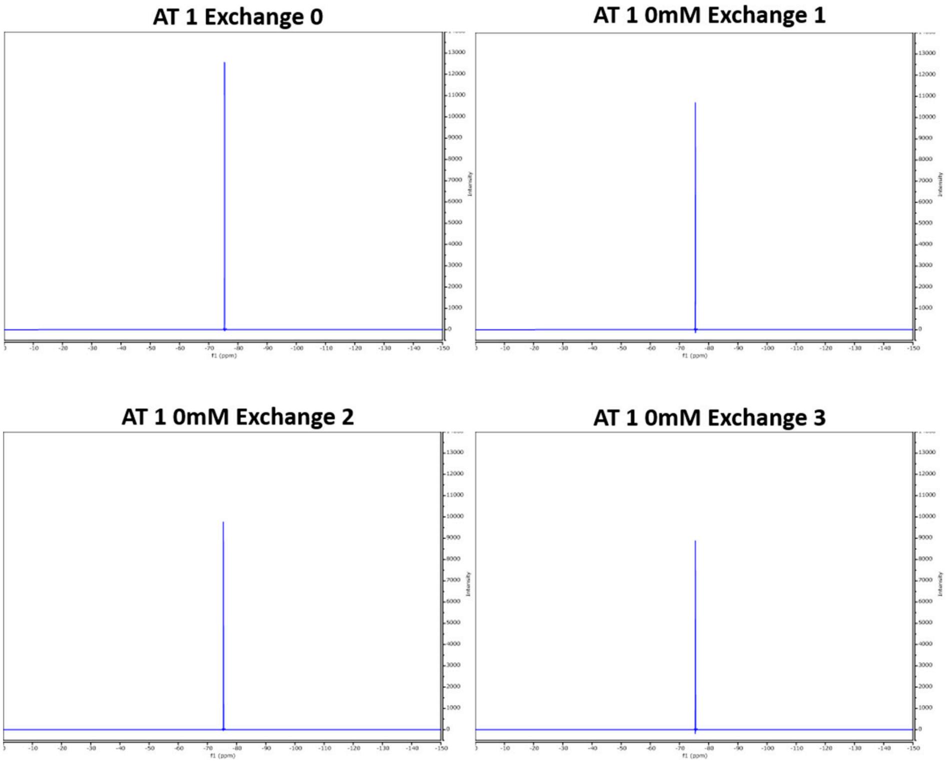

Figure S9: ^{19}F-NMR spectra of AT1 with 0 mM HCl exchange

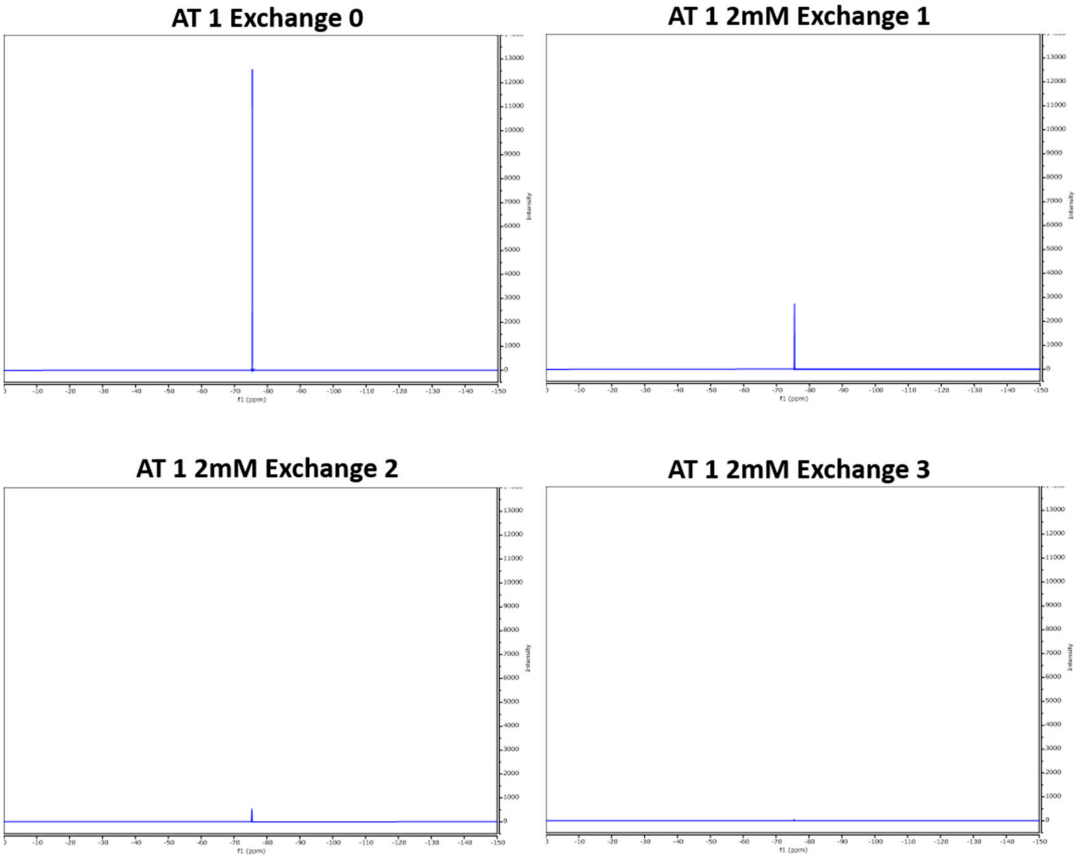

Figure S10: ^{19}F-NMR spectra of AT1 with 2 mM HCl exchange

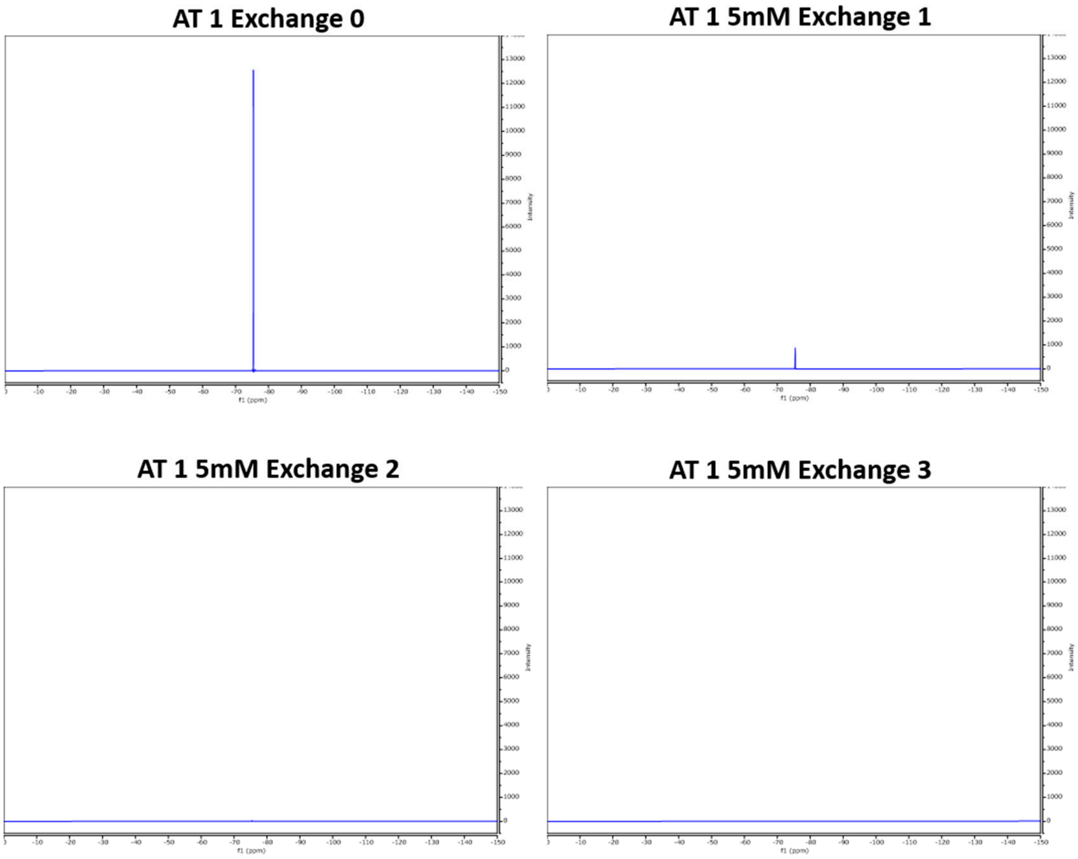

Figure S11: ^{19}F-NMR spectra of AT1 with 5 mM HCl exchange

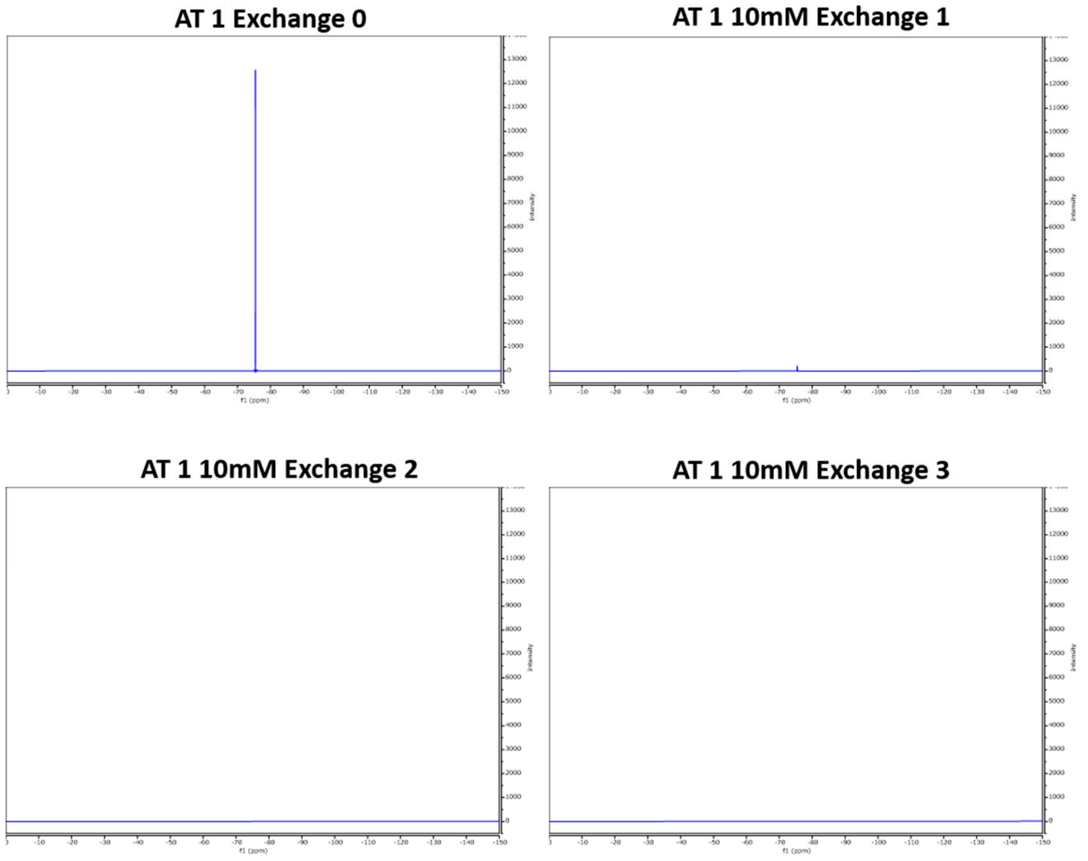

Figure S12: ^{19}F-NMR spectra of AT1 with 10 mM HCl exchange

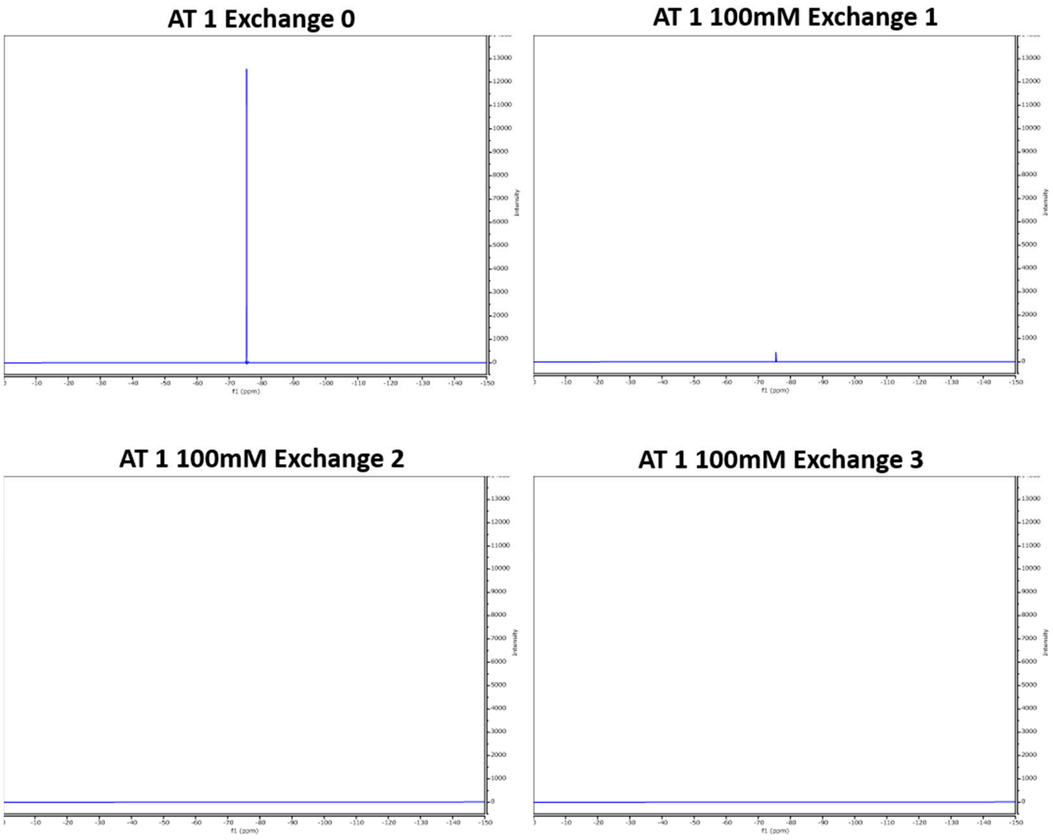

Figure S13: ^{19}F-NMR spectra of AT1 with 100 mM HCl exchange

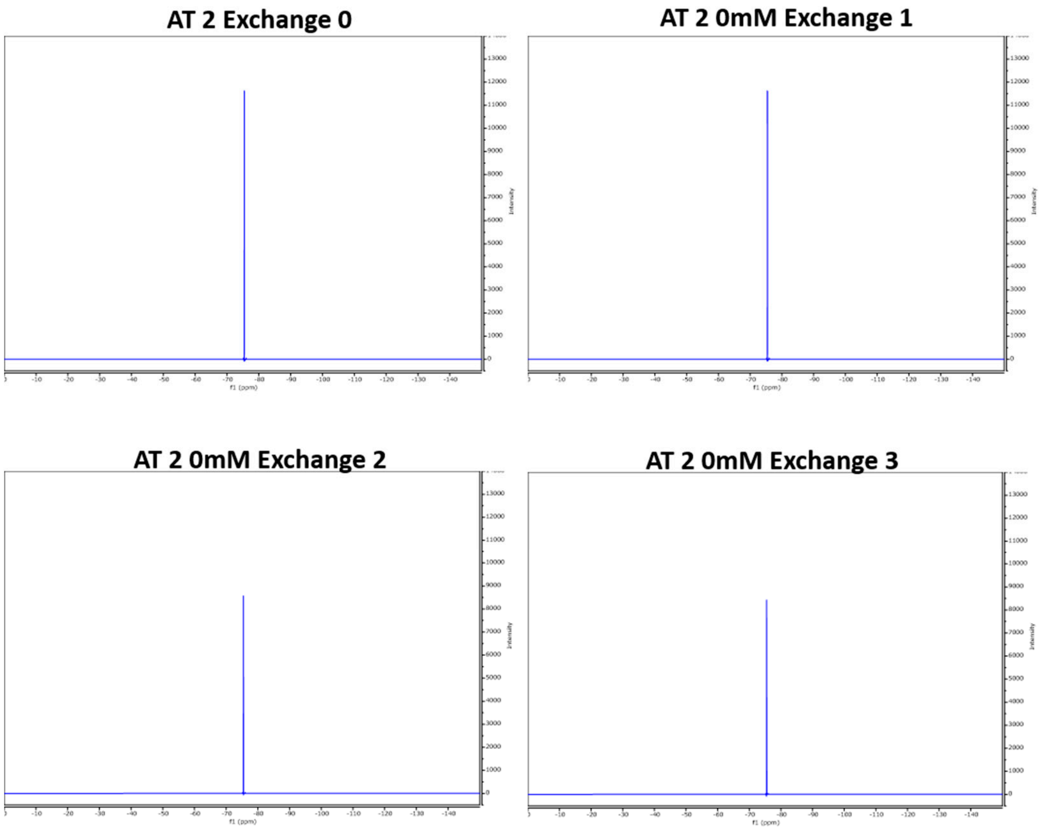

Figure S14: ^{19}F-NMR spectra of AT2 with 0 mM HCl exchange

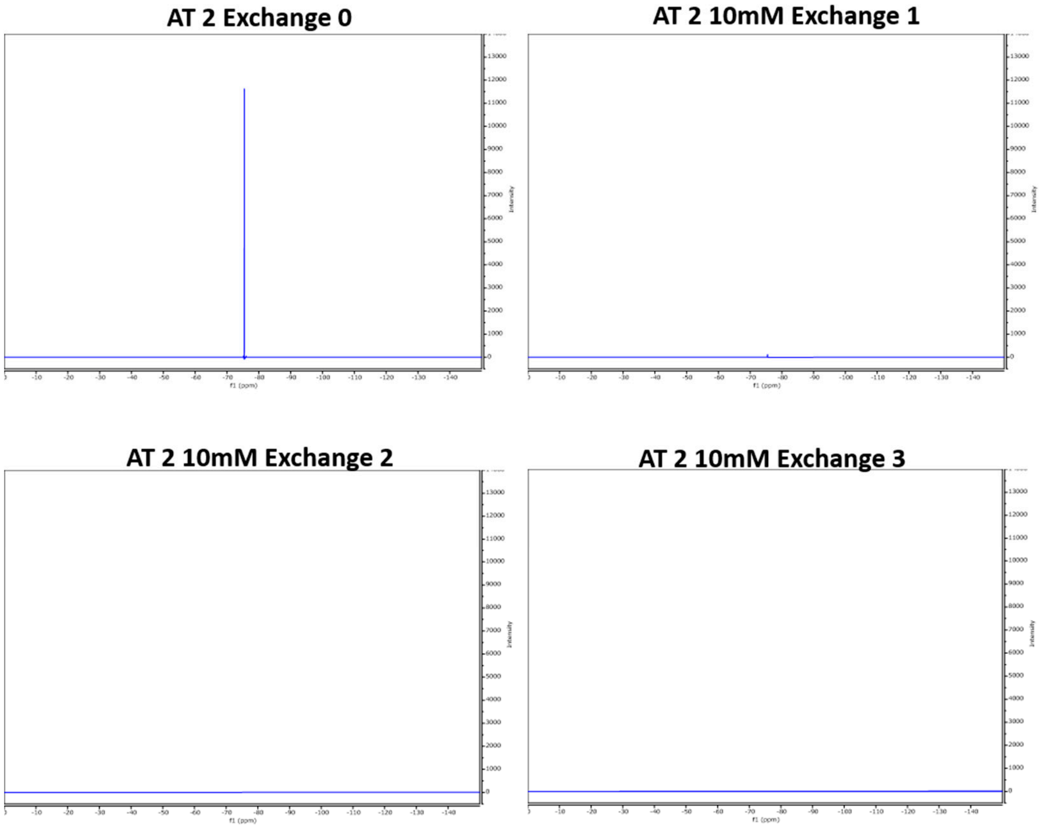

Figure S15: ^{19}F-NMR spectra of AT2 with 10 mM HCl exchange

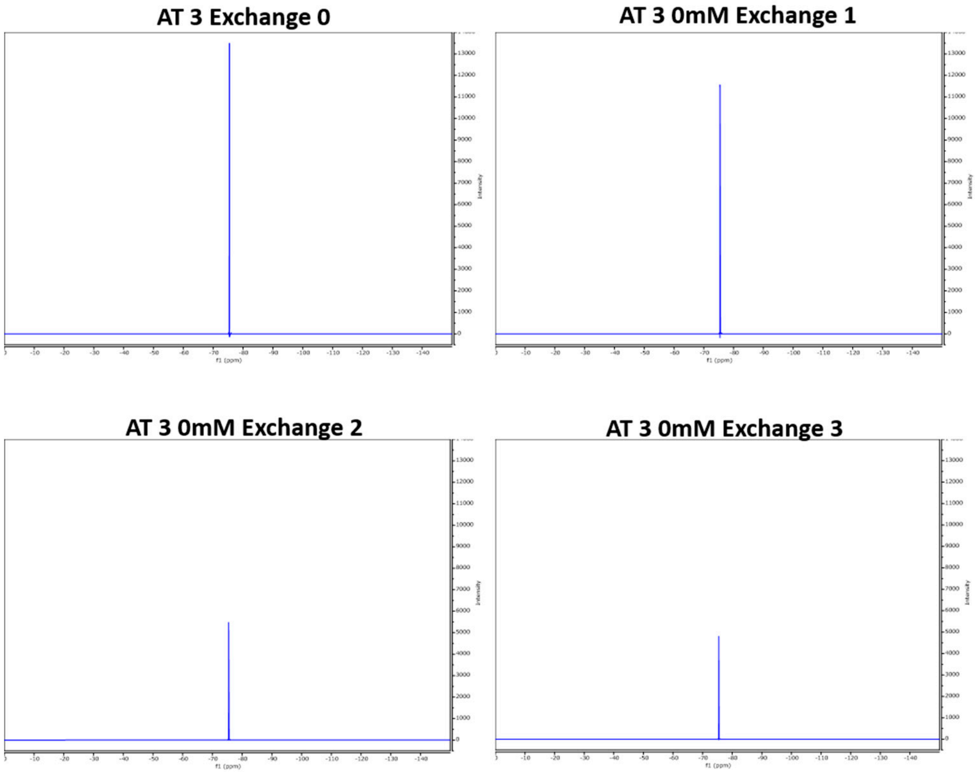

Figure S16: ^{19}F-NMR spectra of AT3 with 0 mM HCl exchange

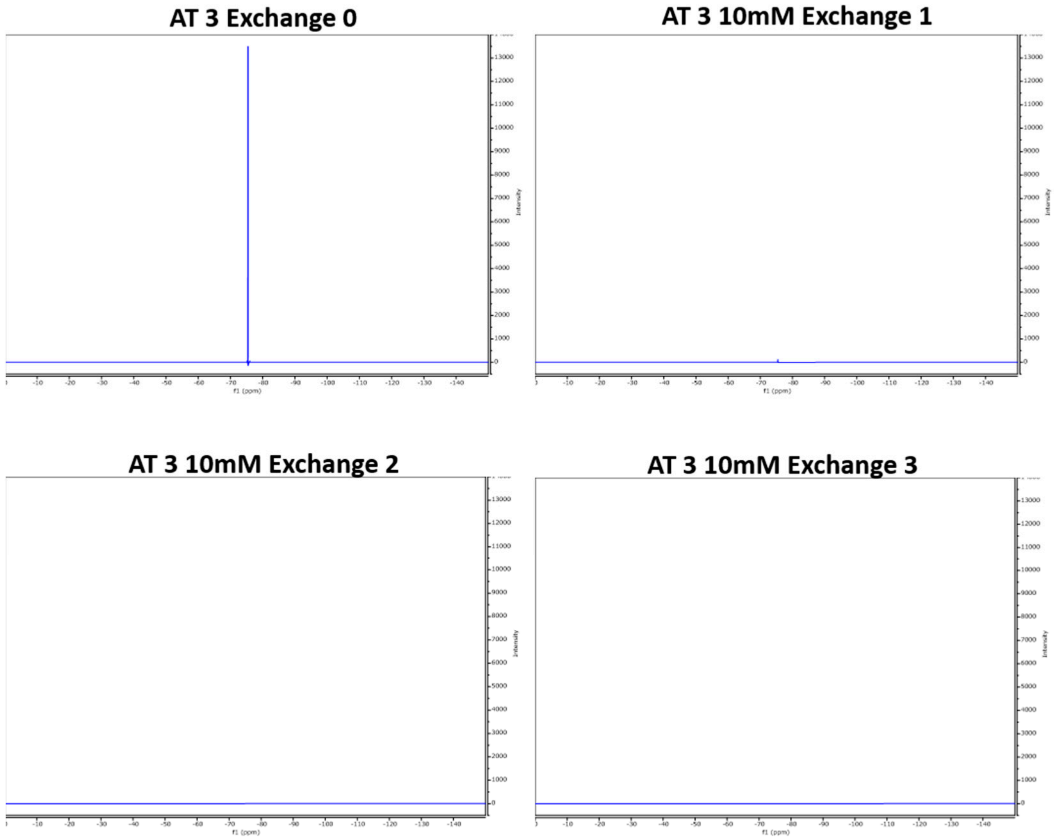

Figure S17: ^{19}F-NMR spectra of AT3 with 10 mM HCl exchange

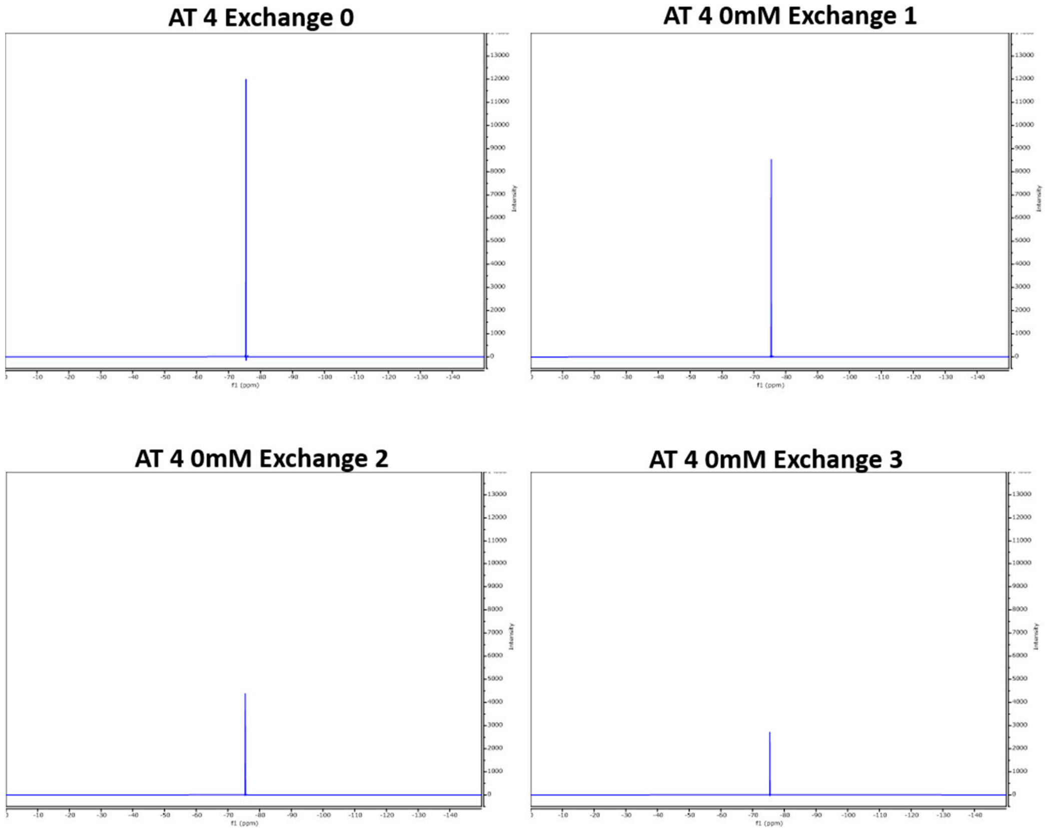

Figure S18: ^{19}F-NMR spectra of AT4 with 0 mM HCl exchange

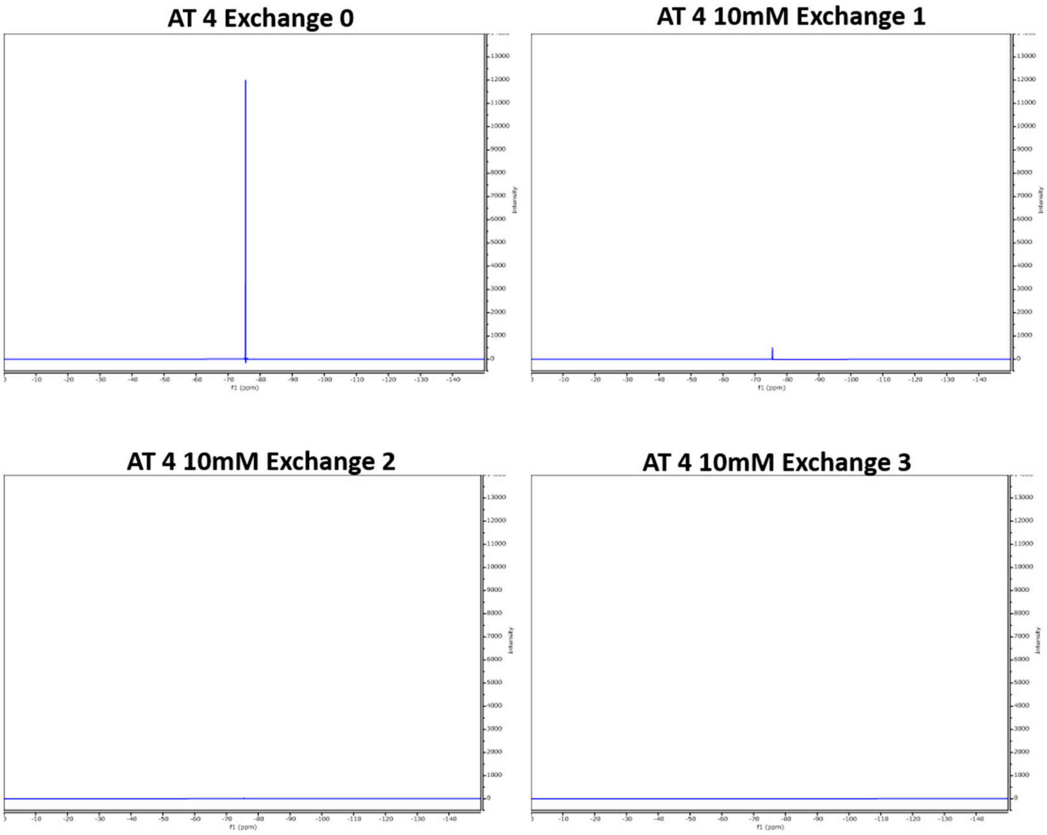

Figure S19: ^{19}F-NMR spectra of AT4 with 10 mM HCl exchange

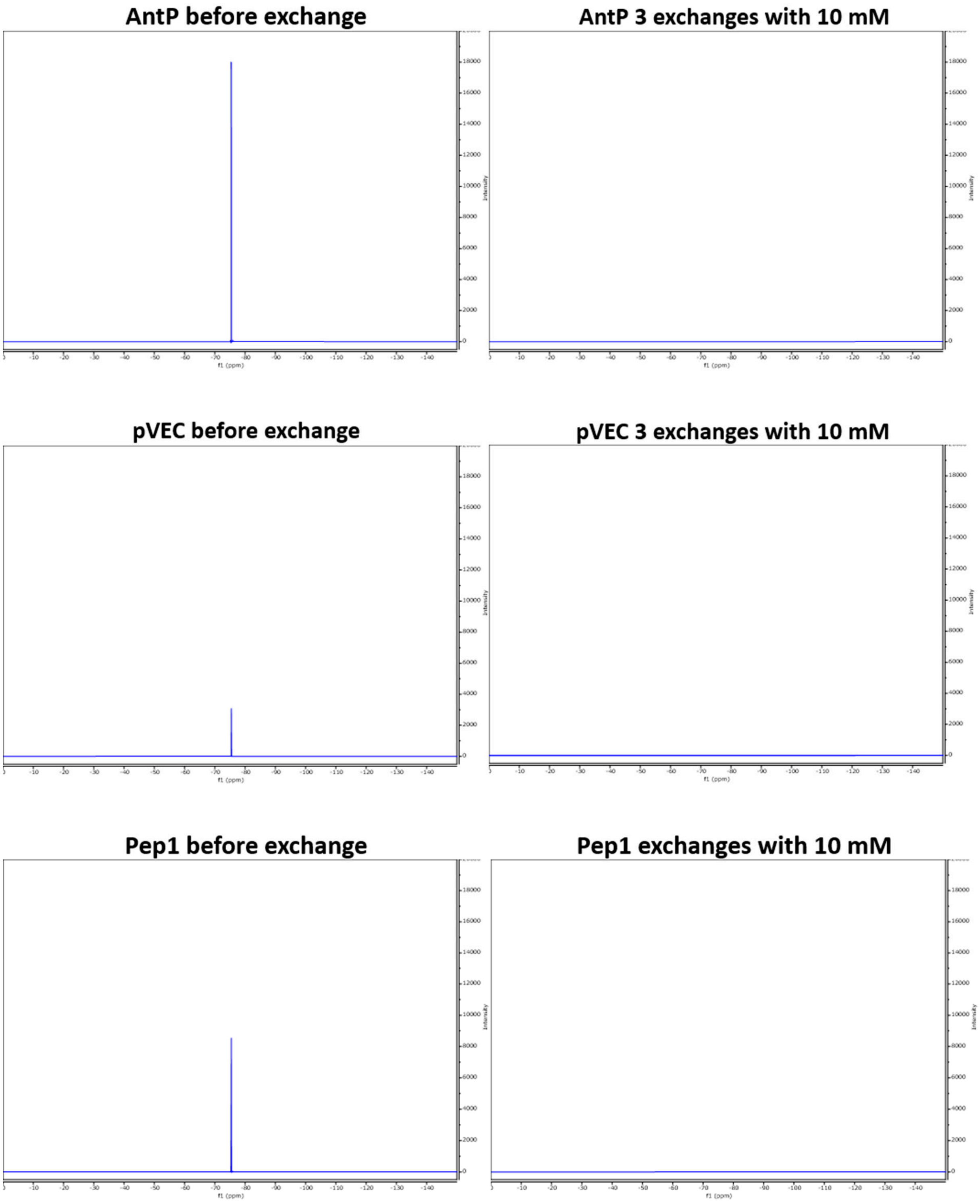

Figure S20: ^{19}F-NMR spectra of CPPs before and after three counterion exchange cycles with 10 mM HCl

Section S3.2: HPLC-ELSD

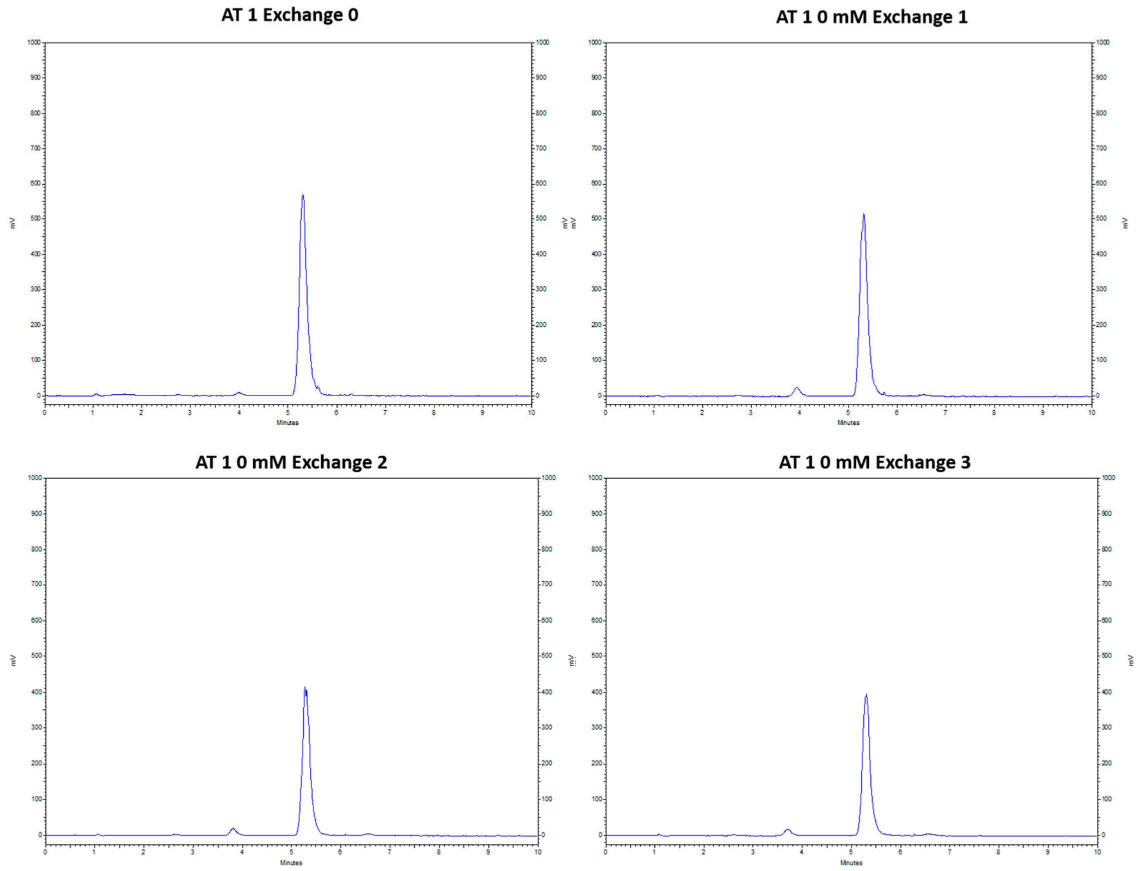

Figure S21: HPLC-ELSD chromatograms of AT1 for 0 mM HCl exchange

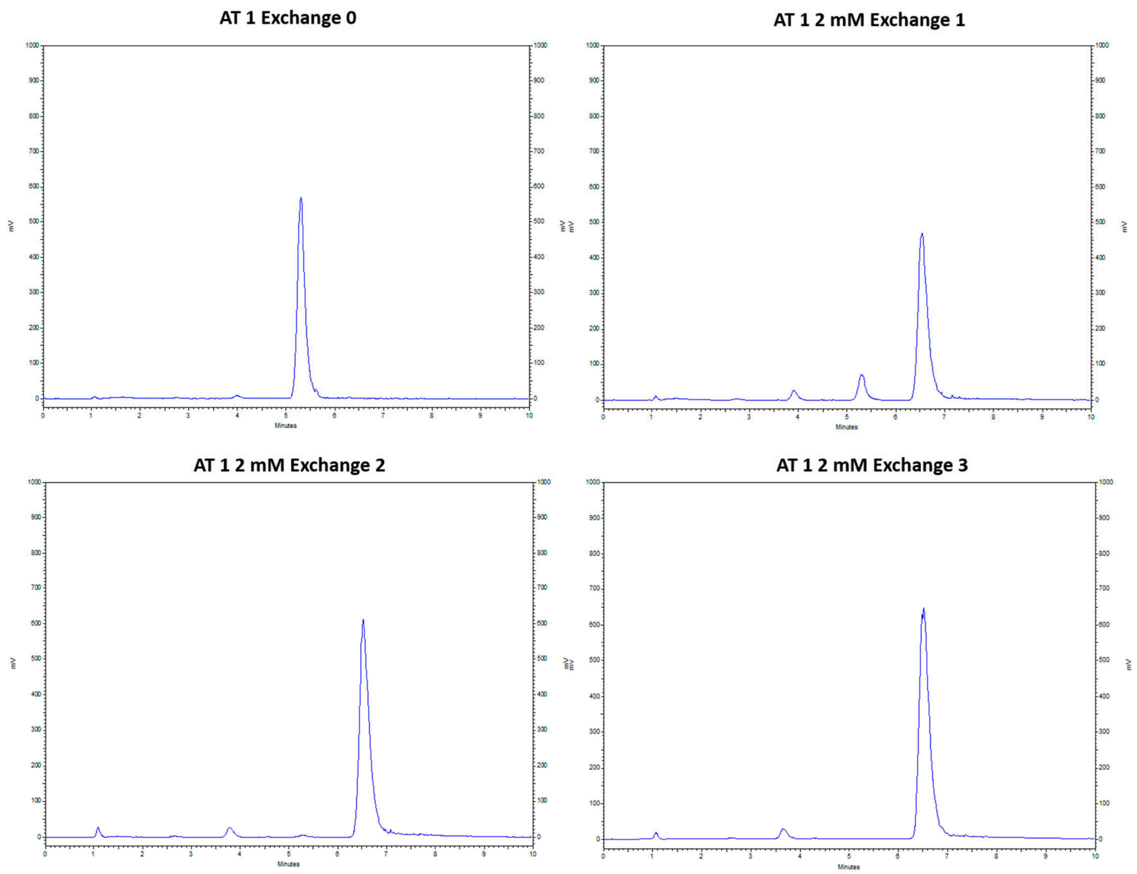

Figure S22: HPLC-ELSD chromatograms of AT1 for 2 mM HCl exchange

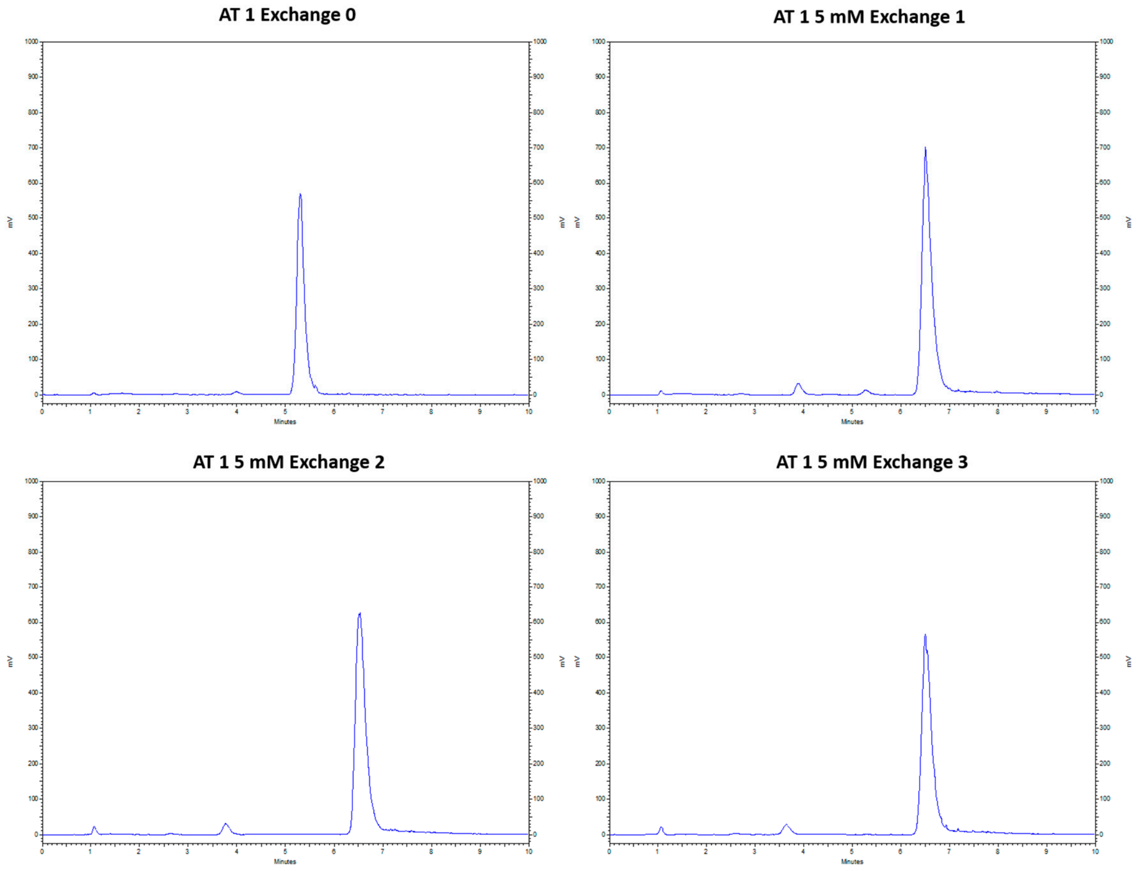

Figure S23: HPLC-ELSD chromatograms of AT1 for 5 mM HCl exchange

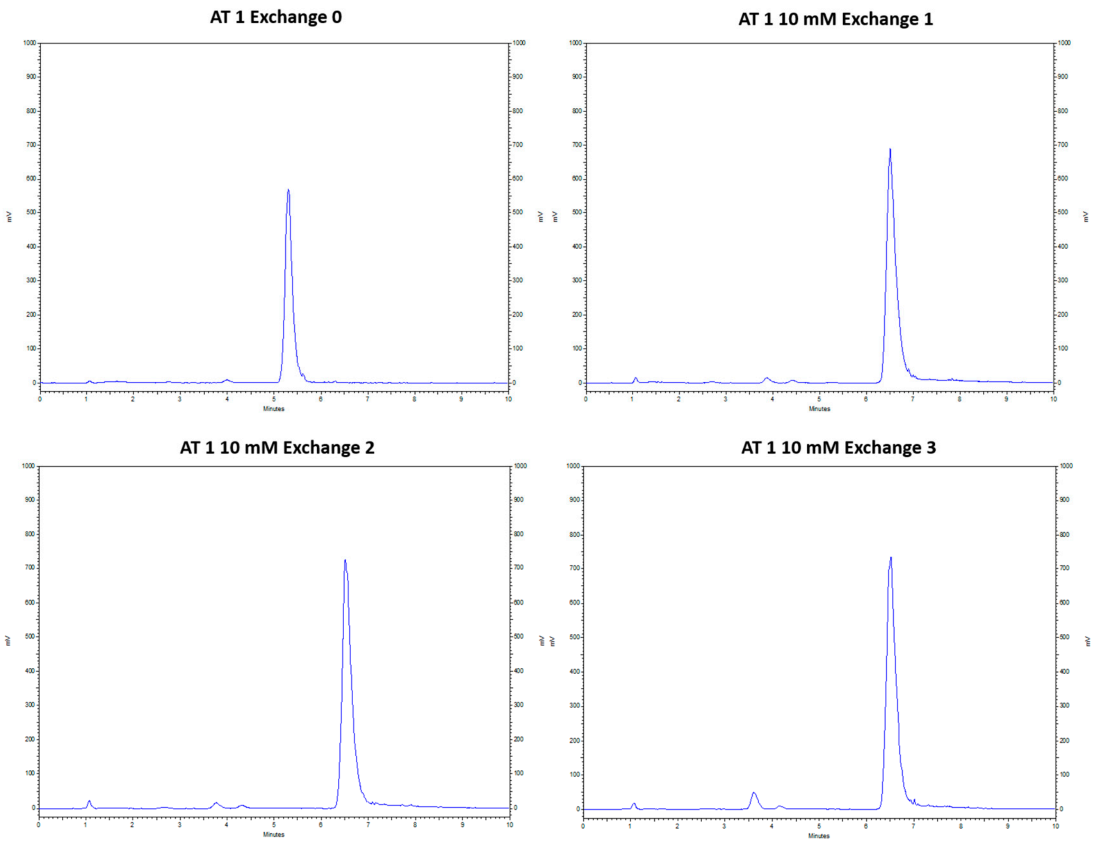

Figure S24: HPLC-ELSD chromatograms of AT1 for 10 mM HCl exchange

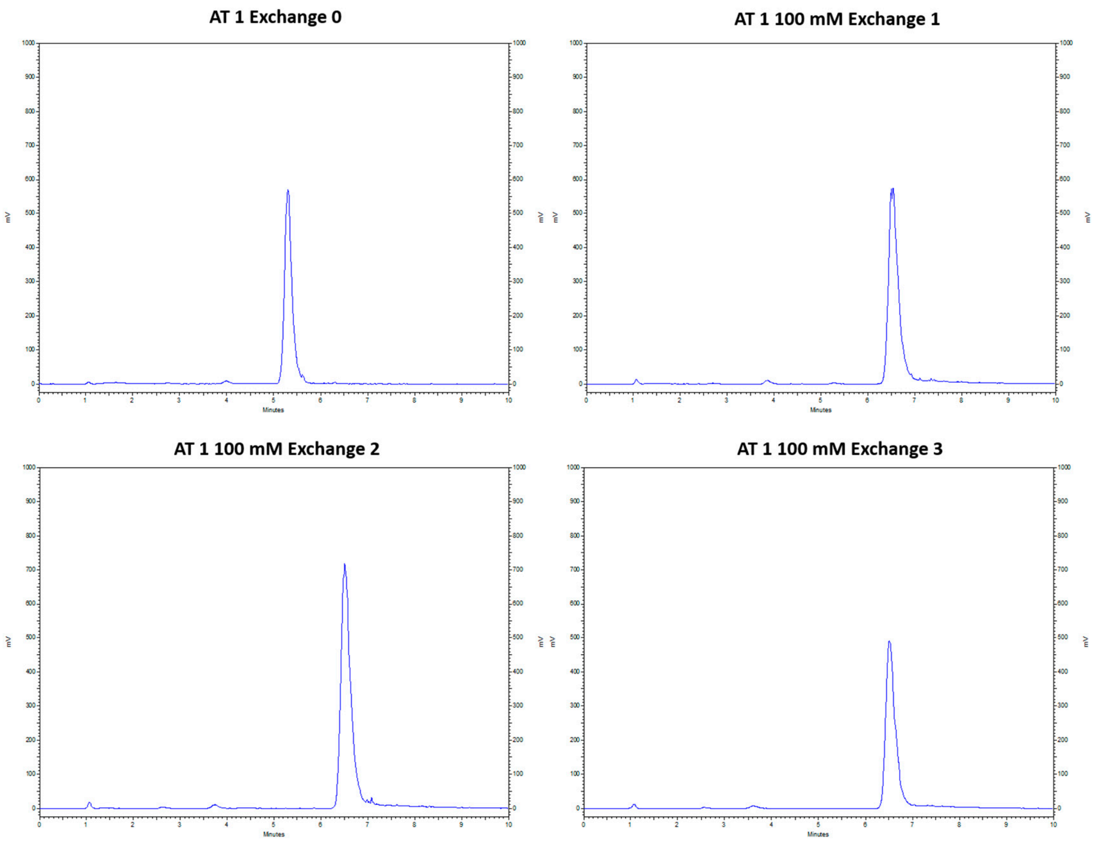

Figure S25: HPLC-ELSD chromatograms of AT1 for 100 mM HCl exchange

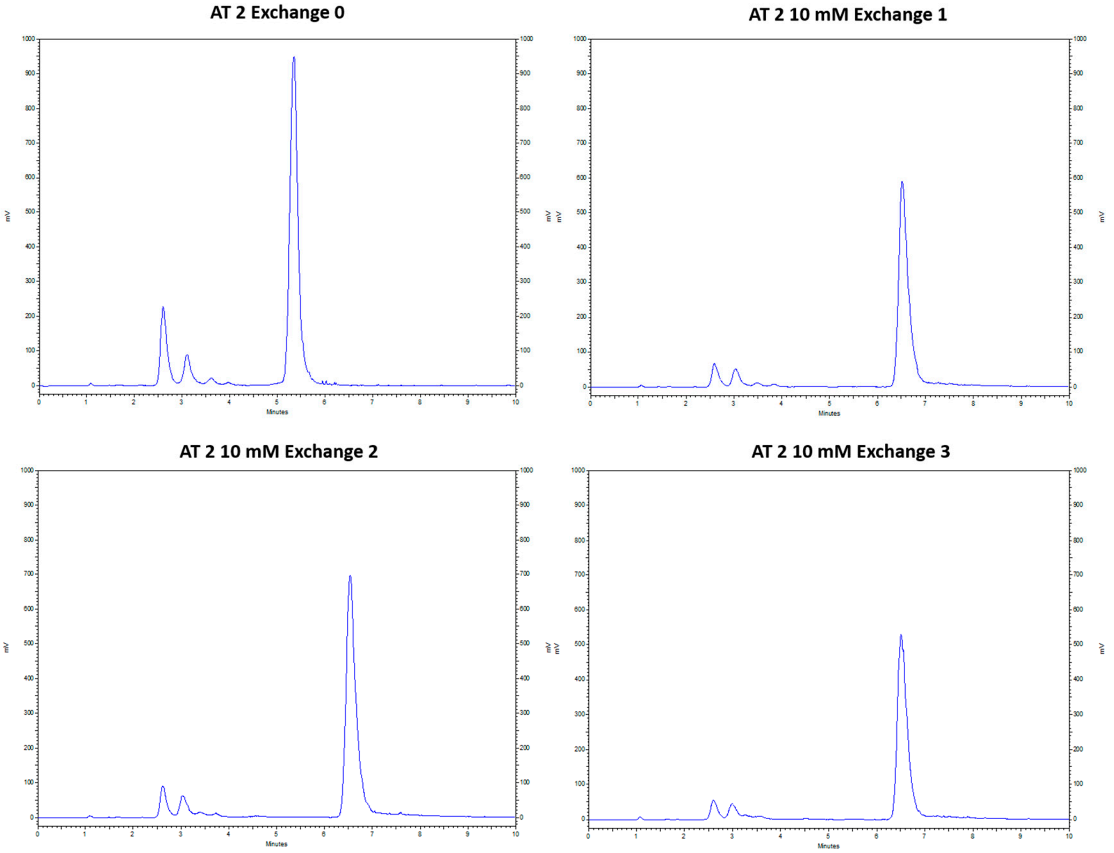

Figure S26: HPLC-ELSD chromatograms of AT2 for 10 mM HCl exchange

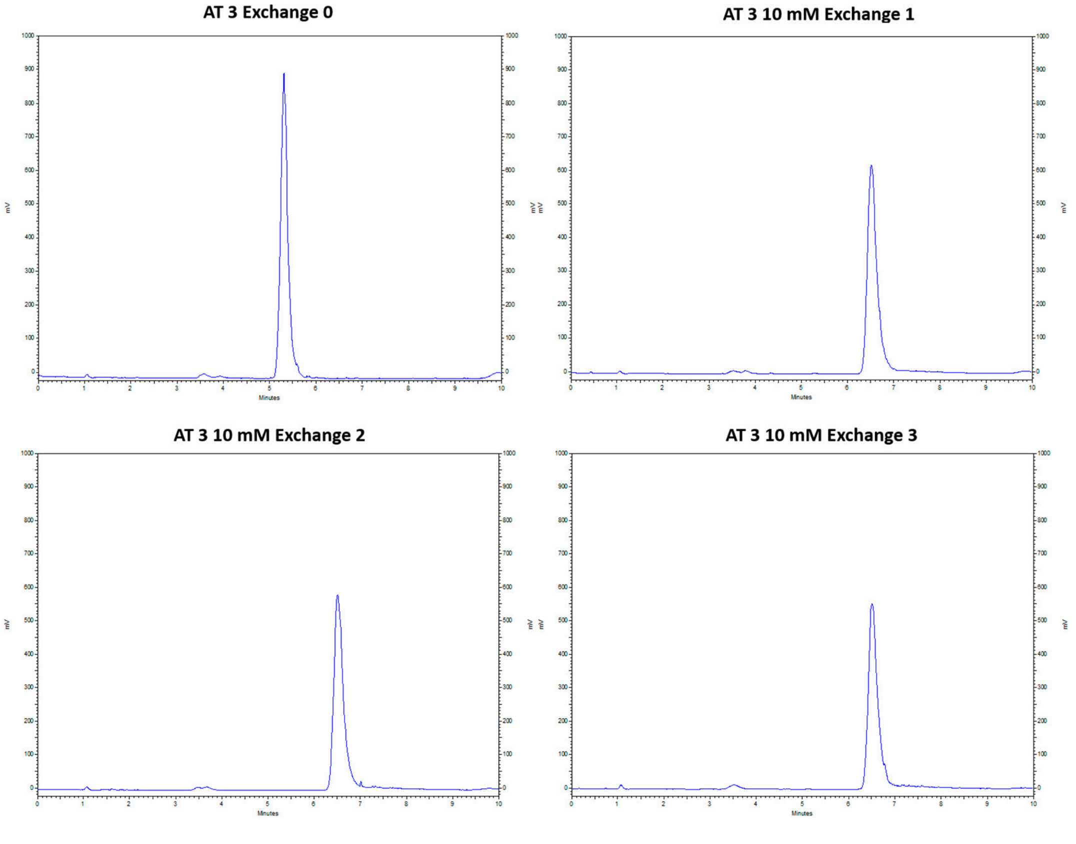

Figure S27: HPLC-ELSD chromatograms of AT3 for 10 mM HCl exchange

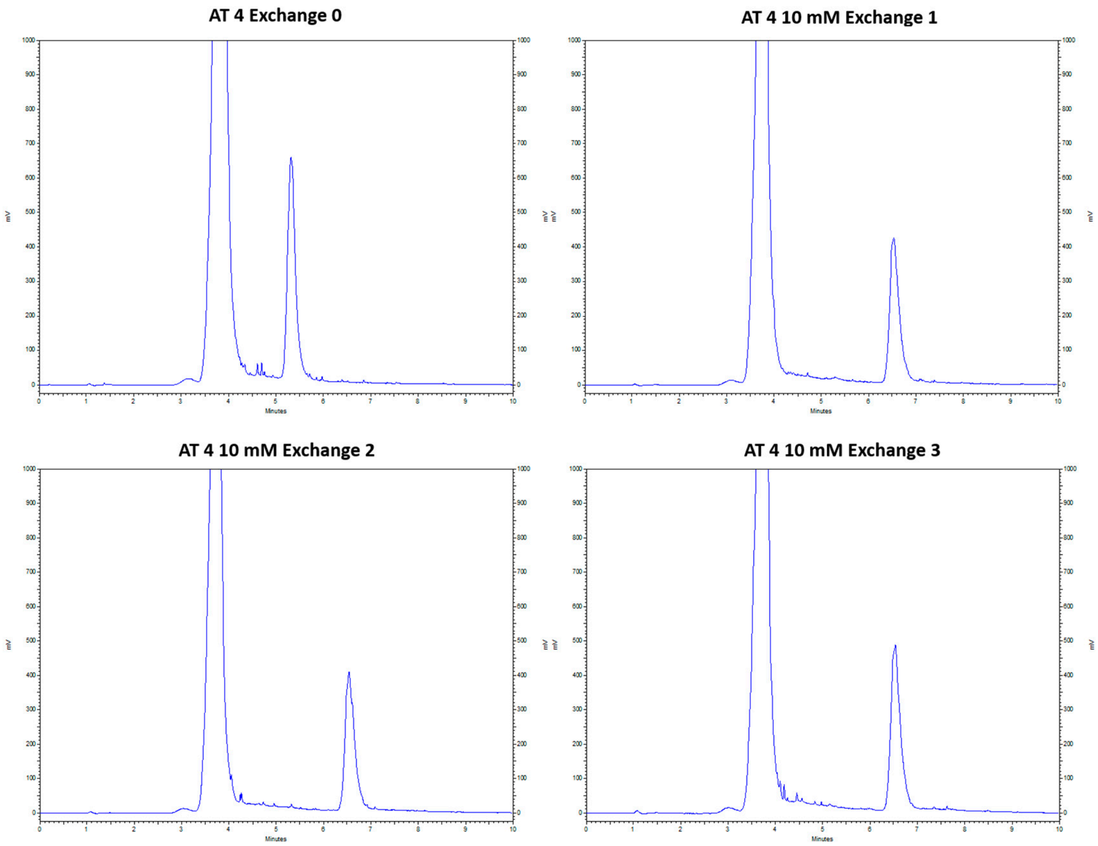

Figure S28: HPLC-ELSD chromatograms of AT4 for 10 mM HCl exchange

Section S3.3: FT-IR

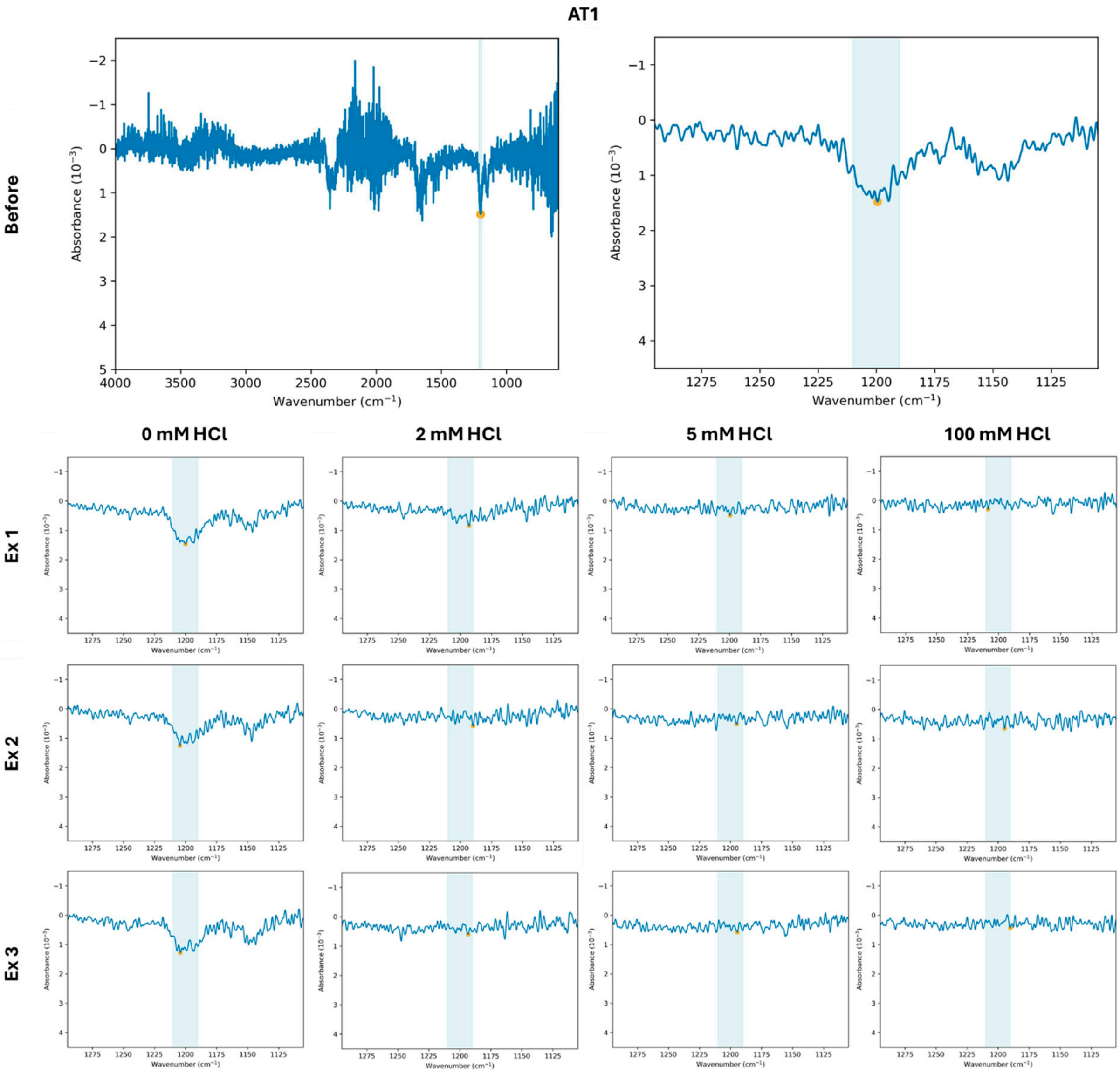

Figure S29: FT-IR data of salt exchange of AT1 with different concentrations of HCl

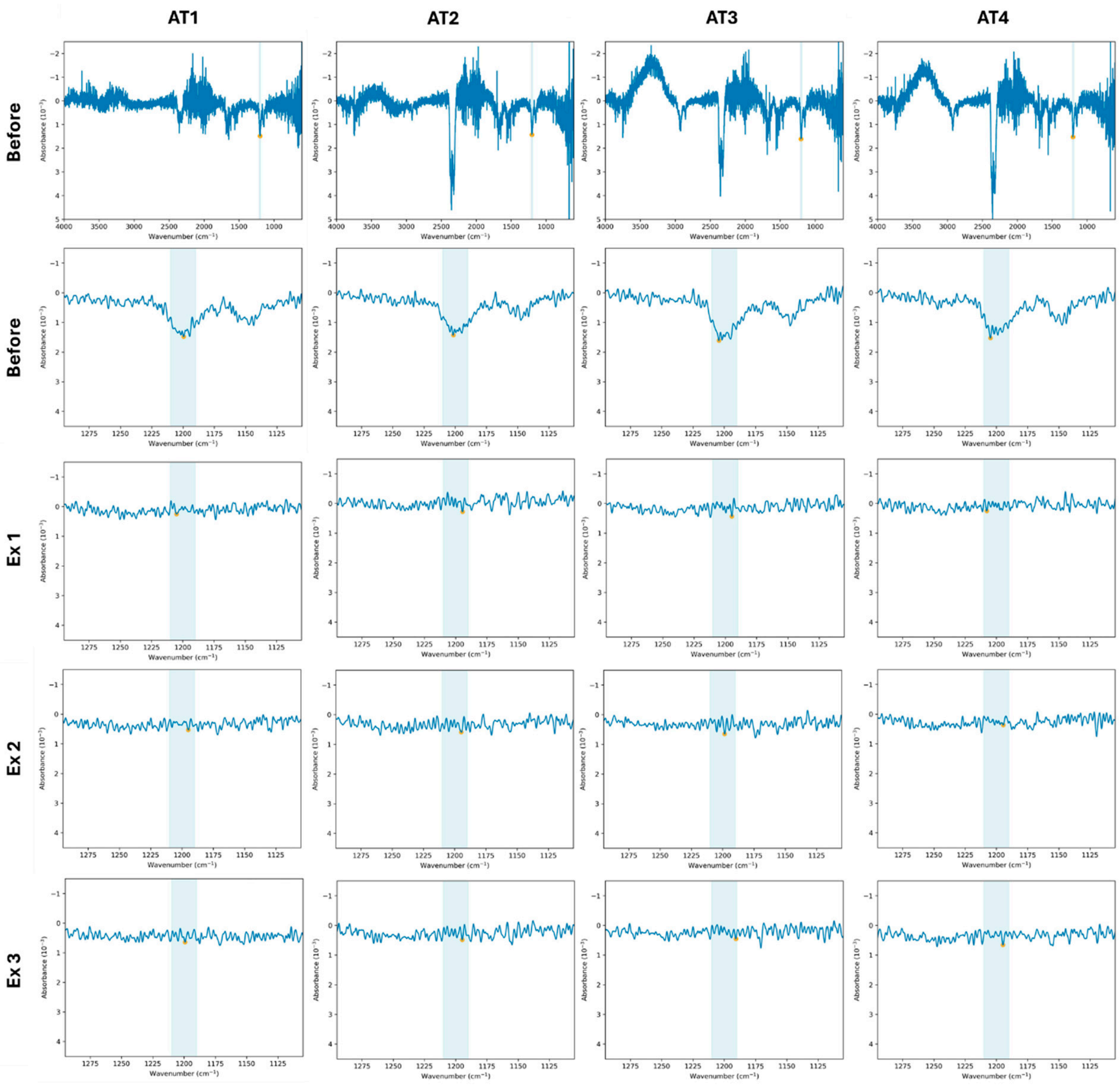

Figure S30: FT-IR data of salt exchange of AT1-4 with 10 mM HCl

Section S3.4: Purity

Table S2: Peptide purity determined by HPLC-UV before and after HCl exchange

	Peptide	AT 1					AT 2	AT 3	AT 4	AntP	Pep1	pVEC
	Conc. aq. HCl for salt ex-change (mM)	0	2	5	10	100	10	10	10	10	10	10
Before Exchange	Purity (%) mean	97.88					97.30	94.66	95.59	86.70*	93.50*	100.00*
	Purity (%) std	1.62					0.24	0.07	0.34			
Exchange 1	Purity (%) mean	97.86	97.83	98.16	97.79	98.79	97.16	94.52	95.54			
	Purity (%) std	1.58	1.63	1.94	1.16	0.05	0.09	0.05	0.32			
Exchange 2	Purity (%) mean	97.77	97.93	97.90	98.90	98.82	97.05	94.38	95.41			
	Purity (%) std	1.71	1.57	1.53	0.06	0.02	0.02	0.16	0.30			
Exchange 3	Purity (%) mean	96.57	96.51	96.83	98.95	98.88	97.09	94.45	95.48	88.10*	87.53*	100.00*
	Purity (%) std	1.70	1.34	1.57	0.01	0.03	0.03	0.28	0.11			

* n = 1

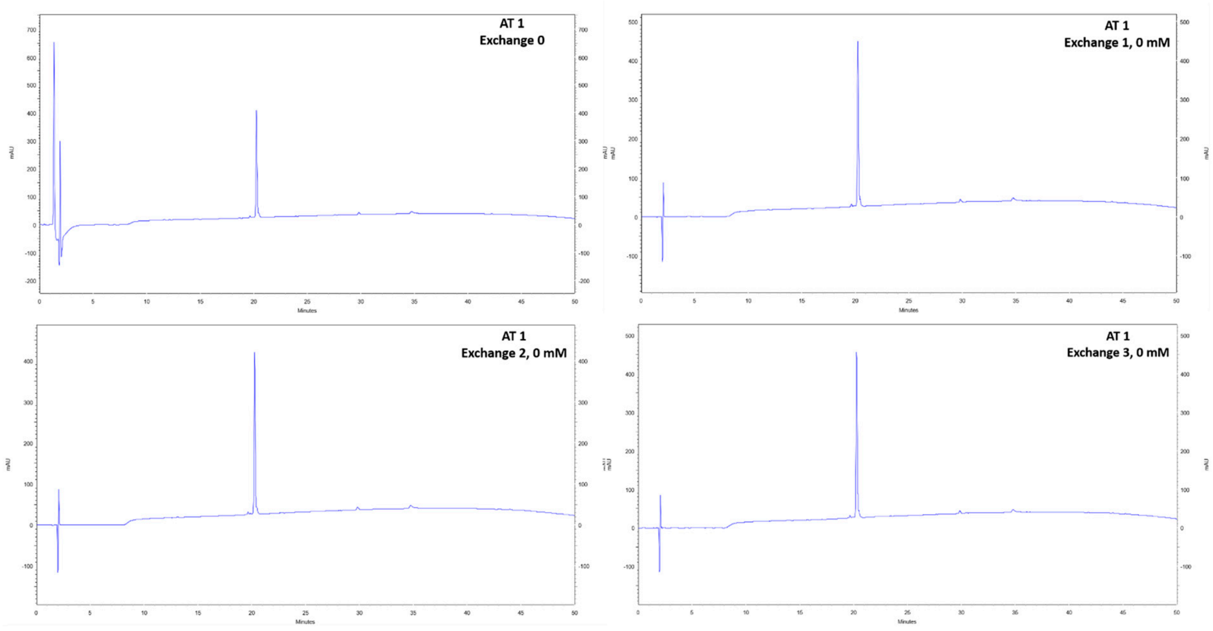

Figure S31: Purity of AT1 over three counterion exchanges with 0 mM HCl

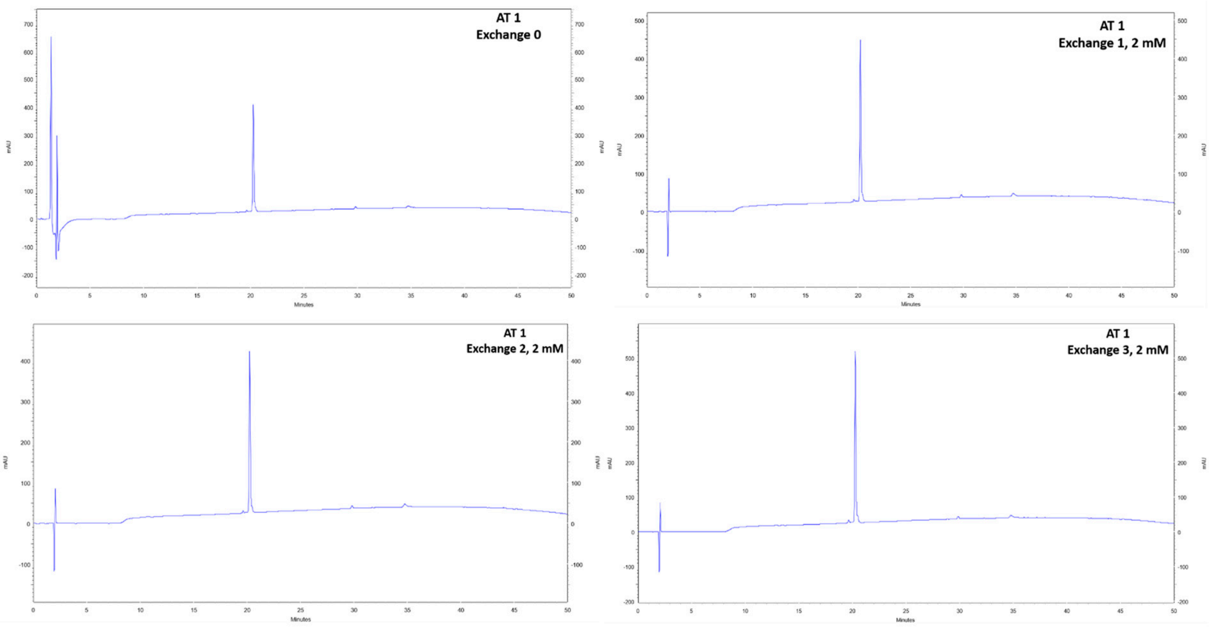

Figure S32: Purity of AT1 over three counterion exchanges with 2 mM HCl

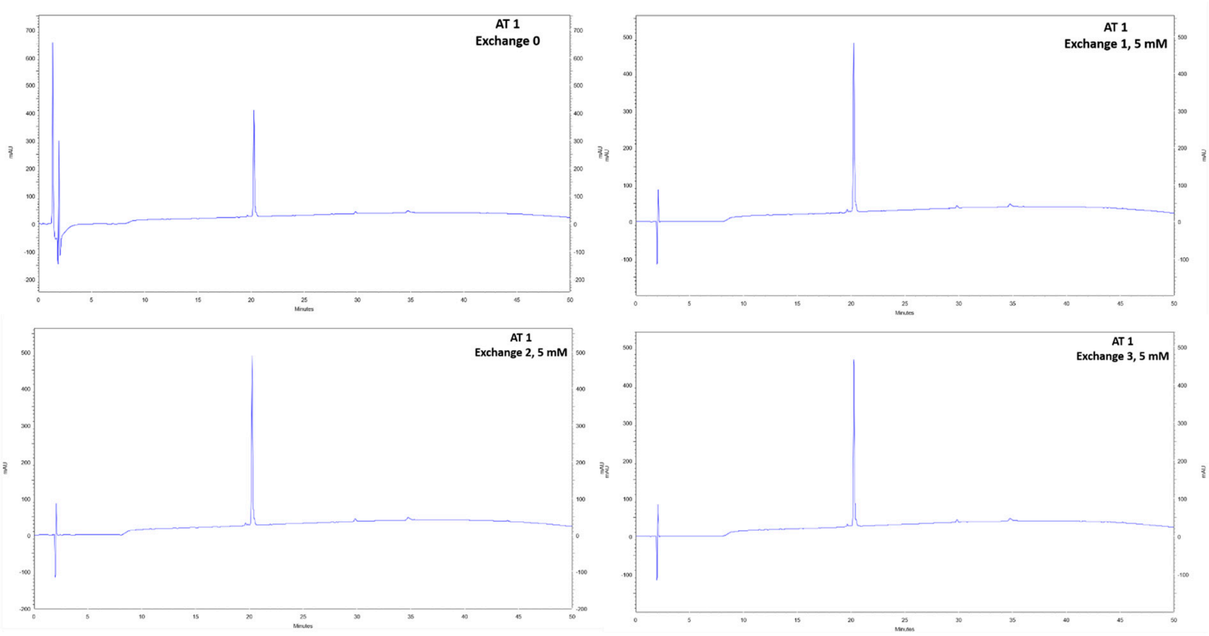

Figure S33: Purity of AT1 over three counterion exchanges with 5 mM HCl

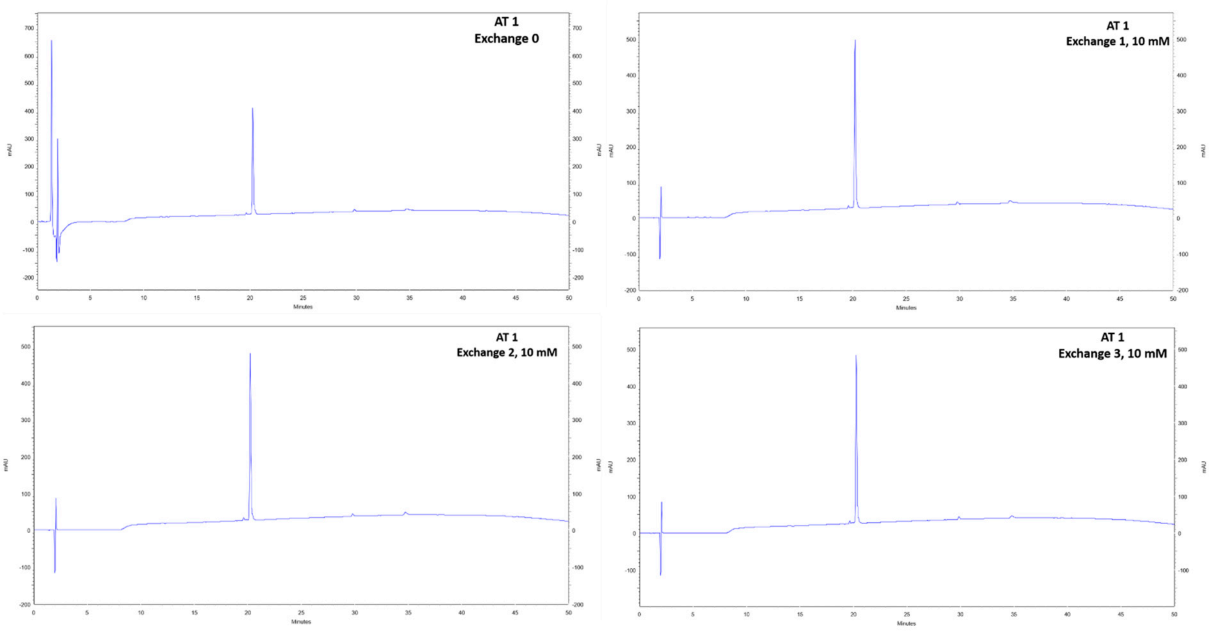

Figure S34: Purity of AT1 over three counterion exchanges with 10 mM HCl

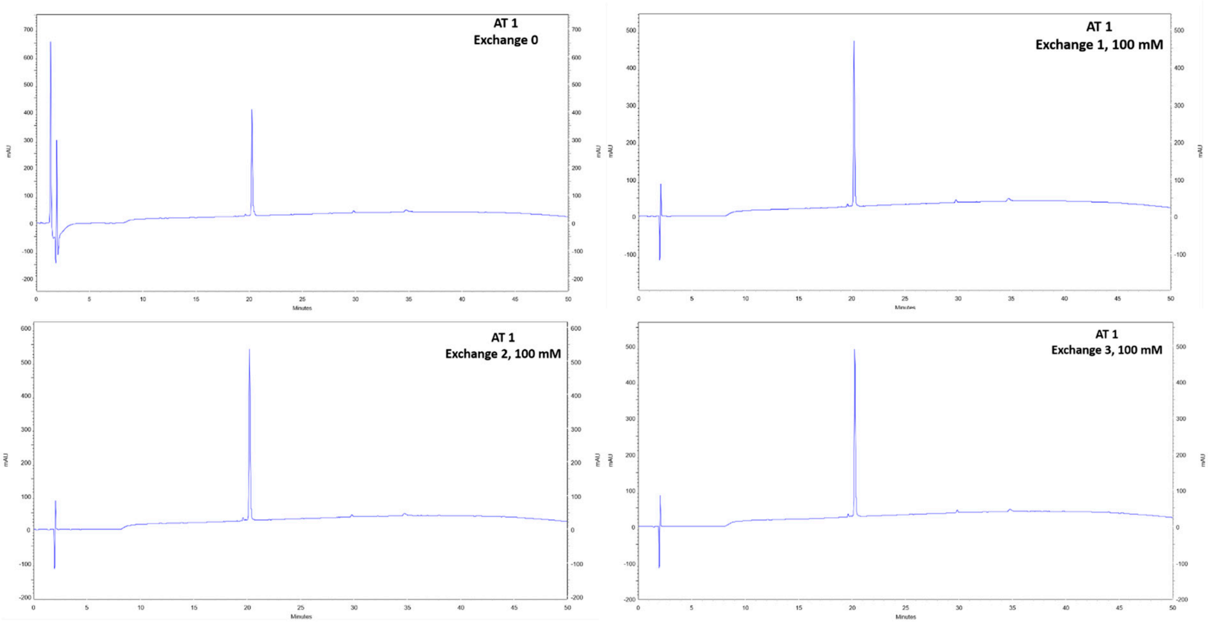

Figure S35: Purity of AT1 over three counterion exchanges with 100 mM HCl

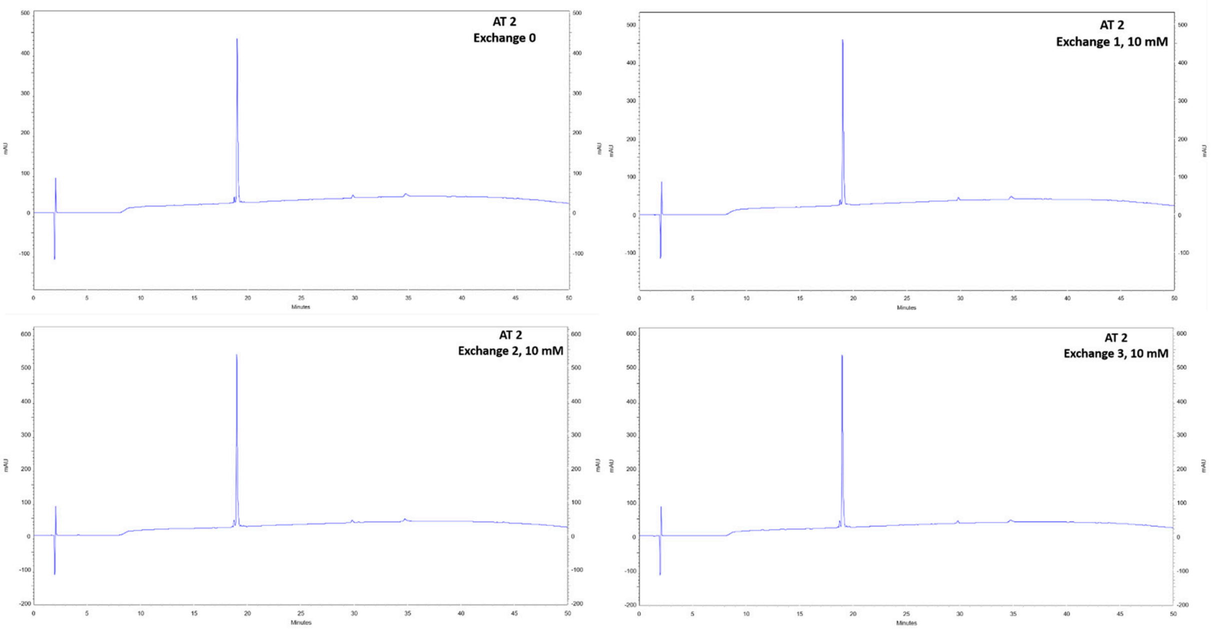

Figure S36: Purity of AT2 over three counterion exchanges with 10 mM HCl

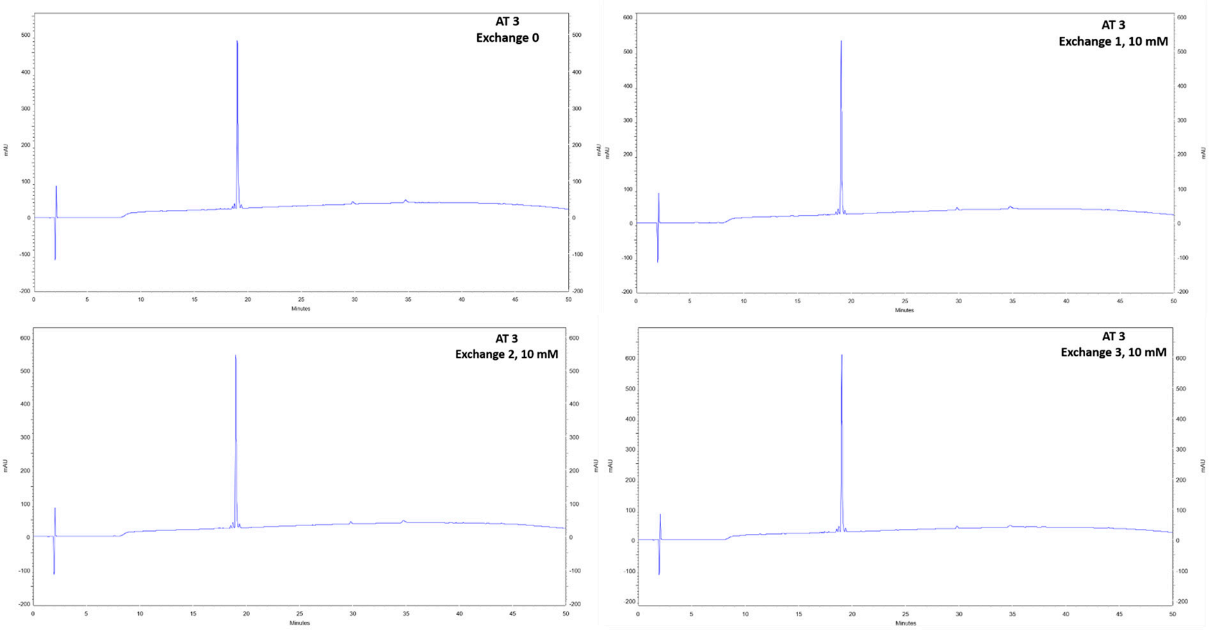

Figure S37: Purity of AT3 over three counterion exchanges with 10 mM HCl

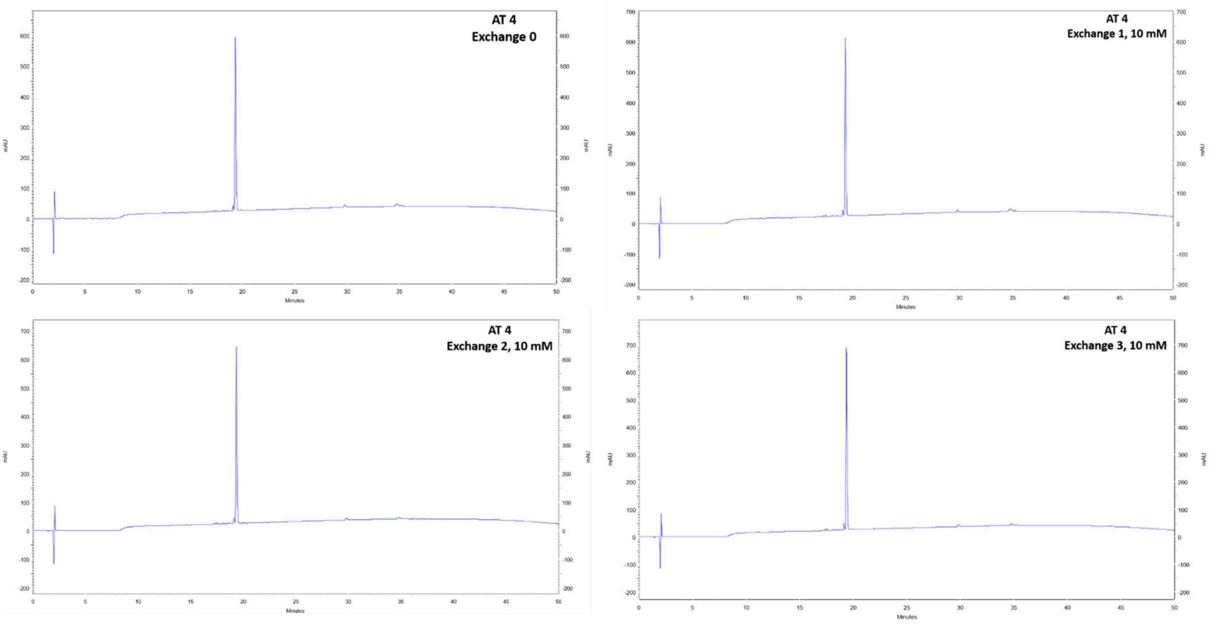

Figure S38: Purity of AT4 over three counterion exchanges with 10 mM HCl

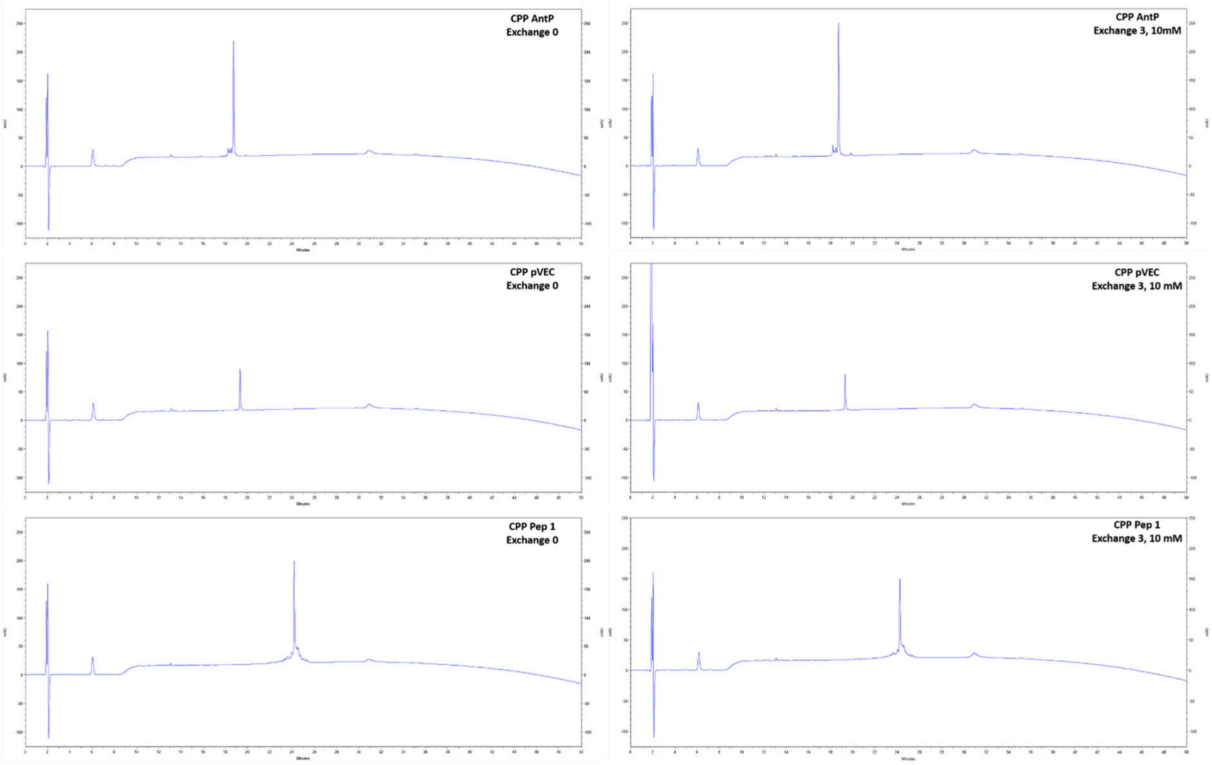

Figure S39: Purity of CPPs before and after three counterion exchanges with 10 mM HCl

Section S3.5: Additional Figures and Tables

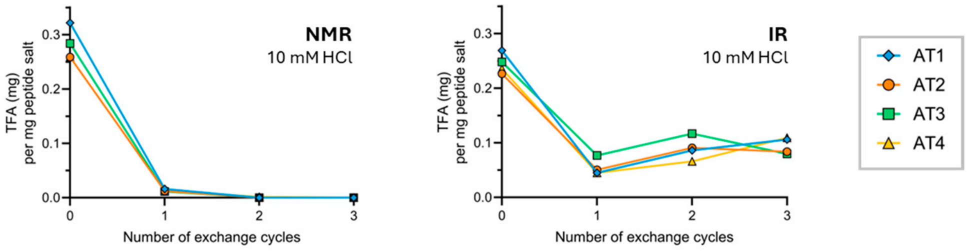

Figure S3.32: TFA per mg peptide salt determined by FT-IR and 19F-NMR for AT1-4

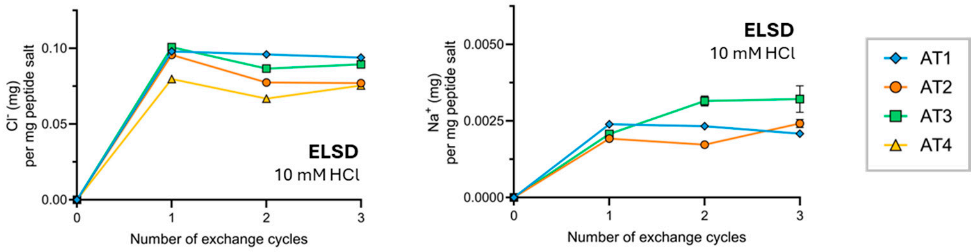

Figure S40: Cl and Na per mg peptide salt determined by HPLC-ELSD for AT1-4

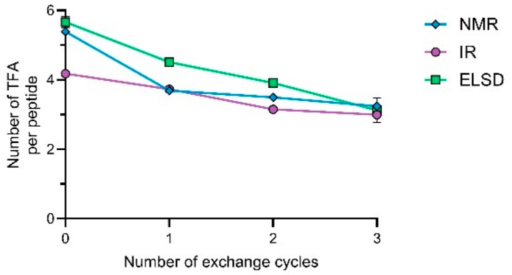

Figure S41: Comparison of calculations of number of TFA per peptide determined by 19F-NMR, FT-IR or HPLC-ELSD for AT1 exchanged with 0 mM HCl

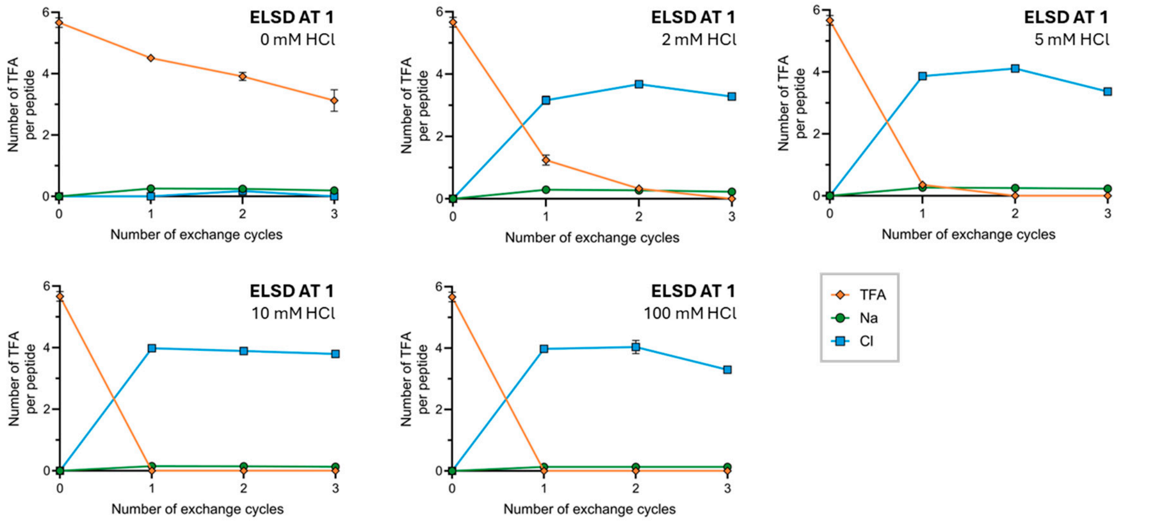

Figure S42: Number of TFA per peptide per exchange cycle for AT1 at different concentrations of HCl determined by HPLC-ELSD

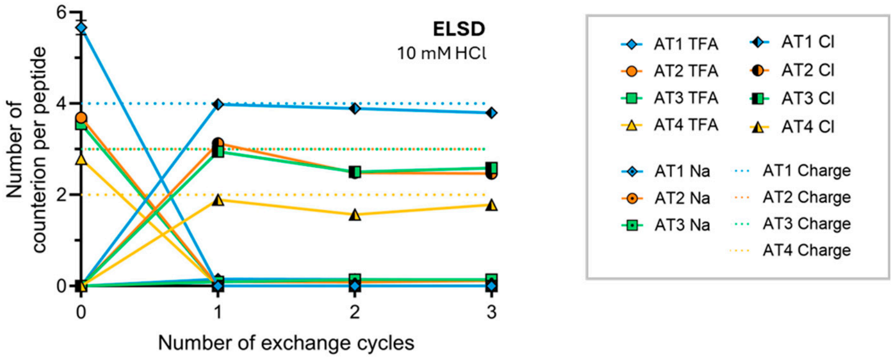

Figure S43: Number of counterions per exchange cycle for AT1-4 determined by HPLC-ELSD

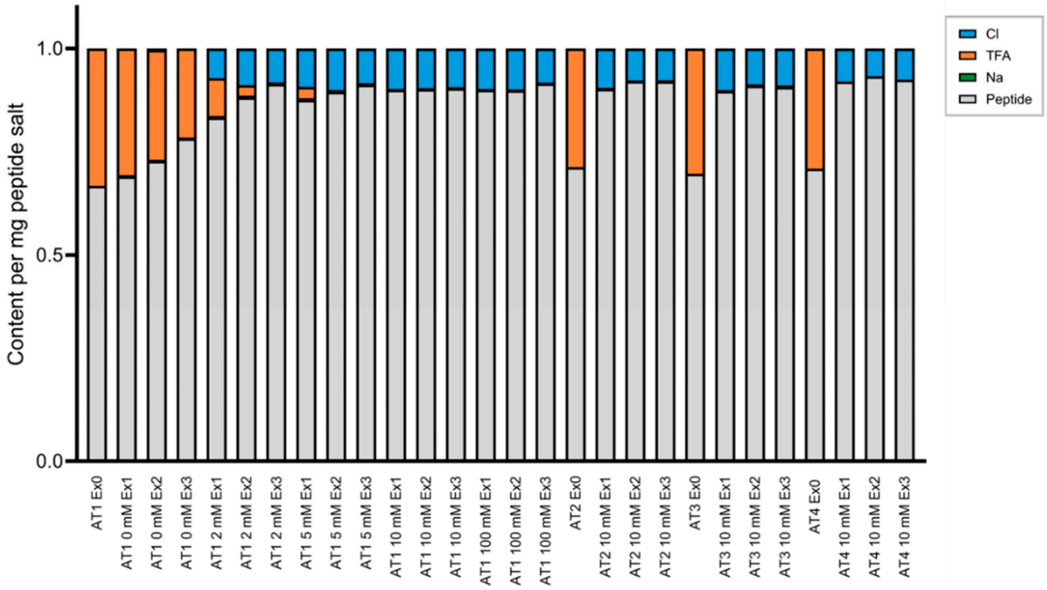

Figure S44: Content of counterion per mg peptide for AT1-4 at different concentrations of HCl over 3 exchange cycles

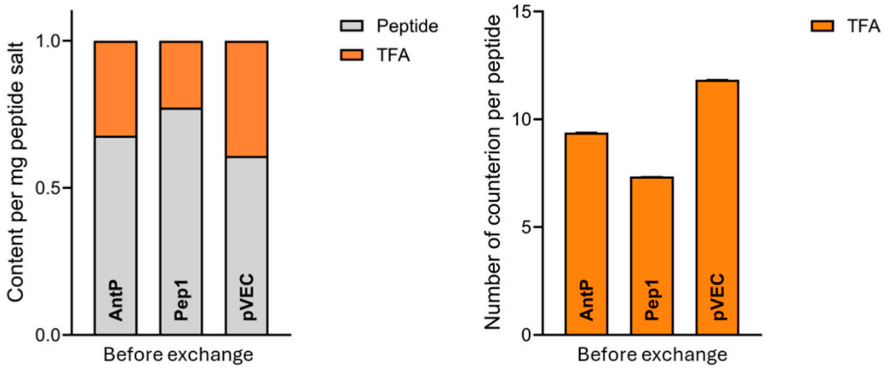

Figure S45: Content of TFA per mg peptide salt and number of TFA per peptide for AntP, Pep1 and pVEC

Table S3: TFA determination by IR

	Peptide	AT 1					AT 2	AT 3	AT 4
	Conc. aq. HCl for salt exchange (mM)	0	2	5	10	100	10	10	10
**Before**	Conc. of peptide salt (mg/mL)	4.03					4.62	4.77	4.75
	Max. Absorbance (10^{-3})	1.48					1.43	1.61	1.53
	Wavenumber at max. absorbance (cm^{-1})	1199.50					1201.50	1204.25	1204.75
	Conc. TFA (mg/mL)	1.08					1.05	1.18	1.12
	TFA per 1 mg peptide salt (mg)	0.27					0.23	0.25	0.24
**Ex change 1**	Conc. of peptide salt (mg/mL)	4.33	4.17	4.15	3.97	4.03	3.97	4.08	4.02
	Max. Absorbance (10^{-3})	1.46	0.85	0.49	0.25	0.30	0.28	0.44	0.26
	Wavenumber at max. absorbance (cm^{-1})	1200.00	1193.00	1199.50	1205.00	1208.50	1194.50	1194.75	1207.75
	Conc. TFA (mg/mL)	1.07	0.62	0.35	0.18	0.21	0.20	0.31	0.18
	TFA per 1 mg peptide salt (mg)	0.25	0.15	0.08	0.05	0.05	0.05	0.08	0.05
**Exchange 2**	Conc. of peptide salt (mg/mL)	4.23	3.90	4.33	4.47	3.82	4.72	4.03	4.02
	Max. Absorbance (10^{-3})	1.26	0.58	0.52	0.53	0.65	0.59	0.65	0.37
	Wavenumber at max. absorbance (cm^{-1})	1204.50	1190.00	1195.00	1195.00	1194.75	1194.75	1198.50	1194.50
	Conc. TFA (mg/mL)	0.92	0.42	0.38	0.38	0.47	0.43	0.47	0.26
	TFA per 1 mg peptide salt (mg)	0.22	0.11	0.09	0.09	0.12	0.09	0.12	0.07
**Exchange 3**	Conc. of peptide salt (mg/mL)	4.48	4.43	4.15	4.42	3.73	4.20	4.12	4.40
	Max. Absorbance (10^{-3})	1.28	0.60	0.58	0.64	0.43	0.49	0.46	0.66
	Wavenumber at max. absorbance (cm^{-1})	1204.25	1193.25	1194.50	1199.25	1190.25	1194.75	1190.75	1194.75
	Conc. TFA (mg/mL)	0.94	0.43	0.42	0.47	0.31	0.35	0.33	0.48
	TFA per 1 mg peptide salt (mg)	0.21	0.10	0.10	0.11	0.08	0.08	0.08	0.11

* Values highlighted in orange were detected below the LOQ of TFA

Table S4: TFA determination by NMR

	Peptide	AT 1					AT 2		AT 3		AT 4		AntP	Pep1	pVEC
	Conc. aq. HCl for salt exchange (mM)	0	2	5	10	100	0	10	0	10	0	10	10	10	10
**Before**	Conc. peptide salt (mg/mL)	2.02					2.31		2.38		2.38		3.23	2.25	2.17
	TFA signal shift (ppm)	75.4					75.4		75.5		75.5		75.5	75.5	75.4
	Conc. TFA (mg/mL) mean	0.6489					0.5977		0.6766		0.6151		1.0438	0.5110	0.8486
	Conc. TFA (mg/mL) std	0.0071					0.0062		0.0058		0.0105		0.0012	0.0004	0.0005
	TFA per 1 mg peptide salt mean (mg)	0.3218					0.2589		0.2839		0.2590		0.3228	0.2271	0.3917
	TFA per 1 mg peptide salt std (mg)	0.0035					0.0027		0.0024		0.0044		0.0004	0.0002	0.0002
**Exchange 1**	Conc. peptide salt (mg/mL)	2.17	2.08	2.08	1.98	2.02	2.08	1.98	2.50	2.04	2.47	2.01			
	TFA signal shift (ppm)	75.4	75.4	75.5	75.5	75.5	75.5	75.5	75.5	75.5	75.5	75.5			
	Conc. TFA (mg/mL) mean	0.5313	0.1596	0.0564	0.0065	0.0147	0.5034	0.0144	0.6830	0.0182	0.5693	0.0203			
	Conc. TFA (mg/mL) std	0.0045	0.0026	0.0010	0.0064	0.0096	0.0055	0.0009	0.0021	0.0009	0.0044	0.0013			
	TFA per 1 mg peptide salt mean (mg)	0.2452	0.0766	0.0272	0.0033	0.0073	0.2416	0.0072	0.2732	0.0089	0.2308	0.0101			
	TFA per 1 mg peptide salt std (mg)	0.0021	0.0012	0.0005	0.0032	0.0047	0.0027	0.0005	0.0009	0.0004	0.0018	0.0007			
**Exchange 2**	Conc. peptide salt (mg/mL)	2.12	1.95	2.17	2.23	1.91	2.43	2.36	2.32	2.02	2.35	2.01			
	TFA signal shift (ppm)	75.4	75.5	75.5	n.a.	n.a.	75.5	n.a.	75.5	n.a.	75.5	75.5			
	Conc. TFA (mg/mL) mean	0.4980	0.0275	0.0025	0.0000	0.0000	0.5324	0.0000	0.5836	0.0000	0.5526	0.0030			
	Conc. TFA (mg/mL) std	0.0010	0.0015	0.0001	0.0000	0.0000	0.0026	0.0000	0.0039	0.0000	0.0020	0.0001			
	TFA per 1 mg peptide salt mean (mg)	0.2353	0.0141	0.0011	0.0000	0.0000	0.2188	0.0000	0.2519	0.0000	0.2352	0.0015			
	TFA per 1 mg peptide salt std (mg)	0.0005	0.0008	0.0001	0.0000	0.0000	0.0011	0.0000	0.0017	0.0000	0.0008	0.0001			
**E**	Conc. peptide salt (mg/mL)	2.24	2.22	2.08	2.21	1.87	2.18	2.10	2.15	2.06	2.35	2.20	1.05	1.28	0.80

	TFA signal shift (ppm)	75.4	75.5	n.a.	n.a.	n.a.	75.5	n.a.	75.5	n.a.	75.5	n.a.	n.a.	n.a.	n.a.
	Conc. TFA (mg/mL) mean	0.4975	0.0048	0.0000	0.0000	0.0000	0.4372	0.0000	0.5371	0.0000	0.5284	0.0000	0.0000	0.0000	0.0000
	Conc. TFA (mg/mL) std	0.0087	0.0006	0.0000	0.0000	0.0000	0.0048	0.0000	0.0027	0.0000	0.0025	0.0000	0.0000	0.0000	0.0000
	TFA per 1 mg peptide salt mean (mg)	0.2219	0.0021	0.0000	0.0000	0.0000	0.2003	0.0000	0.2498	0.0000	0.2248	0.0000	0.0000	0.0000	0.0000
	TFA per 1 mg peptide salt std (mg)	0.0039	0.0003	0.0000	0.0000	0.0000	0.0022	0.0000	0.0012	0.0000	0.0011	0.0000	0.0000	0.0000	0.0000

* Values highlighted in orange were detected below the LOQ of TFA

Table S5: TFA, Cl and Na determination by HPLC-ELSD

	Peptide	AT 1					AT 2	AT 3	AT 4
	Conc. aq. HCl for salt exchange (mM)	0	2	5	10	100	10	10	10
Before	Conc. peptide salt (mg/mL)	0.81					0.92	0.95	0.95
Before	**Injection volume (µL)**	**20**					**20**	**20**	**20**
Before	Conc. TFA (µg/mL) mean	268.36					264.98	289.13	276.24
Before	Conc. TFA (µg/mL) std	6.53					4.80	5.41	2.37
Before	TFA per 1 mg peptide salt mean (mg)	0.3327					0.2870	0.3033	0.2908
Before	TFA per 1 mg peptide salt std (mg)	0.0081					0.0052	0.0057	0.0025
Before	Conc. Cl (µg/mL) mean	0.00					0.00	0.00	0.00
Before	Conc. Cl (µg/mL) std	0.00					0.00	0.00	0.00
Before	Cl per 1 mg peptide salt mean (mg)	0.0000					0.0000	0.0000	0.0000
Before	Cl per 1 mg peptide salt std (mg)	0.0000					0.0000	0.0000	0.0000
Before	Conc. Na (µg/mL) mean	0.00					0.00	0.00	0.00
Before	Conc. Na (µg/mL) std	0.00					0.00	0.00	0.00
Before	Na per 1 mg peptide salt mean (mg)	0.0000					0.0000	0.0000	0.0000
Before	Na per 1 mg peptide salt std (mg)	0.0000					0.0000	0.0000	0.0000
Exchange 1	Conc. peptide salt (mg/mL)	0.87	0.83	0.83	0.79	0.81	0.79	0.82	0.80
Exchange 1	**Injection volume (µL)**	**20**	**20**	**20**	**20**	**20**	**20**	**20**	**20**
Exchange 1	Conc. TFA (µg/mL) mean	245.53	75.87	22.97	0.00	0.00	0.00	0.00	0.00
Exchange 1	Conc. TFA (µg/mL) std	5.48	9.82	0.71	0.00	0.00	0.00	0.00	0.00
Exchange 1	TFA per 1 mg peptide salt mean (mg)	0.2833	0.0910	0.0277	0.0000	0.0000	0.0000	0.0000	0.0000
Exchange 1	TFA per 1 mg peptide salt std (mg)	0.0063	0.0118	0.0009	0.0000	0.0000	0.0000	0.0000	0.0000
Exchange 1	Conc. Cl (µg/mL) mean	0.00	59.96	76.89	77.72	78.94	75.87	82.29	64.05
Exchange 1	Conc. Cl (µg/mL) std	0.00	2.40	0.73	0.82	0.14	1.46	1.23	0.45
Exchange 1	Cl per 1 mg peptide salt mean (mg)	0.0000	0.0720	0.0926	0.0980	0.0979	0.0956	0.1008	0.0797
Exchange 1	Cl per 1 mg peptide salt std (mg)	0.0000	0.0029	0.0009	0.0010	0.0002	0.0018	0.0015	0.0006
Exchange 1	Conc. Na (µg/mL) mean	2.71	3.58	3.44	1.90	1.73	1.53	1.69	n/a
Exchange 1	Conc. Na (µg/mL) std	0.08	0.25	0.12	0.07	0.07	0.01	0.04	n/a
Exchange 1	Na per 1 mg peptide salt mean (mg)	0.0031	0.0043	0.0041	0.0024	0.0021	0.0019	0.0021	n/a

	Na per 1 mg peptide salt std (mg)	0.000 1	0.000 3	0.000 1	0.000 1	0.000 1	0.000 0	0.000 0	n/a

Continued on next page

**Exchange 2**	Conc. peptide salt (mg/mL)	0.85	0.78	0.87	0.89	0.76	0.94	0.81	0.80
	**Injection volume (µL)**	**20**	**20**	**20**	**20**	**20**	**20**	**20**	**20**
	Conc. TFA (µg/mL) mean	215.37	19.89	0.00	0.00	0.00	0.00	0.00	0.00
	Conc. TFA (µg/mL) std	6.78	0.99	0.00	0.00	0.00	0.00	0.00	0.00
	TFA per 1 mg peptide salt mean (mg)	0.2544	0.0255	0.0000	0.0000	0.0000	0.0000	0.0000	0.0000
	TFA per 1 mg peptide salt std (mg)	0.0080	0.0013	0.0000	0.0000	0.0000	0.0000	0.0000	0.0000
	Conc. Cl (µg/mL) mean	2.96	69.12	87.24	85.76	75.70	73.06	69.89	53.72
	Conc. Cl (µg/mL) std	0.18	2.08	1.89	0.28	4.08	0.26	1.31	0.58
	Cl per 1 mg peptide salt mean (mg)	0.0035	0.0886	0.1007	0.0960	0.0992	0.0774	0.0866	0.0669
	Cl per 1 mg peptide salt std (mg)	0.0002	0.0027	0.0022	0.0003	0.0053	0.0003	0.0016	0.0007
	Conc. Na (µg/mL) mean	2.71	3.34	3.45	2.08	1.60	1.63	2.54	n/a
	Conc. Na (µg/mL) std	0.04	0.11	0.10	0.06	0.04	0.08	0.13	n/a
	Na per 1 mg peptide salt mean (mg)	0.0032	0.0043	0.0040	0.0023	0.0021	0.0017	0.0032	n/a
	Na per 1 mg peptide salt std (mg)	0.0000	0.0001	0.0001	0.0001	0.0001	0.0001	0.0002	n/a
**Exchange 3**	Conc. peptide salt (mg/mL)	0.90	0.89	0.83	0.88	0.75	0.84	0.82	0.88
	**Injection volume (µL)**	**20**	**20**	**20**	**20**	**20**	**20**	**20**	**20**
	Conc. TFA (µg/mL) mean	192.87	0.00	0.00	0.00	0.00	0.00	0.00	0.00
	Conc. TFA (µg/mL) std	21.01	0.00	0.00	0.00	0.00	0.00	0.00	0.00
	TFA per 1 mg peptide salt mean (mg)	0.2151	0.0000	0.0000	0.0000	0.0000	0.0000	0.0000	0.0000
	TFA per 1 mg peptide salt std (mg)	0.0234	0.0000	0.0000	0.0000	0.0000	0.0000	0.0000	0.0000
	Conc. Cl (µg/mL) mean	0.00	72.76	69.74	82.94	61.62	64.68	73.65	66.43
	Conc. Cl (µg/mL) std	0.00	0.92	0.59	1.02	0.57	0.41	0.62	1.13
	Cl per 1 mg peptide salt mean (mg)	0.0000	0.0821	0.0840	0.0939	0.0825	0.0770	0.0894	0.0755
	Cl per 1 mg peptide salt std (mg)	0.0000	0.0010	0.0007	0.0012	0.0008	0.0005	0.0008	0.0013
	Conc. Na (µg/mL) mean	2.38	3.26	3.15	1.84	1.62	2.03	2.65	n/a
	Conc. Na (µg/mL) std	0.03	0.03	0.04	0.04	0.01	0.11	0.36	n/a
	Na per 1 mg peptide salt mean (mg)	0.0027	0.0037	0.0038	0.0021	0.0022	0.0024	0.0032	n/a
	Na per 1 mg peptide salt std (mg)	0.0000	0.0000	0.0000	0.0000	0.0000	0.0001	0.0004	n/a

* Cl concentrations highlighted in blue were determined from measurements with 5 µL injection volume, ensuring signals in the calibrated range

* Values highlighted in orange were detected below the LOQ of the corresponding counterion

Table S6: Calculations number of counterions per peptide for IR, NMR and HPLC-ELSD

	Peptide	MW (Da)	Conc. aq. HCl for salt exchange (mM)	Number of exchange cycles	Peptide per 1 mg peptide salt mean (mmol)	Peptide per mg peptide salt std (mmol)	TFA per 1 mg peptide salt mean (mmol)	TFA per 1 mg peptide salt std (mmol)	Cl per 1 mg peptide salt mean (mmol)	Cl per 1 mg peptide salt std (mmol)	Na per 1 mg peptide salt mean (mmol)	Na per 1 mg peptide salt std (mmol)	TFA per peptide mean	TFA per peptide std	Cl per peptide mean	Cl per peptide std	Na per peptide mean	Na per peptide std
IR	AT 1	1296.5		before	5.6E-04		2.4E-03						4.18					
			0	Ex1	5.8E-04		2.2E-03						3.73					
				Ex2	6.0E-04		1.9E-03						3.15					
				Ex3	6.1E-04		1.8E-03						3.00					
NMR	AT 1	1296.5		before	5.2E-04	2.7E-06	2.8E-03	3.08E-05					5.39	0.07				
			0	Ex1	5.8E-04	1.58E-06	2.2E-03	1.8E-05					3.69	0.03				
				Ex2	5.9E-04	3.54E-07	2.1E-03	4.02E-06					3.50	0.01				
				Ex3	6.0E-04	2.98E-06	1.9E-03	3.39E-05					3.24	0.06				
	AT 2	1046.2		before	7.1E-04	2.56E-06	2.3E-03	2.35E-05					3.21	0.04				
			0	Ex1	7.2E-04	2.54E-06	2.1E-03	2.33E-05					2.92	0.03				

				Ex2	7.5E-04	1.01E-06	1.9E-03	9.26E-06					2.57	0.01				
				Ex3	7.6E-04	2.09E-06	1.8E-03	1.92E-05					2.30	0.03				
	AT 3	931.1		before	7.7E-04	2.63E-06	2.5E-03	2.15E-05					3.24	0.03				
			0	Ex1	7.8E-04	9.18E-07	2.4E-03	7.5E-06					3.07	0.01				
				Ex2	8.0E-04	1.82E-06	2.2E-03	1.49E-05					2.75	0.02				
				Ex3	8.1E-04	1.32E-06	2.2E-03	1.08E-05					2.72	0.01				
	AT 4	774.9		before	9.6E-04	5.7E-06	2.3E-03	3.88E-05					2.38	0.04				
			0	Ex1	9.9E-04	2.32E-06	2.0E-03	1.57E-05					2.04	0.02				
				Ex2	9.9E-04	1.08E-06	2.1E-03	7.32E-06					2.09	0.01				
				Ex3	1.0E-03	1.37E-06	2.0E-03	9.31E-06					1.97	0.01				
	AntP	2245.8	10	before	3.0E-04	1.71E-07	2.8E-03	3.37E-06					9.39	0.01				
	Pep1	2848.3	10	before	2.7E-04	6.73E-08	2.0E-03	1.68E-06					7.34	0.01				
	pVEC	2096.6	10	before	2.9E-04	1.17E-07	3.4E-03	2.15E-06					11.84	0.01				
ELSD	AT 1	1296.5		before	5.1E-04	6.2E-06	2.9E-03	7.1E-05	0	0	0	0	5.67	0.15	0	0	0	0
			0	Ex1	5.5E-04	4.9E-06	2.5E-03	5.5E-05	0	0	1.4E-04	3.8E-06	4.51	0.11	0	0	0.25	0.01

				Ex2	5.7E-04	6.2E-06	2.2E-03	7.0E-05	9.9E-05	6.1E-06	1.4E-04	2.1E-06	3.91	0.13	0.17	0.01	0.24	0.00
				Ex3	6.0E-04	1.8E-05	1.9E-03	2.1E-04	0	0	1.2E-04	1.6E-06	3.13	0.35	0	0	0.19	0.01
			2	Ex1	6.4E-04	9.4E-06	8.0E-04	1.0E-04	2.0E-03	8.1E-05	1.9E-04	1.3E-05	1.24	0.16	3.16	0.13	0.29	0.02
				Ex2	6.8E-04	2.3E-06	2.2E-04	1.1E-05	2.5E-03	7.5E-05	1.9E-04	6.1E-06	0.33	0.02	3.68	0.11	0.27	0.01
				Ex3	7.1E-04	8.0E-07	0	0	2.3E-03	2.9E-05	1.6E-04	1.4E-06	0	0	3.28	0.04	0.23	0.00
			5	Ex1	6.8E-04	9.5E-07	2.4E-04	7.5E-06	2.6E-03	2.5E-05	1.8E-04	6.4E-06	0.36	0.01	3.87	0.04	0.27	0.01
				Ex2	6.9E-04	1.7E-06	0	0	2.8E-03	6.1E-05	1.7E-04	5.0E-06	0	0	4.11	0.09	0.25	0.01
				Ex3	7.0E-04	5.5E-07	0	0	2.4E-03	2.0E-05	1.7E-04	2.0E-06	0	0	3.37	0.03	0.23	0.00
			10	Ex1	6.9E-04	8.0E-07	0	0	2.8E-03	2.9E-05	1.0E-04	4.0E-06	0	0	3.98	0.04	0.15	0.01
				Ex2	7.0E-04	2.4E-07	0	0	2.7E-03	8.7E-06	1.0E-04	3.0E-06	0	0	3.89	0.01	0.15	0.00
				Ex3	7.0E-04	8.9E-07	0	0	2.6E-03	3.2E-05	9.1E-05	2.1E-06	0	0	3.80	0.05	0.13	0.00
			100	Ex1	6.9E-04	1.5E-07	0	0	2.8E-03	4.9E-06	9.3E-05	4.0E-06	0	0	3.98	0.01	0.13	0.01
				Ex2	6.9E-04	4.1E-06	0	0	2.8E-03	1.5E-04	9.1E-05	2.4E-06	0	0	4.04	0.22	0.13	0.00
				Ex3	7.1E-04	5.9E-07	0	0	2.3E-03	2.1E-05	9.4E-05	7.2E-07	0	0	3.30	0.03	0.13	0.00
	AT 2	1046.2		before	6.8E-04	5.0E-06	2.5E-03	4.6E-05	0	0	0	0	3.69	0.07	0	0	0	0

			10	Ex1	8.6E-04	1.8E-06	0	0	2.7E-03	5.2E-05	8.4E-05	4.9E-07	0	0	3.13	0.06	0.10	0.00
				Ex2	8.8E-04	2.7E-07	0	0	2.2E-03	7.8E-06	7.5E-05	3.7E-06	0	0	2.48	0.01	0.09	0.00
				Ex3	8.8E-04	4.8E-07	0	0	2.2E-03	1.4E-05	1.1E-04	5.6E-06	0	0	2.47	0.02	0.12	0.01
	AT 3	931.1		before	7.5E-04	6.1E-06	2.7E-03	5.0E-05	0	0	0	0	3.55	0.07	0	0	0	0
			10	Ex1	9.6E-04	1.6E-06	0	0	2.8E-03	4.3E-05	9.0E-05	2.1E-06	0	0	2.95	0.04	0.09	0.00
				Ex2	9.8E-04	1.7E-06	0	0	2.4E-03	4.6E-05	1.4E-04	7.1E-06	0	0	2.50	0.05	0.14	0.01
				Ex3	9.7E-04	9.3E-07	0	0	2.5E-03	2.1E-05	1.4E-04	1.9E-05	0	0	2.59	0.02	0.14	0.02
	AT 4	774.9		before	9.2E-04	3.2E-06	2.6E-03	2.2E-05	0	0	0	0	2.79	0.03	0	0	0	0
			10	Ex1	1.2E-03	7.3E-07	0	0	2.2E-03	1.6E-05	0	0	0	0	1.89	0.01	0	0
				Ex2	1.2E-03	9.2E-07	0	0	1.9E-03	2.0E-05	0	0	0	0	1.57	0.02	0	0
				Ex3	1.2E-03	1.7E-06	0	0	2.1E-03	3.6E-05	0	0	0	0	1.78	0.03	0	0

Section S4: **Liposomal Assay**

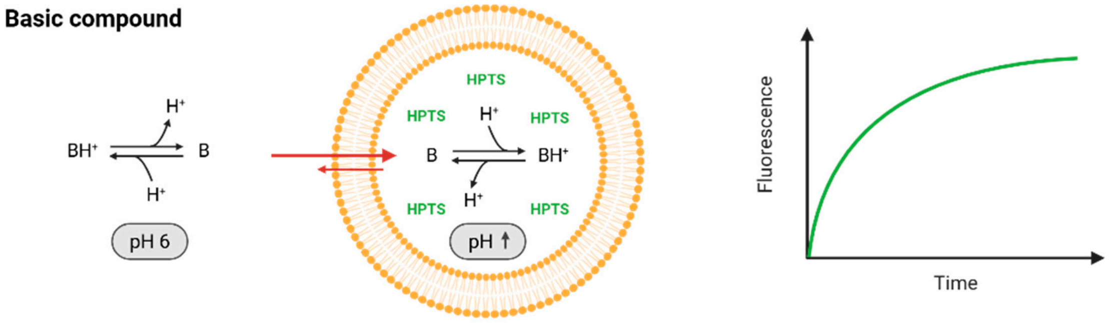

Figure S46: General mechanism of the liposomal assay for a basic compound

Table S7: Size of liposomes used in liposomal assay

	**Size liposomes (nm)**	**PolyIndex**
	144.1	0.175
	148.6	0.151
	146.5	0.177
	148.3	0.180
**Average**	**146.9**	**0.171**
**std**	**1.8**	**0.012**

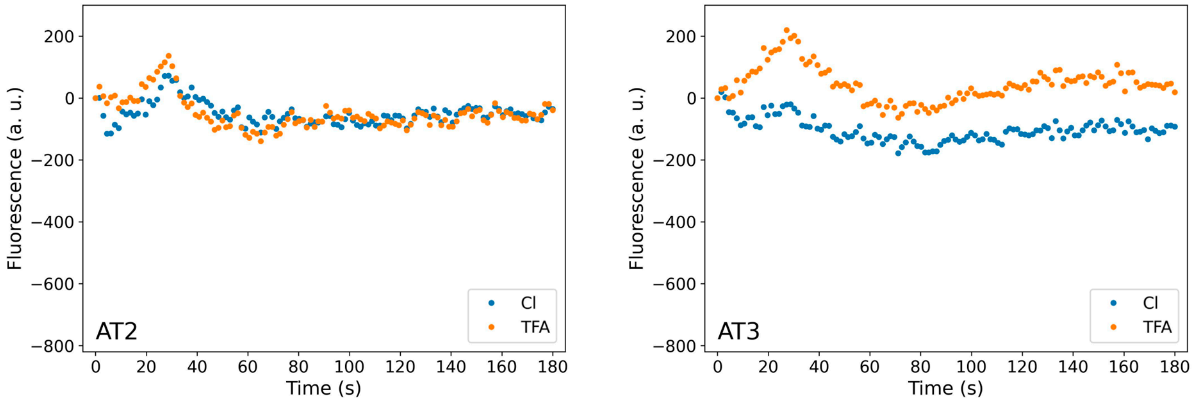

Figure S47: Permeation kinetics of AT2 and AT3 as TFA and Cl salt

Table S8: pH measurements of AT1-4 peptide salt in aq. solution after exchange and lyophilization

	Peptide	AT 1					AT2		AT3		AT4	
	Conc. aq. HCl for salt exchange (mM)	0	2	5	10	100	0	10	0	10	0	10
Before exchange	Conc. peptide salt (mg/mL)	2.02					2.31		2.38		2.38	
	pH	2.59					2.63		2.63		2.39	
After exchange 1	Conc. peptide salt (mg/mL)	2.17	2.08	2.08	1.98	2.02	2.08	1.98	2.50	2.04	2.47	2.01
	pH	2.92	2.78	2.79	2.73	2.69	2.88	2.7	3.05	2.86	3.03	2.87
After exchange 2	Conc. peptide salt (mg/mL)	2.12	1.95	2.17	2.23	1.91	2.43	2.36	2.32	2.02	2.35	2.01
	pH	2.95	2.81	2.77	2.73	2.66	2.94	2.69	3.08	2.86	3.08	2.91
After exchange 3	Conc. peptide salt (mg/mL)	2.24	2.22	2.08	2.21	1.87	2.18	2.10	2.15	2.06	2.35	2.20
	pH	3	2.76	2.78	2.73	2.75	3.01	2.7	3.26	2.85	3.22	2.86

Python script for liposomal assay data evaluation:

```
import os
import pandas as pd
import matplotlib.pyplot as plt
import numpy as np
from scipy.optimize import curve_fit

# Import data
folderpath = 'Y:\LiposomeAssay'
file_name = 'Data.xlsx'
full_path = os.path.join(folderpath, file_name)
sheet_names = sorted(pd.ExcelFile(full_path, engine="openpyxl").sheet_names)


# Summarize samples in a dictionary
sample_groups = {}
for sheet in sheet_names:
    sample_name = sheet. rsplit('_', 1)[0]
    if sample_name not in sample_groups:
        sample_groups[sample_name] = []
    sample_groups[sample_name].append(sheet)

# Introduce variables
samples = []
counterions = []
curves_x = []
```

```
curves_y = []
means_x = []
means_y = []

# Calculate mean value of three replicate samples
for sample, sheets in sample_groups.items():

    xs = []
    ys = []

    if 'TFA' in sample:
        counterion = 'TFA'
    else:
        counterion = 'Cl'
    counterions.append(counterion)

    # Read data from Excel, combine replicates of the same sample
    for sheet in sheets:
        df = pd.read_excel(
            full_path,
            sheet_name=sheet,
            engine="openpyxl",
        )

        # Import x and y values
        time = pd.to_numeric(df.iloc[:, 1]).dropna().to_numpy()
        signal = pd.to_numeric(df.iloc[:, 3]).dropna().to_numpy()

        # Normalize start point of curve to 0
        signal_normalized = signal - signal[0]

        xs.append(time)
        ys.append(signal_normalized)

        plt.plot(time, signal_normalized, label=sheet)


    # Interpolation-based mean curve, allowing a mean value for data curves without the same number
of data points (depends on sample location on plate reader, approx. every 1.5 second)
    mean_x_axis = np.linspace(0, 180, num=120) # values for interpolation
    ys_interp = [np.interp(mean_x_axis, xs[i], ys[i]) for i in range(len(xs))] # interpolation
    ys_interp = np.array(ys_interp)
    mean_y_axis = np.mean(ys_interp, axis=0) # mean curve
    std_y_axis = np.std(ys_interp, axis=0) # standard deviation for each point

    # Update variables and store for further processing
    samples.append(sample)
    curves_x.append(xs)
    curves_y.append(ys)
    means_x.append(mean_x_axis)
    means_y.append(mean_y_axis)
```

```
# Find the blank curve to substract from all samples
blank_index = None
for i, name in enumerate(samples):
    if name.lower() == "blank":
        blank_index = i
        break

blank_x = means_x[blank_index]
blank_y = means_y[blank_index]

# Function for double exponential fit
def fit_double_exponential(x, a1, b1, a2, b2):
    return a1 * np.exp(-b1 * (x)) + a2 * np.exp(-b2 * (x))

fit_initial_guess = [100, 0.05, 10, 0.001]

size_liposomes_nm = 146.875
size_liposomes_nm_std = 1.79217


for i in range(len(samples)):
    peptide = samples[i]
    if i == blank_index: # Skip blank
        r_squared = rmse = normalized_rmse = None
        a1 = b1 = a2 = b2 = None
        a1_err = b1_err = a2_err = b2_err = None
    else:
        y = means_y[i]
        corrected_y = y - blank_y  #Blank correction
        y0 = np.min(corrected_y)
        zero_normalized_y = corrected_y - y0 # normalize minimum in curve to zero to improve fitting

        #Data for nonlinear regression
        x_data = means_x[i]
        y_data = zero_normalized_y

        try:
            # Fit the curve
            popt, pcov = curve_fit(fit_double_exponential, x_data, y_data, p0=fit_initial_guess)

            # predict values and determine R squared, RMSE, normalized RMSE
            y_pred = fit_double_exponential(x_data, *popt)
            residuals = y_data - y_pred
            ss_res = np.sum(residuals**2)
            ss_tot = np.sum((y_data - np.mean(y_data))**2)
            r_squared = 1 - (ss_res / ss_tot)
            rmse = np.sqrt(ss_res / len(y_data))
            normalized_rmse = rmse / (np.max(y_data)-np.min(y_data))

            # Extract fitted parameters and standard errors
            a1, b1, a2, b2 = popt
            a1_err, b1_err, a2_err, b2_err = np.sqrt(np.diag(pcov))
```

```
        if b1 > b2:
            ka = b1
            ka_std = b1_err
        else:
            ka = b2
            ka_std = b2_err
        # Calculate Permapp from measured parameters
        Permapp = ka * size_liposomes_nm / 6 * 1e-7
        log_Permapp = np.log10(Permapp)
        dPermapp = (1e-7 / 6) * np.sqrt((ka * size_liposomes_nm_std)**2 + (size_liposomes_nm *
ka_std)**2) #Calculate standard deviation by error propagation
        log_dPermapp = dPermapp / (Permapp * np.log(10)) #Calculate standard deviation by error
propagation


    except Exception as e:
        r_squared = rmse = normalized_rmse = None
        a1 = b1 = a2 = b2 = None
        a1_err = b1_err = a2_err = b2_err = None
        Permapp = log_Permapp = dPermapp = log_dPermapp = None


    x = np.linspace(0, 180, num=120)

    x_fit = np.linspace(0, 180, 900)

    color_map = {
        'HCl': 'tab:blue',
        'Cl': 'tab:blue',
        'TFA': 'tab:orange'
    }

    plt.figure(figsize=(7, 5))

    label = counterions[i]
    y = zero_normalized_y


    y_fit = fit_double_exponential(x_fit, a1, b1, a2, b2)

    y_zeroed = y - y[0]
    y_fit_zeroed = y_fit - y[0]


    color = color_map[label]
    plt.plot(x, y_zeroed, 'o', ms= 4, label=f'{label}', color = color)
    if normalized_rmse <= 0.1:
        plt.plot(x_fit, y_fit_zeroed, '-', label=f'{label} fit', color = color, linewidth = 2.5)

  plt.text(0, -780, f"{peptide}", fontsize=20)
  plt.xlabel("Time (s)", fontsize = 16)
  plt.ylabel("Fluorescence (a. u.)", fontsize = 16)
```

```
plt.ylim(-820, 300)
plt.yticks(ticks= range(-800, 300, 200), fontsize = 14)
plt.xlim(-5, 185)
plt.xticks(ticks=range(0, 200, 20), fontsize = 14)
plt.legend(fontsize = 14)
plt.tight_layout()
plt.savefig(f"{peptide}.jpg", dpi = 300)
plt.show()
```

Table S9: Parameters of biexponential curve fitting for all liposomal permeation assay experiments

Sample	R^2	RMSE	RMSE normalized *	A	A std	k_a	k_a std	B	B std	k_B	k_B std	$Perm_{app}$	$Perm_{app}$ std	log $Perm_{app}$	log $Perm_{app}$ std
**HCl**	0.743	58.463	0.143	332.507	2.406E+08	0.014	6.914	30.630	2.406E+08	0.014	71.332				
**TFA**	0.739	25.609	0.133	192.457	9.197	0.018	0.002	0.309	0.520	-0.030	0.010				
**AT1 Cl**	0.187	19.864	0.192	46.946	9.518	0.037	0.019	18.376	6.418	-0.007	0.002				
**AT1 TFA**	0.496	22.857	0.108	173.214	19.744	0.206	0.042	52.351	4.141	-0.004	0.001				
**AT2 Cl**	0.140	33.573	0.180	96.922	9.633	0.008	0.004	0.670	2.729	-0.024	0.022				
**AT2 TFA**	0.421	38.835	0.141	184.045	13.209	0.017	0.004	5.836	7.887	-0.016	0.008				
**AT3 Cl**	0.603	24.238	0.122	164.316	9.731	0.026	0.004	11.540	5.803	-0.012	0.003				
**AT3 TFA**	0.184	51.385	0.181	163.250	17.854	0.013	0.007	9.551	17.484	-0.014	0.010				
**AT4 Cl**	0.863	35.482	0.108	355.626	13.129	0.020	0.002	0.480	2.127	-0.022	0.026				
**AT4 TFA**	0.695	33.651	0.139	4005.151	1.153E+08	0.006	2.467	-3798.359	1.153E+08	0.006	2.566				
**AntP Cl**	0.939	18.459	**0.047**	360.572	8.705	0.040	0.002	8.320	2.528	-0.013	0.002	9.692E-08	4.930E-09	-7.014	0.022
**Antp TFA**	0.967	16.747	**0.040**	450.559	8.459	0.046	0.002	3.784	1.310	-0.017	0.002	1.133E-07	3.984E-09	-6.946	0.015
**Pep1 Cl**	0.974	21.626	**0.031**	690.556	11.484	0.052	0.001	8.584	1.725	-0.016	0.001	1.279E-07	3.969E-09	-6.893	0.013

**Pep1 TFA**	0.874	23.584	**0.070**	272.940	14.708	0.078	0.007	17.001	1.989	-0.015	0.001	1.907E-07	1.701E-08	-6.720	0.039
**pVEC Cl**	0.603	22.238	0.162	46.245	10.000	0.035	0.018	17.793	4.231	-0.011	0.002				
**pVEC TFA**	0.793	17.760	**0.081**	185.778	9.432	0.050	0.006	17.025	4.806	-0.006	0.002	1.221E-07	1.355E-08	-6.913	0.048

* Samples with normalized RSME < 0.1 highlighted
